# Caspase-10 Negatively Regulates Caspase-8-Mediated Cell Death, Switching the Response to CD95L in Favor of NF-κB Activation and Cell Survival

**DOI:** 10.1016/j.celrep.2017.04.010

**Published:** 2017-04-25

**Authors:** Sebastian Horn, Michelle A. Hughes, Ramon Schilling, Carsten Sticht, Tencho Tenev, Michaela Ploesser, Pascal Meier, Martin R. Sprick, Marion MacFarlane, Martin Leverkus

**Affiliations:** 1 Section of Molecular Dermatology, Department of Dermatology, Venereology, and Allergology, Medical Faculty Mannheim, University of Heidelberg, Theodor-Kutzer-Ufer 1-3, 68167 Mannheim, Germany; 2 MRC Toxicology Unit, Hodgkin Building, PO Box 138, Lancaster Road, Leicester LE1 9HN, UK; 3 Center for Medical Research, Medical Faculty Mannheim, University of Heidelberg, Theodor-Kutzer-Ufer 1-3, 68167 Mannheim, Germany; 4 The Breakthrough Toby Robins Breast Cancer Research Centre, Institute of Cancer Research, Mary-Jean Mitchell Green Building, Chester Beatty Laboratories, Fulham Road, London SW3 6JB, UK; 5 Heidelberg Institute for Stem Cell Technology and Experimental Medicine (HI-STEM gGmbH), Im Neuenheimer Feld 280, 69120 Heidelberg, Germany; 6 Department of Dermatology and Allergology, Medical Faculty of the RWTH Aachen, Pauwelsstraße 30, 52074 Aachen, Germany

**Keywords:** caspase-10, caspase-8, cFLIP, cell death, DISC, NF-κB, CD95

## Abstract

Formation of the death-inducing signaling complex (DISC) initiates extrinsic apoptosis. Caspase-8 and its regulator cFLIP control death signaling by binding to death-receptor-bound FADD. By elucidating the function of the caspase-8 homolog, caspase-10, we discover that caspase-10 negatively regulates caspase-8-mediated cell death. Significantly, we reveal that caspase-10 reduces DISC association and activation of caspase-8. Furthermore, we extend our co-operative/hierarchical binding model of caspase-8/cFLIP and show that caspase-10 does not compete with caspase-8 for binding to FADD. Utilizing caspase-8-knockout cells, we demonstrate that caspase-8 is required upstream of both cFLIP and caspase-10 and that DISC formation critically depends on the scaffold function of caspase-8. We establish that caspase-10 rewires DISC signaling to NF-κB activation/cell survival and demonstrate that the catalytic activity of caspase-10, and caspase-8, is redundant in gene induction. Thus, our data are consistent with a model in which both caspase-10 and cFLIP coordinately regulate CD95L-mediated signaling for death or survival.

## Highlights

• Caspase-10 negatively regulates DISC-mediated caspase-8 activation and cell death
• DISC formation and caspase-10 recruitment depend on caspase-8 scaffold function
• Caspase-10 rewires DISC signaling to NF-κB-induced gene induction/cell survival
• The catalytic activity of caspase-10 and caspase-8 are redundant in gene induction

## Graphical Abstract

**Figure:**
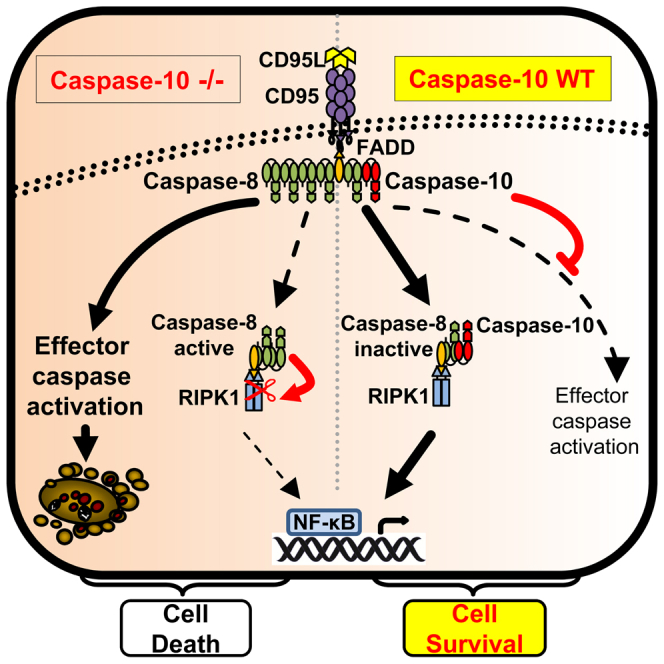

## Teaser

It has been assumed that caspase-10, and its homolog caspase-8, have redundant functions in cell death signaling. Horn et al. now reveal a role for caspase-10 in switching CD95 signaling from caspase-8-induced cell death to NF-κB activation/cell survival. DISC recruitment of caspase-10 and NF-κB activation critically depend upon caspase-8 scaffold function.

## Introduction

The apoptotic signaling cascade can be initiated by extrinsic or intrinsic stimuli. Extracellular death ligands, such as the cluster of differentiation 95 ligand (CD95L) (also known as FasL/Apo-1L) or TRAIL, bind to their respective receptors, most likely to preformed receptor trimers (Chan et al., 2000). Upon ligand binding, the adaptor protein FADD is recruited via its death domain to the receptor. The initiator caspase-8 then binds via its two death effector domains (DED) to the DED of FADD (Sprick et al., 2000). Proteins recruited upon receptor activation form a membrane-bound so-called death-inducing signaling complex (DISC) (Kischkel et al., 1995). After formation of the DISC, the large and small catalytic subunits of the caspase-8 homodimer are cleaved and activate downstream effector caspases (Hughes et al., 2009, Oberst et al., 2010). Additionally, active caspase-8 cleaves substrates, such as Bid, thereby connecting the extrinsic and intrinsic apoptotic pathway (Li et al., 1998). DISC-mediated caspase-8 cleavage is regulated by cFLIP, a DED containing caspase-like protein without protease activity (Irmler et al., 1997). Like caspase-8, cFLIP binds via its DEDs to the DISC and was shown to block caspase-8-mediated cell death (Leverkus et al., 2000, Siegmund et al., 2002, Wachter et al., 2004), although the long isoform of cFLIP (cFLIP_L_) can also activate caspase-8 by mechanisms that are only now becoming clear (Boatright et al., 2004, Dohrman et al., 2005b, Hughes et al., 2016, Micheau et al., 2002, Yu et al., 2009).

The DISC is a complex synergy of recruited proteins, and recent studies have shown that DISC stoichiometry is different than prior models suggested, such that a single FADD molecule is able to recruit a multitude of caspase-8 molecules to the DISC via DED chain assembly (Dickens et al., 2012, Schleich et al., 2012). Death ligand-induced DISC formation is known to activate other signaling cascades beyond caspase-8. When caspase activity is blocked, death ligands can trigger necroptotic cell death, depending on the kinase activity of RIPK1 and RIPK3 (Feoktistova et al., 2011, Feoktistova et al., 2012, Geserick et al., 2009, Tenev et al., 2011). In addition, DISC formation leads to NF-κB activation and cytokine gene induction (Choi et al., 2001, Farley et al., 2008, Park et al., 2003, Schmidt et al., 2015). Death ligand-mediated cytokine production occurs, in part, analogous to tumor necrosis factor (TNF) signaling; RIPK1 is required for the activation of NF-κB by the degradation of IκBα (Peter et al., 2007). Of note, an interaction of RIPK1 with the DISC or soluble caspase-8 after receptor stimulation can primarily be detected whenever caspase activity is blocked (Cullen et al., 2013, Harper et al., 2001). However, it remains to be elucidated how RIPK1 is activated upon DISC formation. Interestingly, cFLIP is known to inhibit DISC-mediated gene induction, indicative of a critical but complex role of DISC-associated caspase-8 and cFLIP for regulation of cell death/gene induction (Kavuri et al., 2011, Wachter et al., 2004).

Caspase-10, a close homolog of caspase-8, is a highly conserved caspase throughout evolution, although absent in rodents (Eckhart et al., 2008, Sakamaki et al., 2015). It is recruited to and processed in the DISC (Kischkel et al., 2001, Sprick et al., 2002, Wang et al., 2001), and both caspase-8 and -10 share overlapping substrate specificities (Fischer et al., 2006). It is currently assumed that caspase-8 and caspase-10 have redundant functions in cell death signaling, but the ability of caspase-10 to substitute for caspase-8 has remained controversial. Due to the lack of caspase-10 in rodents, its function cannot be easily studied in vivo. Published experimental approaches to study its gene function have been mainly limited to overexpression studies that putatively derail the stoichiometry of DISC signaling (Mühlethaler-Mottet et al., 2011).

Here, we have identified an unanticipated role for caspase-10 in switching the CD95L-mediated response from caspase-8-induced cell death to activation of NF-κB and cell survival. We found that caspase-10 impedes caspase-8 activation within the CD95 DISC and that this occurs independently of cFLIP. Moreover, we demonstrate that caspase-10 promotes DISC-mediated gene induction and, independent of its catalytic activity, facilitates NF-κB signaling. Remarkably, we discover an indispensable scaffold function for caspase-8 in DISC formation. Thus, we show that, independent of its enzymatic activity, caspase-8 must bind to FADD to allow further recruitment of caspase-10 and/or cFLIP. Taken together, our data reveal that caspase-10 is a negative regulator of caspase-8-mediated cell death and instead supports CD95-induced gene induction.

## Results

### Caspase-10 Inhibits CD95L-Induced Cell Death

**Figure 1:**
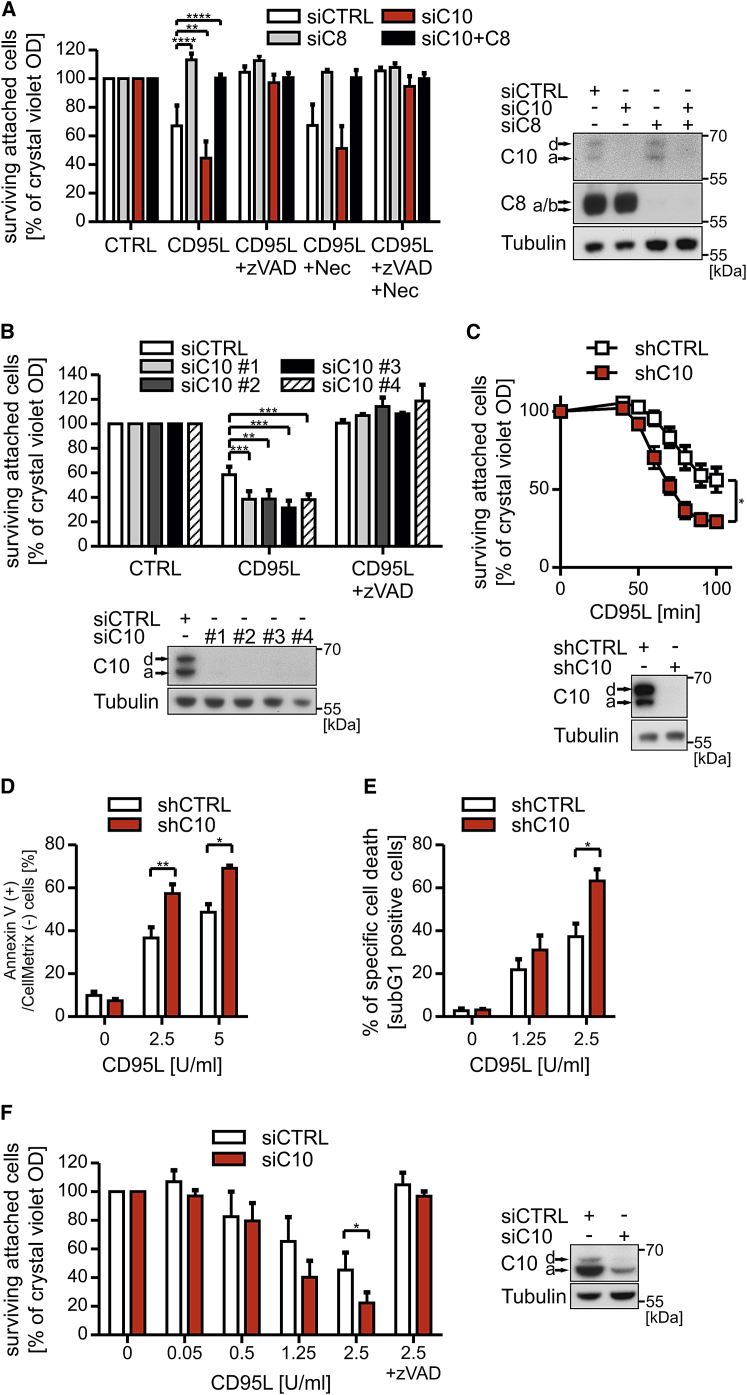
Caspase-10 Impairs CD95L-Induced Cell Death (A) HeLa cells were transfected with caspase-10 (siC10), caspase-8 (siC8), the combination of both, or control siRNA (siCTRL). After 72 hr, triplicates were preincubated with 10 μM zVAD-fmk (zVAD) or 50 μM Necrostatin-1 (Nec) or the combination of both for 1 hr followed by stimulation of 1 U/mL CD95L-Fc for 16–20 hr. Cell viability was analyzed by crystal violet (CV) staining. Knockdown efficiency was controlled by western blotting (WB). (B) HeLa cells were transfected with four different caspase-10 (siC10) or control (siCTRL) siRNAs. After 72 hr, corresponding triplicates were preincubated with 10 μM zVAD for 1 hr followed by stimulation of 1 U/mL CD95L-Fc for 16–20 hr. Cell viability was analyzed by CV staining. Knockdown efficiency was controlled by WB. (C–E) HeLa cells inducibly expressing a shRNA against caspase-10 (shC10) or control shRNA (shCTRL) were treated with 0.5 μg/mL doxycycline for 72 hr. (C) Cells were stimulated in triplicates for the indicated time points with 2 U/mL CD95L-Fc. Cell survival was analyzed as described above. Knockdown efficiency was controlled by WB. (D) Cells were stimulated with the indicated concentrations of CD95L-Fc for 3 hr. Apoptotic cells (annexin V positive and CellMetrix negative) were measured by analyzing the externalization of phosphatidylserine and plasma membrane integrity by Pacific Blue Annexin V plus CellMetrix Green Live/Dead Stain using flow cytometry. A representative experiment is shown in Figure S1A. (E) Cells were stimulated with the indicated CD95L-Fc concentrations for 7 hr. DNA degradation was quantified by flow cytometry using propidium iodide (PI) staining for sub G1 populations. A representative experiment is shown in Figure S1B. (F) SK-Mel cells were transfected with caspase-10 (siC10) and control siRNA (siCTRL). Seventy-two hours later, corresponding triplicates were pre-treated with 10 μM zVAD for 1 hr followed by stimulation with the indicated concentrations of CD95L-Fc for 4 hr. Cell viability was analyzed by CV staining. Knockdown efficiency was controlled by WB. Each graph/diagram represents mean values ± SEM of at least three independent experiments. Significance levels (p values) were measured by Student’s t test (C–F) or two-way ANOVA test (A and B; ^∗^p < 0.05; ^∗∗^p < 0.01; ^∗∗∗^p < 0.001; ^∗∗∗∗^p < 0.0001).

To analyze the function of caspase-10 in CD95L-induced cell death, we performed small interfering RNA (siRNA)-mediated knockdown of caspase-10 in HeLa cells (Figure 1A). Whereas depletion of caspase-8 protected cells from CD95L-induced death, knockdown of caspase-10 reproducibly enhanced CD95-induced cell death. Under these conditions, cell death was caspase-8 mediated, as combined knockdown of caspase-8 and -10 fully protected cells from death induction. Knockdown of caspase-10 using four different siRNAs confirmed the inhibitory function of caspase-10 (Figure 1B). Moreover, HeLa cells expressing a doxycycline-induced caspase-10 short hairpin RNA (shRNA) were also significantly more sensitive to CD95L killing following knockdown of caspase-10 as measured by different assays (Figures 1C–1E and S1). The heightened sensitivity to CD95L was not due to an altered surface expression pattern of CD95 by knockdown of caspase-10 (data not shown). Next, we investigated other cell lines (diverse melanoma lines [SK-Mel, IGR, WK, and MC], B cell and T cell lymphoma [BJAB and Jurkat], and spontaneously transformed keratinocytes [HaCaT]) for the impact of caspase-10 on CD95L-induced cell death. As observed in HeLa cells, SK-Mel melanoma showed a significant sensitivity to CD95L after the depletion of caspase-10 (Figure 1F). In summary, we found that caspase-10 protects from CD95L-induced cell death in three out of eight cell lines examined.

### Caspase-10 and cFLIP Independently Inhibit Caspase-8-Mediated Cell Death

**Figure 2:**
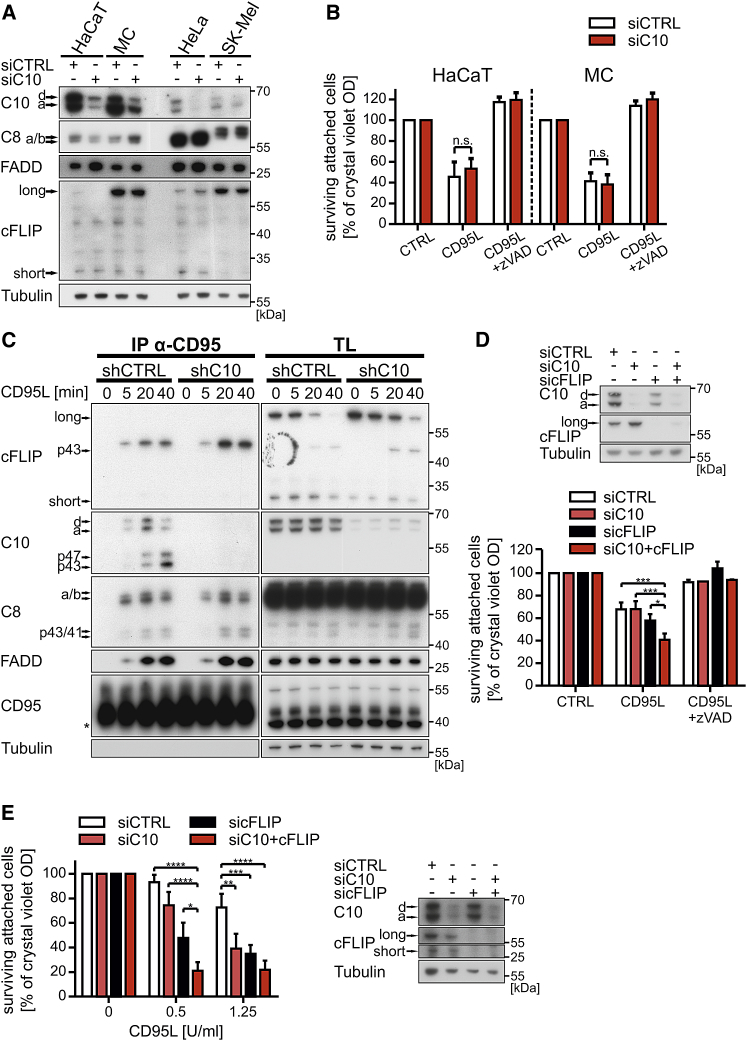
Both cFLIP and Caspase-10 Block Caspase-8-Mediated Cell Death (A) Different cell lines were transfected with siC10 or siCTRL. After 72 hr, knockdown efficiency and proteins involved in DISC signaling were analyzed by WB. Cell lines with high expression of caspase-10 (HaCaT and MC) were compared to low-expressing cell lines (HeLa and SK-Mel). (B) HaCaT and MC cells were treated with siC10 and siCTRL as described in (A). Triplicates were pre-treated with 10 μM zVAD-fmk (zVAD) for 1 hr followed by stimulation of 0.1 (HaCaT) and 2.5 (MC) U/mL CD95L-Fc for 16–20 hr (HaCaT) or 4 hr (MC). Cell viability was analyzed by CV staining. (C) shC10 or shCTRL expression was induced in HaCaT cells by the addition of 0.5 μg/mL doxycycline (Doxy) for 72 hr. Cells were stimulated with 1 U/mL CD95L-Fc for the indicated time points. CD95 was immunoprecipitated from cell lysates (TL), and co-precipitated proteins were analyzed by WB. The asterisk marks a non-specific band. (D) HaCaT cells were transfected with siC10, sicFLIP, the combination of both, or siCTRL. After 48 hr, cells were pre-treated in triplicates with 10 μM zVAD for 1 hr and further stimulated for 4 hr with 0.5 U/mL CD95L-Fc. Cell viability was assayed using CV staining. Knockdown efficiency was controlled by WB. (E) HeLa cells were treated with siRNA as described in (D), stimulated with the indicated concentrations of CD95L-Fc, and analyzed for their cell viability by CV staining. Knockdown efficiency was controlled by WB. Each diagram represents mean values ± SEM of three independent experiments. Significance levels (p values) were measured by Student’s t test (B) or two-way ANOVA test (D and E; ^∗^p < 0.05; ^∗∗^p < 0.01; ^∗∗∗^p < 0.001; ^∗∗∗∗^p < 0.0001; n.s., not significant).

Interestingly, all cell lines unaffected by the knockdown of caspase-10 showed a higher expression level of caspase-10 (examples shown for HaCaT/MC versus HeLa/SK-Mel in Figures 2A and 2B). Remarkably, protein levels after successful knockdown of caspase-10 in HaCaT or MC cells were comparable to endogenous levels present in HeLa or SK-Mel (Figure 2A). This differential stoichiometry of caspase-10 was also reflected in the DISC (Figure S2). We initially hypothesized that the low levels of caspase-10 remaining in these “high expressors” was sufficient to inhibit CD95L-induced cell death. However, a closer look at the expression levels of caspase-10 and cFLIP in HaCaT cells showed a reciprocal counter-regulation of cFLIP after inducible knockdown of caspase-10 (Figures 2C and 2D). Moreover, changes in cFLIP expression were also reflected in the DISC with an increased level of cleaved cFLIP p43 bound to the receptor (Figure 2C). In contrast, caspase-8 or FADD levels in the DISC were unchanged (Figure 2C). These data revealed a close relationship between cFLIP and caspase-10 for inhibition of cell death. To corroborate this assumption, we next combined the knockdown of caspase-10 and cFLIP in HaCaT cells. As shown in Figure 2D, combination of caspase-10 and cFLIP knockdown increased the sensitivity of cells to CD95L (Figures 2D and S3A, dark red columns). As cFLIP expression in HaCaT is very low (Figure 2A), we aimed to extend the data to cell lines with higher cFLIP levels. We thus performed siRNA-mediated knockdown of caspase-10 and cFLIP in HeLa cells. Depletion of either caspase-10 (Figure 2E, light red columns) or cFLIP (Figure 2E, black columns) led to a dramatic increase in CD95-induced cell death. Notably, similar to our data in HaCaT cells, combined knockdown of caspase-10 and cFLIP further increased sensitization to CD95L when compared to caspase-10/cFLIP knockdown alone (Figures 2E and S3B, dark red columns). Taken together, our data showed that caspase-10 is a negative regulator of CD95L-induced cell death, independent of cFLIP. However, at least in HaCaT cells, loss of caspase-10 is compensated by increased expression of cFLIP.

### Caspase-10 Impedes DISC-Mediated Caspase-8 Activation

**Figure 3:**
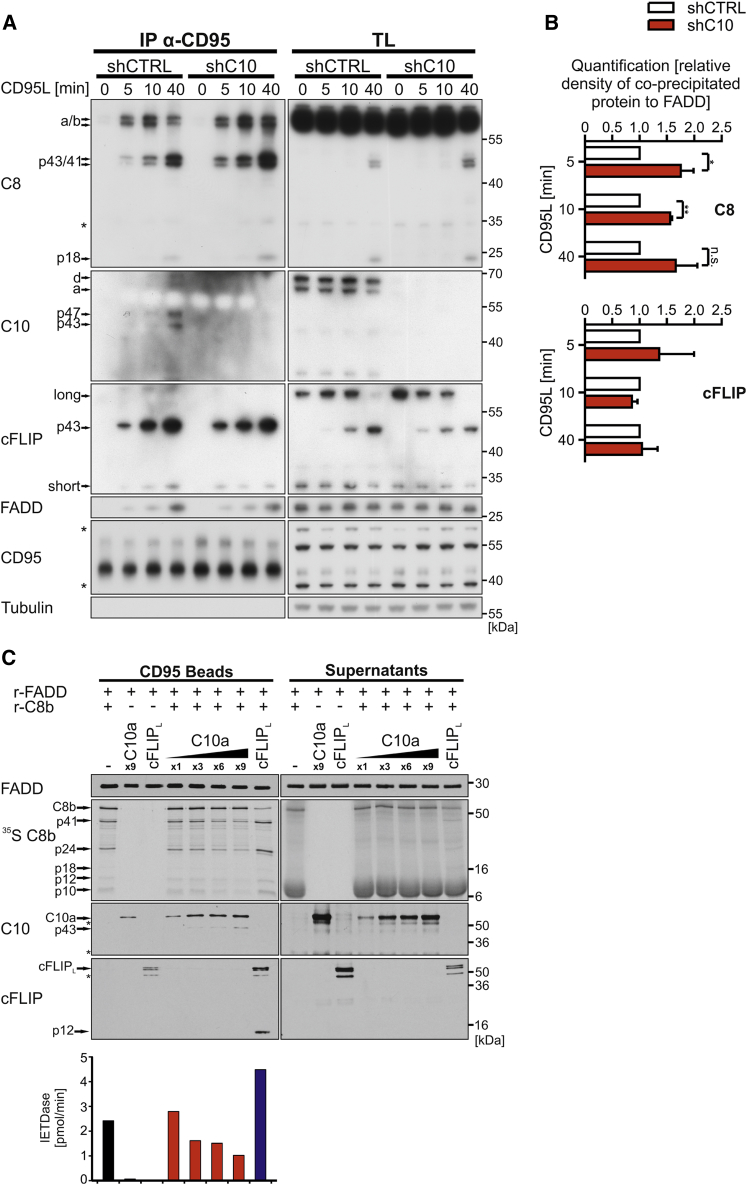
Caspase-10 Impairs Recruitment and Activation of Caspase-8 in the DISC (A) shCTRL or shC10 expression was induced by 0.5 μg/mL doxycycline in HeLa cells. After 72 hr, cells were incubated for the indicated time points with 2 U/mL CD95L-Fc. CD95 was immunoprecipitated from cell lysates (TL), and DISC-associated proteins were analyzed for DISC recruitment by western blotting. The asterisks mark non-specific bands. (B) Relative density of co-precipitated caspase-8 (full-length a/b and p43/41) and cFLIP p43 from the CD95L kinetic shown in (A) was quantified in respect to co-precipitated FADD and normalized to shCTRL cells. Shown are mean values ± SEM of three independent experiments. Significance levels (p values) were measured by Student’s t test (^∗^p < 0.05; ^∗∗^p < 0.01). (C) A functional CD95 DISC was assembled using GST-CD95 intracellular domain (10 μg), recombinant FADD (r-FADD) (5 μg), and ^35^S-labeled recombinant procaspase-8b (r-C8b) (85 μL) at 20°C for 16 hr in the absence or presence of recombinant cFLIP_L_ or increasing amounts of recombinant procaspase-10a (C10a) (25–225 μL; indicated as 1×, 3×, 6×, and 9×). Beads and supernatants were analyzed by SDS-PAGE and autoradiography for ^35^S C8b and immunoblotted for FADD, caspase-10, and cFLIP. Beads were additionally assayed for caspase-8 activity (IETDase). Control CD95 DISCs contained C10a or cFLIP_L_ in the absence of r-C8b. Data shown are representative of three independent experiments. Asterisks mark non-specific bands.

Next, we examined whether caspase-10 modulates assembly of the DISC by characterizing DISC formation in the presence or absence of caspase-10. Upon caspase-10 depletion in HeLa cells, we observed an enrichment of full-length and p43/41 caspase-8 cleavage fragments in the DISC (Figure 3A). Quantification of the ratio of caspase-8 (full length; p43/41) relative to FADD within the DISC confirmed an enhancement of DISC-associated caspase-8 in the absence of caspase-10 (Figure 3B). In contrast, the recruitment of cFLIP to the DISC was unaffected (Figure 3B). Taken together, these findings support our conclusion of a negative regulatory function for caspase-10 in death signaling.

To analyze the proposed inhibitory function of caspase-10 in more detail, we reconstituted the CD95 DISC in a cell-free system using recombinant protein as previously described (Hughes et al., 2009). Strikingly, we observed a concentration-dependent inhibition of caspase-8 by caspase-10 (Figure 3C). Caspase-10 blocked processing of caspase-8 in the DISC and reduced DISC-associated IETDase activity (Figure 3C). Furthermore, our data confirm the ability of cFLIP_L_ to activate caspase-8, as well as the co-operative and hierarchical binding model of caspase-8 and cFLIP (Hughes et al., 2016). As shown in Figure 3C, recruitment of cFLIP_L_ to the complex is enhanced by the presence of caspase-8. Intriguingly, our data suggest a similar model of co-operative binding can now be linked to caspase-10. In the absence of caspase-8, recruitment of caspase-10 to the DISC is inefficient and, importantly, IETDase activity is absent (Figure 3C). Taken together, our data demonstrate that caspase-10 impedes caspase-8 processing, thereby reducing DISC-associated caspase activity.

### Caspase-8 Is Indispensable for the Assembly of the CD95 DISC

**Figure 4:**
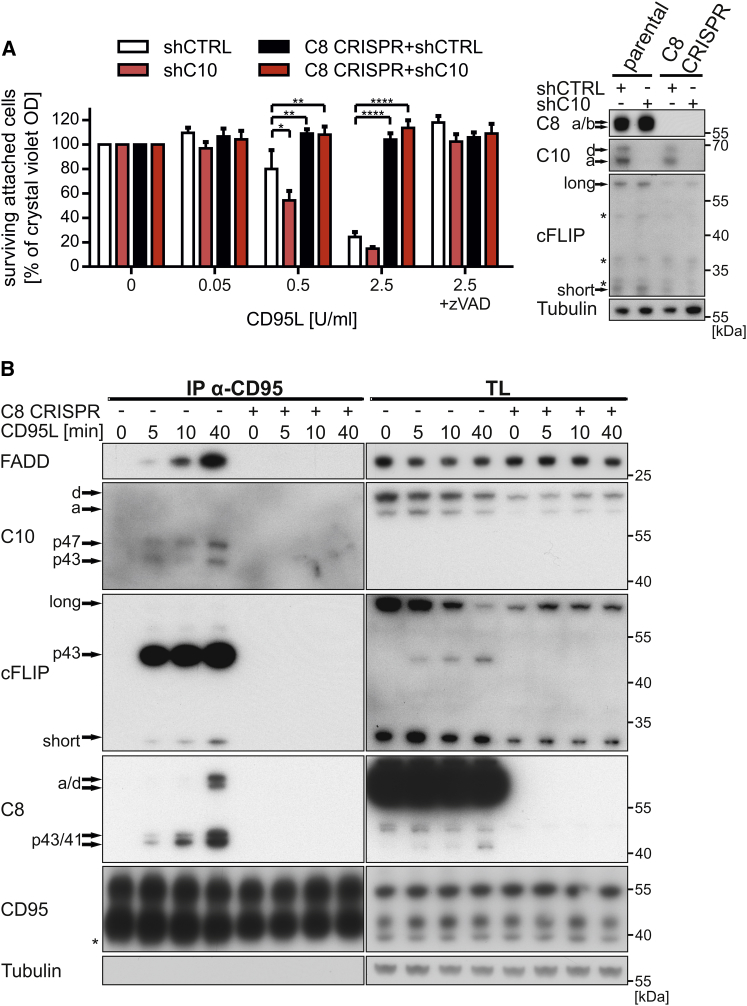
CD95 DISC Formation Requires Caspase-8 (A) Parental and caspase-8-deficient (C8 CRISPR) HeLa cells were treated for 72 hr with 0.5 μg/mL doxycycline to induce the expression of either control or caspase-10 shRNA. Cells were pre-treated for 1 hr with 10 μM zVAD-fmk (zVAD) followed by stimulation with the indicated concentrations of CD95L-Fc for 4 hr. Knockdown efficiency of caspase-10 as well as the knockout of caspase-8 was controlled by WB. Cell viability was analyzed in triplicates by crystal violet staining. Shown are mean values ± SEM of three independent experiments. Significance levels (p values) were measured by two-way ANOVA test (^∗^p < 0.05; ^∗∗^p < 0.01; ^∗∗∗∗^p < 0.0001). (B) CD95 DISC formation was analyzed in parental and HeLa C8 CRISPR cells. CD95 was immunoprecipitated from cell lysates (TL) after stimulation with 2 U/mL CD95L-Fc for the indicated time points. DISC-associated proteins were analyzed by WB. Asterisks mark non-specific bands.

**Figure 5:**
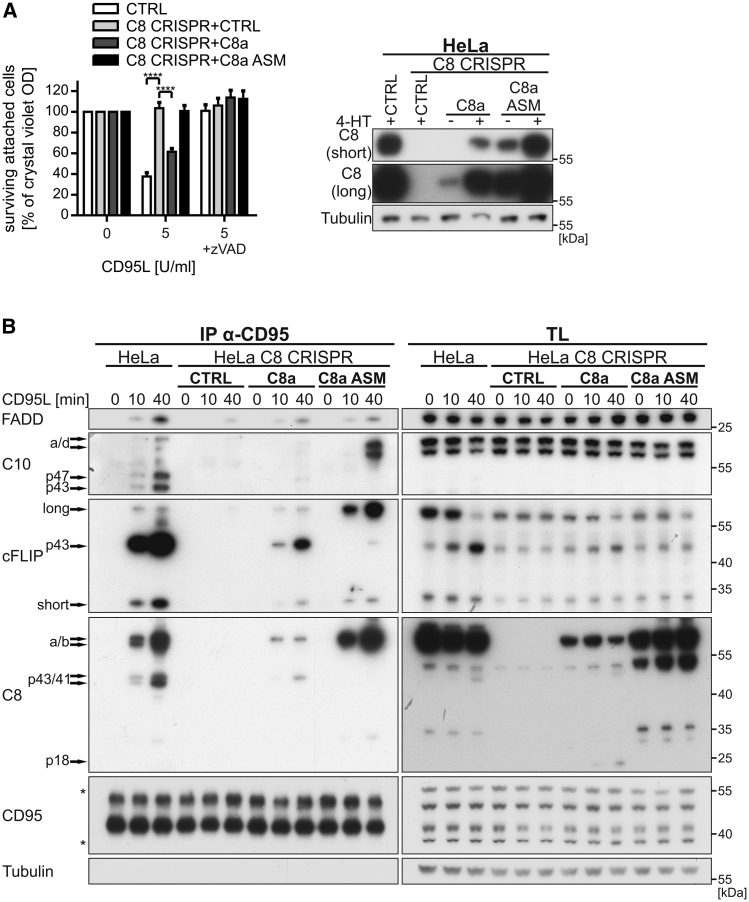
Caspase-8 Scaffold Function Is Indispensable for DISC Recruitment of DED Proteins Caspase-8a (C8a) and its respective active site mutant (C8a ASM) were reconstituted in caspase-8-deficient HeLa (C8 CRISPR) cells. (A) Parental and HeLa C8 CRISPR cells either overexpressing the empty vector (CTRL), C8a, or C8a ASM were treated with 10 nM 4-hydroxytamoxifen (4-HT) for 6 hr to induce the expression of the respective constructs. Cells were pre-treated with 10 μM zVAD-fmk (zVAD) for 1 hr followed by stimulation with 5 U/mL CD95L-Fc for 3 hr. Cell viability was analyzed in triplicates by crystal violet staining. Shown are mean values ± SEM of three independent experiments. Significance levels (p values) were measured by two-way ANOVA test (^∗∗∗∗^p < 0.0001). Expression of C8a and C8a ASM were analyzed by western blotting. (B) As described in (A), C8a was reconstituted in HeLa C8 CRISPR cells for 6 hr by 4-HT. Cell lines were stimulated with 2 U/mL CD95L-Fc for the indicated time points. CD95 was immunoprecipitated from cell lysates (TL) and analyzed for DISC-associated proteins. Asterisks mark non-specific bands.

To further study the co-operative binding of caspase-10 and caspase-8 to the CD95 DISC and to explore whether caspase-10 can substitute for caspase-8 in the native DISC, we next generated caspase-8 knockout HeLa cells using CRISPR/Cas9-mediated recombination. Two independent C8 CRISPR cell clones (C8 CRISPR), generated with two different guide RNAs (gRNAs) (Figure S4), were fully protected from CD95L-induced death irrespective of the expression of caspase-10 (Figure 4A). Intriguingly, C8 CRISPR cells also had repressed cFLIP/caspase-10 levels as compared to parental cells (Figures 4A and 4B, right panels). We next studied DISC composition in the absence of caspase-8. In line with our reconstituted DISC model, we observed that, despite effective precipitation of CD95, the native DISC isolated from C8 CRISPR cells completely lacked cFLIP and caspase-10 (Figure 4B), and strikingly, only weak levels of FADD were detected upon prolonged exposure of the blots. To exclude clonal artifacts during generation of C8 CRISPR cells, we next reconstituted caspase-8a and its respective active site mutant (ASM) in C8 CRISPR cells by inducible overexpression. When these cells were analyzed for CD95L-induced cell death, re-expression of caspase-8, but not its ASM, conferred sensitivity to CD95L-induced cell death, despite a lower overall expression level of the enzymatically active caspase-8a (Figure 5A). Intriguingly, reconstitution of caspase-8 protein restored the recruitment of cFLIP, caspase-10, and, importantly, FADD in the DISC independent of the enzymatic activity of caspase-8 (Figure 5B). The caspase-8 ASM, however, fully protected cleavage of caspase-8, caspase-10, or cFLIP, indicative of the critical importance of caspase-8 enzymatic activity for induction of cell death, but not initial recruitment of DED proteins. Thus, our data reveal a central role for caspase-8 in formation of the CD95 DISC and places caspase-8 upstream of both cFLIP and caspase-10 in DISC signaling.

### Caspase-10 Promotes CD95L-Mediated NF-κB Activation and Gene Induction

**Figure 6:**
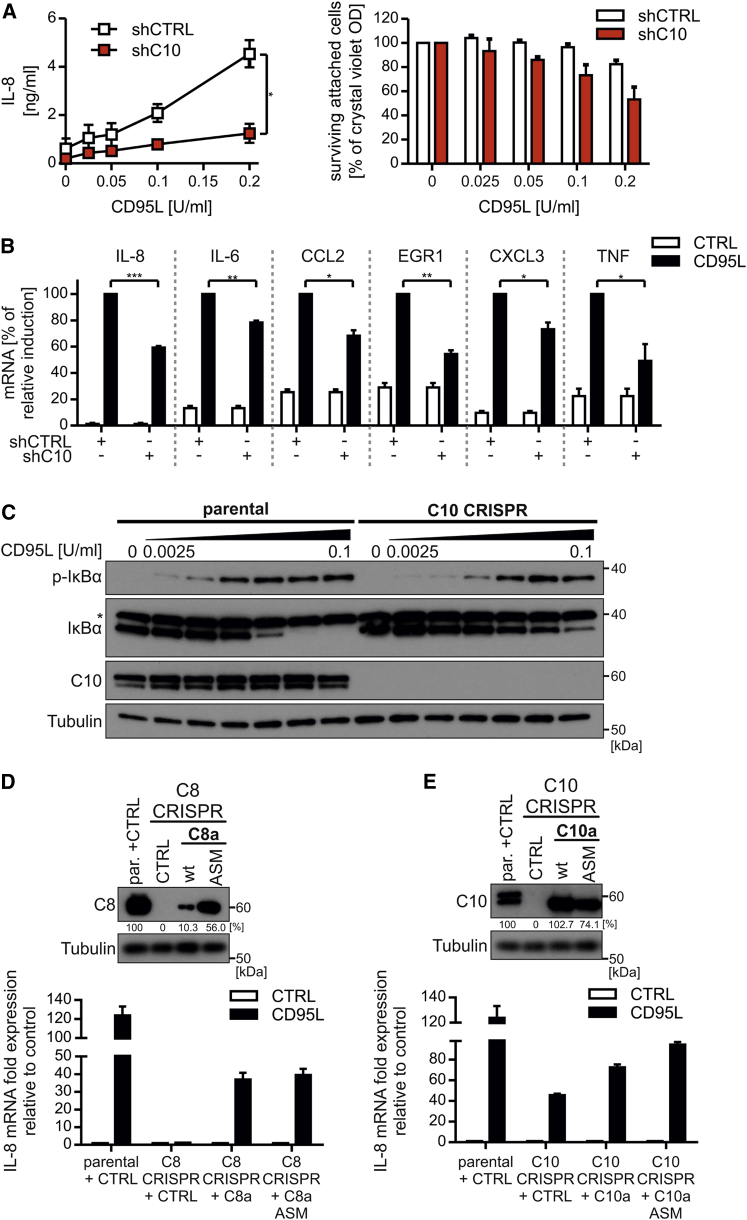
Caspase-10 Promotes CD95L-Mediated Gene Induction (A and B) HeLa cells expressing shC10 or shCTRL were treated for 72 hr with 0.5 μg/mL doxycycline. (A) Duplicate wells were stimulated in media containing 0.5% FCS with the indicated concentrations of CD95L-Fc for 24 hr. Supernatants were analyzed for secreted interleukin-8 (IL-8) by ELISA. Cell viability was assayed using crystal violet staining. (B) HeLa ± shC10 cells were pre-starved for 4 hr in media containing 0.5% FCS followed by treatment with 10 μM zVAD-fmk (zVAD) for 1 hr. Cells were stimulated with 0.1 U/mL CD95L-Fc for 3 hr. RNA was isolated, reverse transcribed to cDNA, and mRNA expression levels of *IL-8*, *IL-6*, *CCL2*, *EGR1*, *CXCL3*, and *TNF* were analyzed by real-time qPCR. (C) Parental and caspase-10-deficient (C10 CRISPR) HeLa cells were starved and pre-treated with zVAD as described in (B). Cells were stimulated with CD95L-Fc (0.0025, 0.005, 0.01, 0.025, 0.05, or 0.1 U/mL) for 3 hr. IκBα phosphorylation as well as degradation and caspase-10 knockout were analyzed by western blotting. Asterisks mark non-specific bands. (D) Parental and caspase-8-deficient (C8 CRISPR) HeLa cells were treated with 10 nM 4-HT for 6 hr in media containing 0.5% FCS to induce the expression of either control plasmid or caspase-8a (expression of caspase-8a ASM was achieved in the absence of induction via 4-HT). Cells were stimulated with zVAD and CD95L-Fc as described in (B) and analyzed for *IL-8* mRNA expression by real-time qPCR. Caspase-8 expression was quantified after reconstitution and compared to parental HeLa cells as indicated in the western blots. Relative *IL-8* mRNA induction has been calculated with respect to caspase-8 expression. (E) Parental, C10 CRISPR, and reconstituted caspase-10a/ASM HeLa cells were treated as described in (D) (expression of wild-type caspase-10 was achieved in the absence of induction via 4-HT). Cells were stimulated with zVAD and CD95L-Fc as described in (B) and *IL-8* mRNA expression analyzed. Relative caspase-10 expression and *IL-8* induction has been calculated as described in (D) (raw data for D and E are shown in Figures S6C and S6D). Each graph/diagram represents mean values ± SEM of three independent experiments. Significance levels (p values) were measured by Student's t test (^∗^p < 0.05; ^∗∗^p < 0.01; ^∗∗∗^p < 0.001).

We thus far explored caspase-10 for its impact on DISC-mediated cell death signaling. However, the function of the enzymatic activity of caspase-10 remains unclear. We reasoned that another function of caspase-10 might be related to the known gene-inductive properties exerted by CD95 stimulation (Cullen et al., 2013, Wallach et al., 1999). Moreover, it is well known that zVAD strongly supports DISC-mediated gene induction (Harper et al., 2001), and we have also previously reported that QVD allows for TRAIL-induced gene activation (Kavuri et al., 2011). However, when we compared zVAD and QVD for their impact on CD95L-mediated *interleukin-8* (*IL-8*) mRNA expression, *IL-8* induction was unaffected by QVD (Figure S5A). Furthermore, we observed that QVD was, firstly, inefficient in blocking CD95L-induced cell death compared to zVAD (Figure S5B) and, secondly, only partially blocked processing of caspase-8 after DISC stimulation (Figure S5C). Therefore, we characterized the role of caspase-10 in death-receptor-mediated gene induction in HeLa cells by microarray analysis in the presence of zVAD to achieve maximal gene expression. We observed that caspase-10 knockdown did not affect the subset of genes induced upon CD95L stimulation; rather it impacts on the amplitude of induction of a variety of NF-κB-induced target genes (Table S1). Of note, we identified a number of CD95L-induced genes to be deregulated by knockdown of caspase-10 (Table S1, light orange), with three genes exhibiting >25% repression of gene induction (Table S1, dark orange). Thus, we aimed to verify selected genes in more detail and importantly demonstrated that loss of caspase-10 significantly repressed IL-8 secretion after CD95L stimulation (Figure 6A). Furthermore, we analyzed the impact of caspase-10 on six CD95L-induced genes via real-time qPCR and observed that caspase-10 knockdown significantly reduced CD95L-mediated gene induction by 20%–50% in all targets examined (Figure 6B). As described for TNF-R-signaling, CD95L-induced gene induction is driven by multiple protein kinases, including the IKK complex, JNK, or p38 mitogen-activated protein (MAP) kinases (Cullen et al., 2013, Wallach et al., 1999). To study the impact of caspase-10 on these kinases, we generated caspase-10 knockout (C10 CRISPR) HeLa cells, which confirmed the heightened sensitivity to CD95L stimulation observed by knockdown approaches (Figure S6A). Whereas we failed to detect obvious differences in the phosphorylation status of JNK or p38 MAP kinase (MAPK) under conditions with or without caspase-10 expression (data not shown), CD95L-mediated IκBα degradation/phosphorylation was inhibited in C10 CRISPR cells (Figures 6C and S6B).

Corroborating our findings of an upstream role for caspase-8 in DISC formation, CD95L-induced cytokine gene induction was fully absent in C8 CRISPR cells. In contrast, reconstitution with caspase-8a/ASM allowed for *IL-8* mRNA induction, albeit to a lesser extent than parental cells (Figure 6D). To accommodate for differing expression levels between re-expressed wild-type and ASM caspase-8, we examined *IL-8* mRNA induction relative to caspase-8 expression in parental cells (Figures 6D and S6C). Moreover, C10 CRISPR cells confirmed our previous data obtained by inducible knockdown. In the absence of caspase-10, *IL-8* mRNA induction was reduced following CD95L stimulation (Figure 6E), whereas reconstitution with wild-type or ASM caspase-10a increased *IL-8* induction in these cells (Figures 6E and S6D). Our experiments using reconstituted caspase-8a ASM, as well as caspase-10a ASM, clearly showed that caspase-mediated cytokine induction occurs independently of catalytic activity (Figures 6D and 6E). Thus, caspase-10 and 8 promote DISC-mediated gene induction, revealing an intricate balance of gene-inducing/death-promoting abilities of these two DISC-associated tandem DED proteins.

## Discussion

To date, studies about DISC signaling have focused on the function of caspase-8 and its regulator cFLIP. In contrast, the role of caspase-10 is more controversial and less understood. The interplay of the tandem DED proteins caspase-8 and -10 and cFLIP is complex; in our study, downregulation of one of these proteins frequently resulted in a rapid counter-regulation of at least one of the other tandem DED proteins. For example, loss of caspase-8 leads to a downregulation of cFLIP (Figures 4 and 5), HaCaT cells counter-regulate the loss of caspase-10 by upregulation of cFLIP (Figure 2), and caspase-8-deficient Jurkat cells were shown to downregulate caspase-10 (Sprick et al., 2002). These phenomena occurred remarkably quickly during cell culture, thus showing how closely these proteins are inter-linked and critical for cell survival. Notably, caspase-10/8 and cFLIP are located on the same genetic locus (2q33-q34), and co-regulated genes tend to be clustered in the same genetic neighborhood (Michalak, 2008). Taken together, our findings underscore the necessity to simultaneously study all tandem DED proteins.

We focused our attention on the role of caspase-10 and strikingly found that this caspase is a negative regulator of DISC-mediated apoptosis (Figure 1). Thus far, caspase-10 was reported to be a pro-apoptotic initiator caspase, similar to caspase-8 (Engels et al., 2005, Fischer et al., 2006). Importantly, our data exclude a pro-apoptotic function of caspase-10, independent of the cell line or the experimental setting used (siRNA-/shRNA-mediated knockdown or gene knockout). Significantly, our reconstituted DISC approach clearly demonstrates the negative impact of caspase-10 on caspase-8 in a cell-free system (Figure 3). In line with our data and a more complex role of caspase-10, a recent study has proposed a pro-survival function of endogenous caspase-10, as it was shown to inhibit autophagic cell death in multiple myeloma cell lines (Lamy et al., 2013).

**Figure 7:**
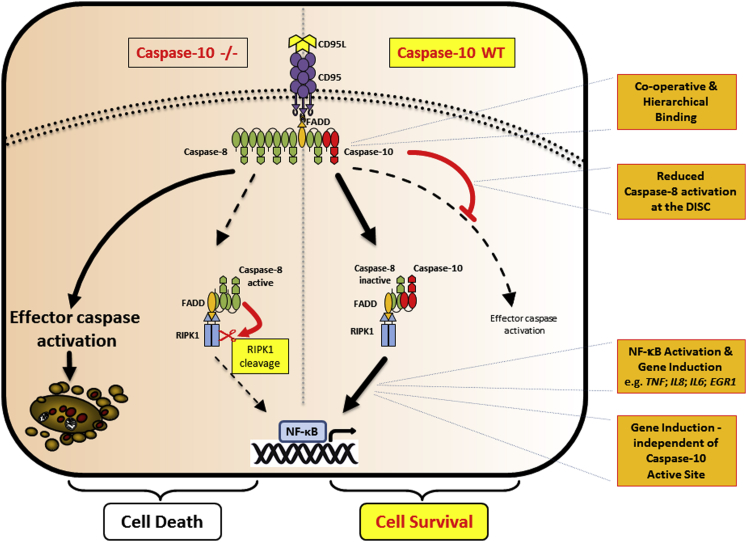
A Dual Role for Caspase-10 in DISC Signaling In this model, FADD binding to the CD95 receptor initiates formation of the DISC. Caspase-8 binding is then required to stabilize the FADD receptor association and enable subsequent recruitment of caspase-10. The presence or absence of caspase-10 defines the amplitude of the cell death response. In the absence of caspase-10, caspase-8 DED chain assembly strongly activates downstream effector caspases to induce apoptosis. Simultaneously, caspase-8 promotes RIPK1 activation by mechanisms that to date have not been elucidated. Next, NF-κB dimerizes and localizes to the nucleus to induce cytokine gene expression. In the presence of caspase-10, DISC-mediated caspase-8 activity and subsequent cell death are reduced. Moreover, caspase-10, together with caspase-8, favors RIPK1 activation and NF-κB-mediated gene induction, independent of caspase-10/caspase-8 catalytic activity. Thus, taken together, caspase-10 switches the cell death response in favor of cell survival and cytokine gene expression.

When we studied the impact of caspase-10 mechanistically, caspase-10 was shown to impair recruitment/processing of caspase-8 in the DISC (Figure 3). Previous mass spectrometry analysis of the native CD95- and TRAIL DISC demonstrated that FADD is clearly sub-stoichiometric as compared to caspase-8, leading to a paradigm-changing model of DED chain elongation that is mainly driven by caspase-8 (Dickens et al., 2012, Schleich et al., 2012). In marked contrast, the ratio of caspase-8 to caspase-10 or cFLIP protein levels, respectively, are much lower and until recently have not been examined in detail. A recent report has suggested that short DED proteins regulate caspase-8 activation in DED chains (Schleich et al., 2016). Moreover, we have recently shown that overexpression of cFLIP_S_ disrupts caspase-8 chain assembly (Hughes et al., 2016). Based on the data we present here, we hypothesize that caspase-10 may also disrupt caspase-8 chain elongation (Figure 7), but this will require further investigation. In line with this assumption, overall levels of DISC-bound caspase-8 were reduced in the presence of caspase-10 (Figure 3). Furthermore, IETDase activity of the reconstituted DISC was repressed by caspase-10 (Figure 3). Interestingly, DISC-associated caspase-10 was unable to activate IETDase in the absence of caspase-8 (Figure 3), despite reports that in vitro dimerized caspase-10 catalytic subunits harbor IETDase activity (Wachmann et al., 2010). However, this finding suggests another possibility for caspase-10 in terms of inhibiting cell death. Caspase-10 and caspase-8 could potentially form heterodimers with reduced or absent activity. In this regard, heterodimerization between caspase-8/10 and cFLIP_L_ has been reported (Boatright et al., 2004, Yu et al., 2009), but it is highly controversial whether the resulting heterodimer has pro- or anti-apoptotic functions (reviewed in van Raam and Salvesen, 2012).

In line with our very recent report on cFLIP (Hughes et al., 2016), our studies demonstrate that caspase-8 is located upstream of both caspase-10 and cFLIP in the cell death pathway with critical relevance for DISC formation (Figures 4 and 5). Our re-expression studies with wild-type/ASM caspase-8 in C8 CRISPR cells restored recruitment of caspase-10, cFLIP, and importantly FADD to the DISC, independent of the catalytic activity of caspase-8 (Figure 5). Moreover, we show that FADD association within the DISC is stabilized by caspase-8 binding (Figures 4 and 5). Thus, our data demonstrate that a scaffold function of caspase-8 is both necessary and critical for DISC formation. However, in contrast to its homolog caspase-8, caspase-10 is not essential for DISC signaling. Altogether, our studies reveal that caspase-10 and cFLIP negatively regulate cell death signaling within the DISC but that this occurs downstream of caspase-8 recruitment. This finding is in contrast to previous reports made in caspase-8-deficient Jurkat cell lines, in which caspase-10 was recruited to the DISC in the absence of caspase-8 (Sprick et al., 2002). Importantly, we observed a weakly expressed truncated form of caspase-8 in caspase-8-deficient Jurkat cells (data not shown). Thus, because low levels of caspase-8 can restore caspase-10 and cFLIP recruitment to the DISC (Figure 5), these findings likely explain the previous results with caspase-8-deficient Jurkat cells (Sprick et al., 2002). Therefore, some of the collected knowledge about apoptotic and gene-inductive signaling gained from caspase-8-deficient Jurkat cells has to be critically re-evaluated.

In addition to the apoptosis-inducing machinery, DISC-mediated gene induction is an important signaling pathway that is activated concomitant to apoptotic cell death (Peter et al., 2007) but is most frequently observed under conditions independent of apoptotic caspase-8 activation, e.g., in the presence of broad spectrum caspase inhibitors (Harper et al., 2001, Kavuri et al., 2011, Leverkus et al., 2003). Importantly, we observed that QVD failed to support DISC-mediated gene induction and that it is inefficient in blocking CD95L-induced caspase-8 cleavage and cell death (Figure S5). Very recently, it has been shown that QVD, in contrast to zVAD, is a weak inhibitor of the caspase-8/cFLIP_L_ heterodimer, whereas both almost equally block the caspase-8 homodimer (Brumatti et al., 2016). Studies on the function of cFLIP_L_ in the activation of NF-κB upon DISC formation are contradictory. It has been reported that the cFLIP p43 fragment promotes the recruitment of TRAF2 and RIPK1 to the caspase-8/cFLIP heterodimer, resulting in more efficient activation of NF-κB (Dohrman et al., 2005a). In contrast, several studies reported an inhibitory function of cFLIP_L_ in DISC-mediated gene induction (Kavuri et al., 2011, Kreuz et al., 2004). This clearly needs to be further examined but puts the caspase-8/cFLIP heterodimer in the spotlight for DISC-induced NF-κB activation. Strikingly, our data now show that caspase-10 promotes DISC-mediated cytokine expression by enhancing IκBα degradation/phosphorylation (Figure 6; Table S1). Whereas the molecular mechanisms of DISC-mediated gene induction are largely unknown, our studies show that the scaffold function of caspase-8 is essential for DISC formation as well as NF-κB activation (Figure 6); moreover, the catalytic activity of caspase-8 and -10 is redundant for NF-κB activation and subsequent cytokine induction. However, it still remains to be elucidated how and where caspase-8 and -10 activate RIPK1 to initiate the phosphorylation of IκBα.

Our data for CD95L demonstrate that caspase-10 shifts the apoptotic cell death response following DISC formation to the activation of NF-κB and cell survival (Figure 7), both beneficial features for tumor cells. Thus, caspase-10 may have tumorigenic properties. Chronic inflammatory gene expression favors tumor formation and progression (Chai et al., 2015), and CD95 signaling has been shown in several studies to favor tumor growth and invasiveness (Barnhart et al., 2004, Chen et al., 2010). Thus, our data hint at the possibility of modulating caspase-10 expression as a therapeutic oncological target; repression of caspase-10 reduces cytokine expression (Figure 6) and favors apoptosis (Figure 1), potentially resulting in impaired tumor growth. Whether DISC-mediated gene induction is responsible for CD95 dependency of cancer cells remains unanswered. However, we have previously demonstrated that primary keratinocytes activate NF-κB upon stimulation of the TRAIL DISC, and they are 5-fold less sensitive to death-ligand-induced apoptosis than transformed keratinocytes (Kavuri et al., 2011, Leverkus et al., 2000). Thus, gene induction is potentially much more relevant in DISC signaling than currently assumed.

Here, we reveal the elusive, and in part controversial, role of caspase-10 in DISC signaling. Our discovery of caspase-10 as a negative regulator of cell death and a facilitator of gene induction separates the function of cFLIP and caspase-10 in the DISC. Crucially, we demonstrate that caspase-8 binding via FADD to the receptor is an indispensable initiating step in DISC formation and NF-κB activation. Moreover, our data clearly delineate that caspase-10 is not an initiator of DISC-induced cell death signaling as previously believed. Instead, caspase-10 and caspase-8 scaffold function promote DISC-mediated gene induction, revealing an intricate balance of gene-inducing and death-protecting abilities of the different DISC-associated tandem DED proteins (Figure 7). These findings change our current understanding of extrinsic apoptotic signaling and open new possibilities in terms of signaling via other caspase-8 and -10 and cFLIP-containing complexes, such as the ripoptosome or TNF complex (Feoktistova et al., 2011, Micheau and Tschopp, 2003, Tenev et al., 2011).

## Experimental Procedures

Supplemental Experimental Procedures contain details of materials, antibodies, siRNA transfection, cytotoxicity assays, western blot analysis, IL-8 ELISA, and primer sequences used for real-time qPCR.

### Generation of Stable Cell Lines

To generate stable cell lines that inducibly overexpress caspase-8a/10a and their respective active site mutants by addition of 4-hydroxytamoxifen, cells were transduced with a pF GEV16 Super PGKHygro as previously described (Diessenbacher et al., 2008). Viral vectors inducibly expressing a shRNA against caspase-10 (V3THS_394573) or the respective control shRNA (TRIPZ inducible lentiviral non-silencing shRNA control) via the pTRIPZ lentiviral shRNAmir system by addition of doxycycline were purchased from GE Healthcare and used for production of lentiviral particles using a second-generation packing system. In brief, HEK293T cells were transfected with 3 μg pMD2.G, 7.5 μg pSPax2, and 3 μg pcDNA3.1/p35 of lentiviral packaging vectors together with the transfer vector pF 5 × UAS W SV40 Puro, which expresses caspase-8 in a Gal4-dependent fashion. Caspase-8a/ASM was subcloned from pcDNA3.1 (Hughes et al., 2009) and caspase-10a/ASM from pEF6/V5-His-TOPO into lentiviral vector system using standard cloning procedures and verified by sequencing. Supernatants were harvested 24 and 48 hr post-transfection and filtered (0.45 μm filter; GE Healthcare). Viral particles were added to the cells with 5 μg/mL polybrene, and cells were spin infected for 1.5 hr at 30°C. Antibiotic-resistant stable cell lines were selected in 300 μg/mL hygromycin and/or 1 μg/mL puromycin for 3–7 days.

Caspase-8/10-deficient HeLa cells were generated using the CRISPR/Cas9 system. HeLa cells were transiently transfected using Lipofectamine LTX Reagent with PLUS Reagent (Thermo Fisher Scientific) according to manufacturer's recommendations. HeLa cells were cotransfected with the pMA-T vector (carrier of the cassette-U6-gRNA(casp8)-TTTTT; Life Technologies) and hCas9-pcDNA3.3-TOPO (Addgene) to generate caspase-8 knockout cells. Caspase-10 knockout cells were generated by the use of the pSpCas9(BB)-2A-GFP (PX458) plasmid (Addgene). gRNA insertion was performed as previously described (Ran et al., 2013). gRNA sequences were designed using the open access software provided at http://crispr.mit.edu/ to target the 5′ end of the gene and thus all isoforms of either caspase-8 or 10. The gRNA sequences were as follows:

Casp8-1: GCCTGGACTACATTCCGCAA
Casp8-2: GCTCTTCCGAATTAATAGAC
Casp10: GGGGGTCCAAGATGTGGAGA

Two days post-transfection, cells were sorted with a BD FACSAria I (BD Biosciences) and single clones isolated and analyzed for successful caspase-8/10 knockout.

### Immunoprecipitation of the CD95 Receptor

For the precipitation of DISC-associated proteins, 1.5 × 10^7^ cells were used. The procedure of CD95 precipitation was performed as described previously (Cullen et al., 2013). Equal amounts of precipitates were analyzed by western blotting as described above.

### Reconstitution of the CD95 DISC

Complete reconstitutions were carried out essentially as described previously (Hughes et al., 2009). Recombinant procaspase-10a was produced by in vitro transcription/translation (TNT T7-coupled reticulocyte lysate system; Promega) using pEF6/V5-His-TOPO vector containing caspase-10a or its respective ASM.

### Real-Time qPCR

RNA isolation, cDNA synthesis, primer design, and real-time qPCR as well as data analysis were performed as described previously (Feoktistova et al., 2011). Data shown have been normalized to *GAPDH* and have been confirmed by normalization to *18S*.

### Microarray Analysis

HeLa cells were seeded in 6-well plates, and respective shRNA expression was induced by the addition of 0.5 μg/mL doxycycline for 72 hr. In three independent experiments, cells were prestarved for 4 hr with media containing 0.5% fetal calf serum (FCS) followed by zVAD-fmk treatment (10 μM) for 1 hr. Cells were then stimulated with 0.1 U/mL CD95L for 3 hr. Total RNA from stimulated or control cells was isolated and tested by capillary electrophoresis on an Agilent 2100 bioanalyzer (Agilent Technologies) to confirm RNA quality. Gene expression profiling was performed using arrays of human Hugene-2_0-st-type from Affymetrix according to manufacturer's instructions. Bioinformatic evaluations were done as previously described (Czymai et al., 2010). Significant regulated genes (adjusted p values [false discovery rate (FDR)] < 0.05) were considered by a fold change >2 compared to unstimulated control cells.

### Statistics

Statistical analysis was carried out using GraphPad Prism. Statistical significance (p values) was analyzed using either paired Student’s t test (two-tailed) or two-way ANOVA, including Dunnett corrections for multiple comparisons, as indicated in the figure legends.

## Author Contributions

S.H. designed and carried out most of the experiments. R.S. and M.P. performed essential additional experiments. C.S. performed and supervised microarray experiments and analyzed the resulting data. T.T. and P.M. initiated generation of CRISPR/Cas9 knockout cell lines and advised the respective experiments. M.A.H. and M.M. advised and performed the reconstituted DISC analysis. M.M., P.M., and M.R.S. discussed data throughout the study and advised experiments. M.L. conceptualized and initiated the study, obtained funding, designed experiments, and oversaw the study. S.H. and M.L. wrote the paper, with input from M.M., P.M., M.R.S., and M.A.H., particularly during the revision process. All authors reviewed the manuscript.

## Accession Numbers

The accession number for the microarray dataset reported in this paper is GEO: GSE75365.

## Supplemental Information

Document S1. Supplemental Experimental Procedures, Figures S1–S6, and Table S1

Document S2. Article plus Supplemental Information
