# Supplementary material for: Caspase-10 Negatively Regulates Caspase-8-Mediated Cell Death, Switching the Response to CD95L in Favor of NF-κB Activation and Cell Survival
Source: Cell Rep. 2017 Apr 25;19(4):785–97. doi: 10.1016/j.celrep.2017.04.010 (PMC5413585; doi:10.1016/j.celrep.2017.04.010)
Supplement: Document S1. Supplemental Experimental Procedures, Figures S1–S6, and Table S1 [file mmc1.pdf]

Cell Reports, Volume *19*

Supplemental Information

Caspase-10 Negatively Regulates Caspase-8-Mediated Cell Death, Switching the Response to CD95L in Favor of NF-κB Activation and Cell Survival

Sebastian Horn, Michelle A. Hughes, Ramon Schilling, Carsten Sticht, Tencho Tenev, Michaela Ploesser, Pascal Meier, Martin R. Sprick, Marion MacFarlane, and Martin Leverkus

Supplemental Information

Caspase-10 is a negative regulator of caspase-8-mediated cell death, switching the response to CD95L in favour of NF-κB activation and cell survival

Sebastian Horn[1*], Michelle A Hughes[2], Ramon Schilling[1], Carsten Sticht[3], Tencho Tenev[4], Michaela Ploesser[1], Pascal Meier[4], Martin R. Sprick[5*], Marion MacFarlane[2*‡], Martin Leverkus[1,6†]

Inventory of Supplemental Information

Supplemental Figures S1-6 and Table SI:

Figure S1, related to Figure 1
Caspase-10 blocks CD95L-induced cell death

Figure S2, related to Figure 2
Cellular expression levels reflect the stoichiometry of caspase-10 in the DISC

Figure S3, related to Figure 2
Loss of caspase-10 and/or cFLIP promotes CD95L-induced cell death

Figure S4, related to Figure 4
Caspase-8 knockout prevents CD95L-induced cell death

Figure S5, related to Figure 6
QVD does not support CD95L-mediated gene induction and is a weak inhibitor of caspase-8-mediated cell death

Figure S6, related to Figure 6
Caspase-10 and -8 induce DISC-mediated gene expression

Table SI, related to Figure 6
Analysis of CD95L-mediated gene induction by microarray after knockdown of caspase-10

Supplemental Experimental Procedures

Supplemental References

Supplemental Figures

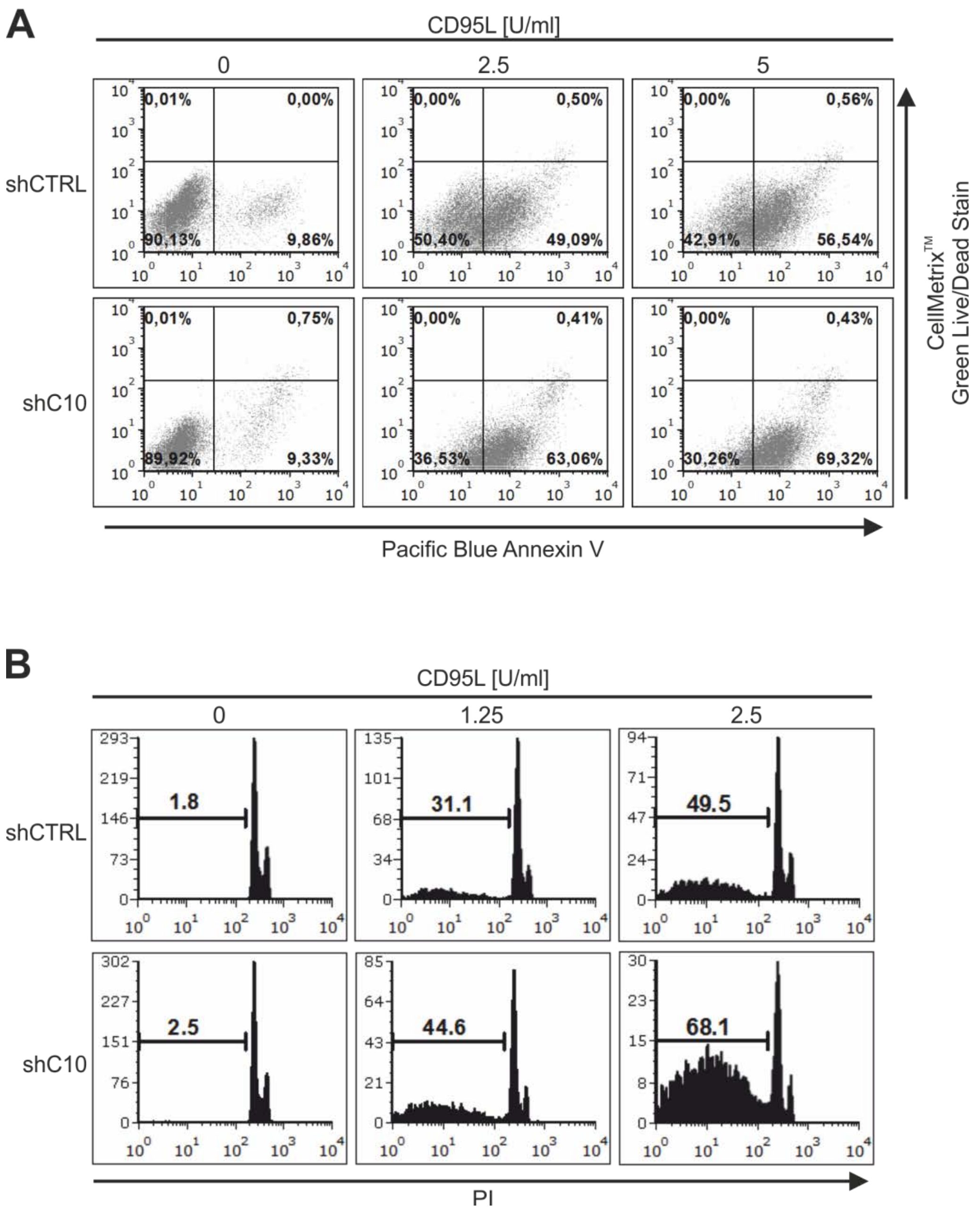

Figure S1, related to Figure 1. Caspase-10 blocks CD95L-induced cell death

(A) Cells were stimulated with the indicated concentrations of CD95L-Fc for 3 h. Apoptotic cells (Annexin V positive and CellMetrix negative) were measured by analyzing the externalization of phosphatidylserine and plasma membrane integrity by Pacific Blue Annexin V plus CellMetrix Green Live/Dead Stain using flow cytometry. A representative experiment is shown. (**B)** Cells were stimulated with the indicated CD95L-Fc concentrations for 7 h. DNA degradation was quantified by flow cytometry using PI staining for sub G1 populations. A representative experiment is shown.

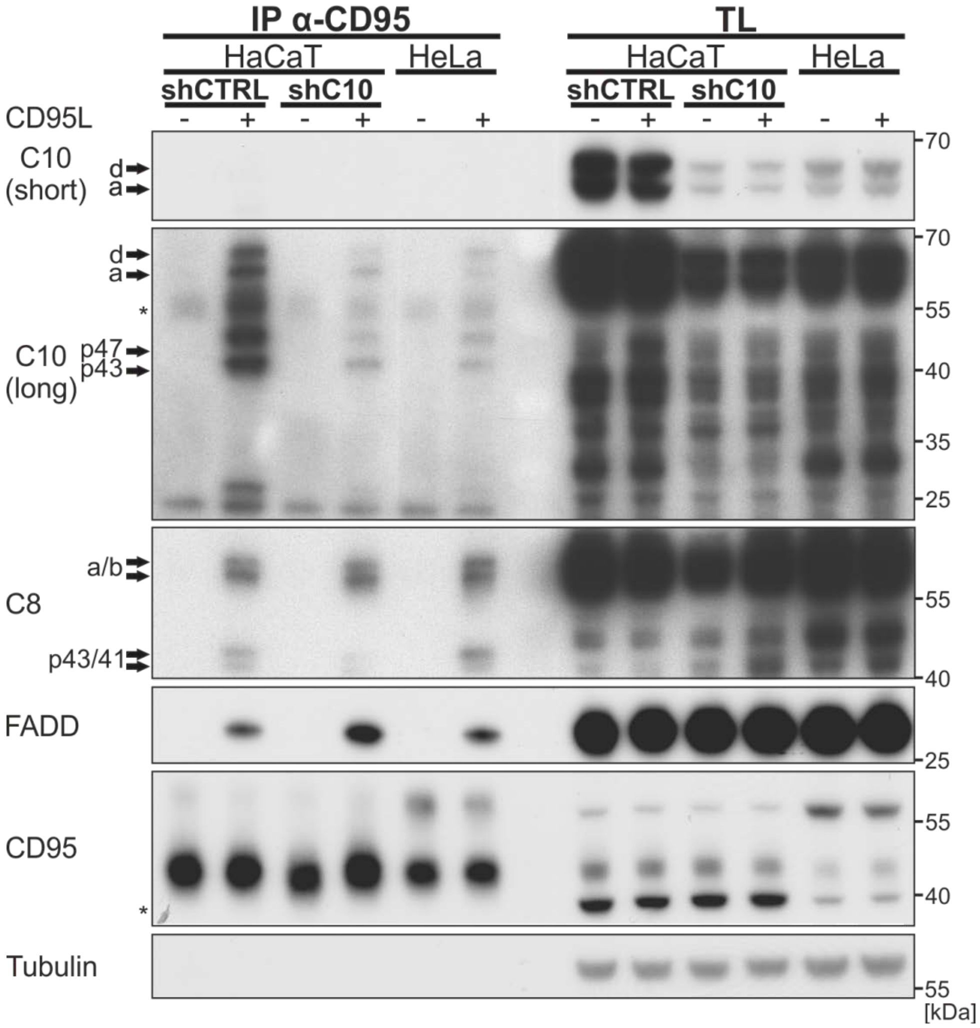

Figure S2, related to Figure 2. Cellular expression levels reflect the stoichiometry of caspase-10 in the DISC

Caspase-10 (shC10) or control (shCTRL) shRNA expression was induced in HaCaT cells by the addition of 0.5 µg/ml doxycycline for 72 h. Cells were stimulated with 1 U/ml CD95L-Fc for 40 min and compared to equivalently-treated HeLa cells. CD95 was immunoprecipitated from cell lysates (TL) and co-precipitated proteins were analyzed by Western blotting. The asterisks mark non-specific bands.

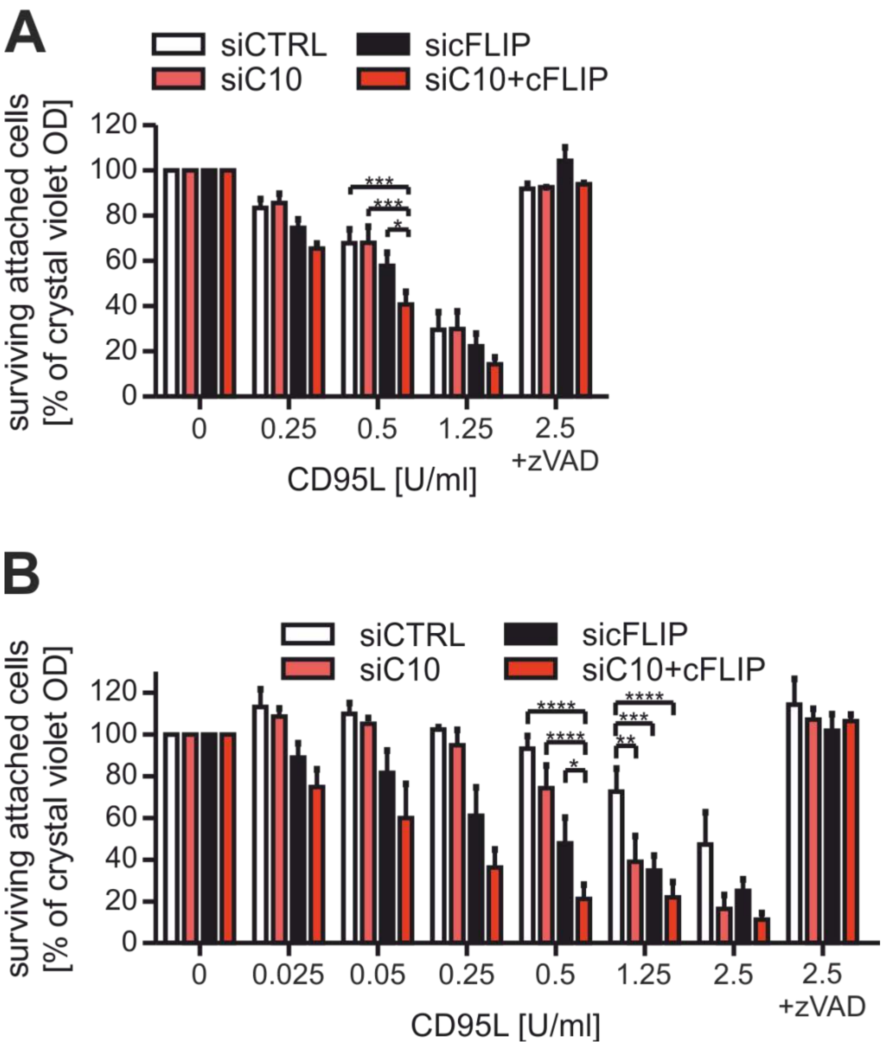

Figure S3, related to Figure 2. Loss of caspase-10 and/or cFLIP promotes CD95L-induced cell death

Dose-response to CD95L-Fc stimulation in HaCaT (**A**) and HeLa (**B**) cells after cFLIP and/or caspase-10 knockdown by siRNA. Cells were transfected with siC10, sicFLIP, the combination of both, or siCTRL. After 48 h, cells were pre-treated in triplicates with 10 µM zVAD for 1 h and further stimulated for 4 h with the indicated concentrations of CD95L-Fc, and cell viability analyzed by crystal violet staining. Each value represents mean ± SEM of three independent experiments. Significance levels (p values) were measured by two-way ANOVA test (*p<0.05; **p<0.01; ***p<0.001; ****p<0.0001).

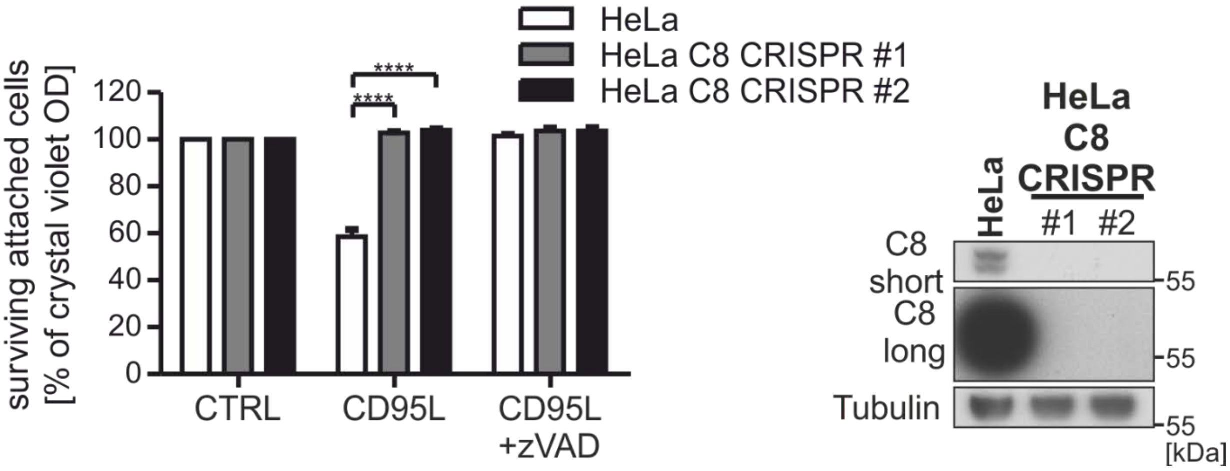

Figure S4, related to Figure 4. Caspase-8 knockout prevents CD95L-induced cell death

Caspase-8 deficient HeLa cell lines were generated using the CRISPR-Cas9 system with two different gRNAs targeting caspase-8. Parental HeLa cells as well as caspase-8 deficient cells (HeLa C8 CRISPR) were pre-treated with 10 µM zVAD-fmk (zVAD) for 1 h followed by stimulation with 1 U/ml CD95L-Fc for 16-20 h. Cell viability was analyzed in triplicates by crystal violet staining. Shown are mean values ± SEM of three independent experiments. Significance levels (p values) were measured by two-way ANOVA test (****p<0.0001). Knockout efficiency was measured by Western blotting.

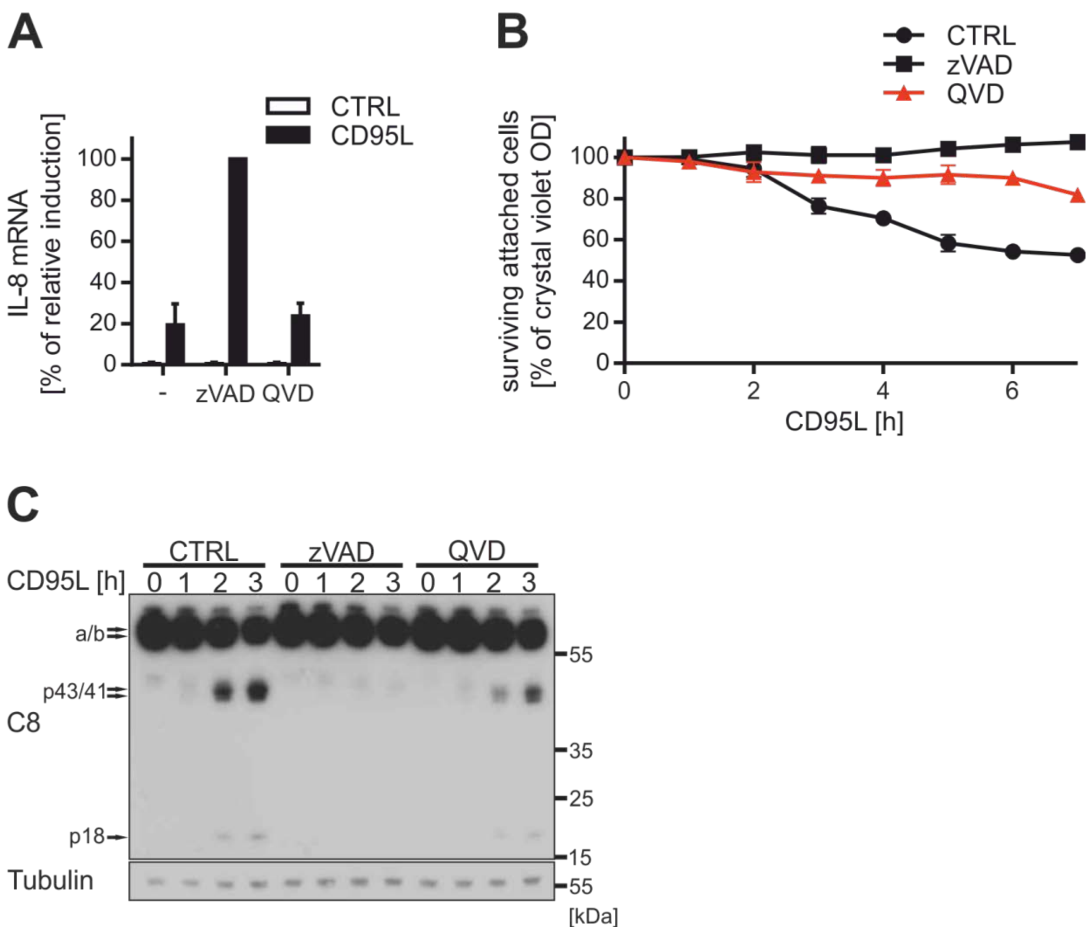

Figure S5, related to Figure 6. QVD does not support CD95L-mediated gene induction and is a weak inhibitor of caspase-8-mediated cell death

(**A**) HeLa+shCTRL cells were starved for 4 h in media containing 0.5 % FCS and pre-treated with 10 µM zVAD-fmk or 10 µM QVD-OPh for 1 h. Cells were stimulated with 0.1 U/ml CD95L-Fc for 3 h in the presence or absence of ZVAD-fmk or QVD-OPh. RNA was isolated, reverse transcribed to cDNA and mRNA expression levels of *IL-8* were analyzed by RT qPCR. Values shown are the average ± range of two independent experiments. **(B)** Triplicates of HeLa+shCTRL cells were pre-treated with 10 µM zVAD-fmk or 10 µM QVD-OPh and stimulated for the indicated time points with 0.1 U/ml CD95L-Fc. Cell viability was assayed by crystal violet staining. Shown are mean values of three technical replicates from one experiment. (**C**) HeLa+shCTRL cells were pre-stimulated as described in B and treated for the indicated time points with 0.1 U/ml CD95L-Fc. Total cell lysates were analyzed for caspase-8 processing by Western blotting.

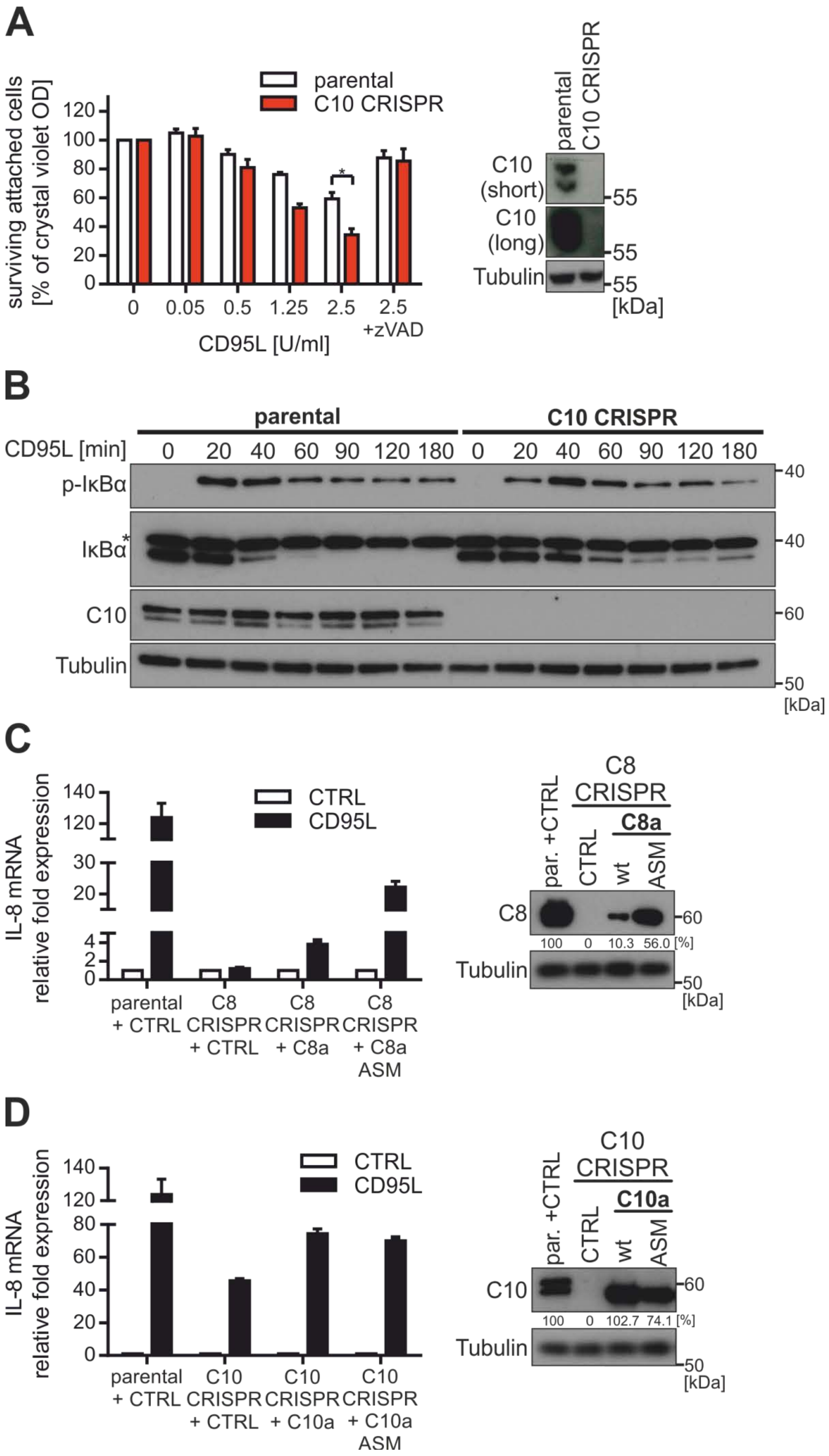

A
surviving attached cells [% of crystal violet OD]
parental
C10 CRISPR
CD95L [U/ml]
+zVAD
C10 (short)
C10 (long)
Tubulin
[kDa]
B
parental
C10 CRISPR
CD95L [min]
p-IκBα
IκBα
C10
Tubulin
[kDa]
C
IL-8 mRNA relative fold expression
CTRL
CD95L
parental + CTRL
C8 CRISPR + CTRL
C8 CRISPR + C8a
C8 CRISPR + C8a ASM
C8 CRISPR
par. +CTRL
CTRL
C8a
wt
ASM
C8
100 0 10.3 56.0 [%]
Tubulin
[kDa]
D
IL-8 mRNA relative fold expression
CTRL
CD95L
parental + CTRL
C10 CRISPR + CTRL
C10 CRISPR + C10a
C10 CRISPR + C10a ASM
C10 CRISPR
par. +CTRL
CTRL
C10a
wt
ASM
C10
100 0 102.7 74.1 [%]
Tubulin
[kDa]

Figure S6, related to Figure 6. Caspase-10 and -8 induce DISC-mediated gene expression

(**A**) Parental and caspase-10 deficient (C10 CRISPR) HeLa cells were pre-treated for 1 h with 10 µM zVAD-fmk (zVAD). Cells were stimulated in triplicate with the indicated concentrations of CD95L-Fc for 4 h. Cell death was analyzed by crystal violet staining. Caspase-10 knockout was controlled by Western blotting. (**B**) Parental and C10 CRISPR HeLa cells were starved for 4 h in media containing 0.5 % FCS, pretreated with 10 µM zVAD, and stimulated with 0.1 U/ml CD95L-Fc for the indicated time points. Caspase-10 knockout and IκBα degradation, as well as phosphorylation, were analyzed by Western blotting. The asterisk marks a non-specific band. (**C**) Raw data for Figure 6D (without the correction for caspase-8 expression levels). (**D**) Raw data for Figure 6E (without the correction for caspase-10 expression levels). Shown are mean values ± SEM of three independent experiments. Significance levels (p values) were measured by Student´s t-test (*$p<0.05$).

Table SI, related to Figure 6: Analysis of CD95L-mediated gene induction by microarray following knockdown of caspase-10

Gene symbol	log2 fold change (CD95L/CTRL)		Fold change (CD95L/CTRL)		p-values (CD95L/CTRL)		Relative repression of gene induction by shC10 [% of fold change]
	shCTRL	shC10	shCTRL	shC10	shCTRL	shC10	
CCL20	4.65	4.75	25.11	26.91	< 0.001	< 0.001	-7.2
IL8	4.57	4.17	23.75	18.00	< 0.001	< 0.001	24.2
PTX3	2.69	2.41	6.45	5.31	< 0.001	< 0.001	17.6
CCL2	2.09	1.80	4.26	3.48	0.015	0.031	18.2
IL6	2.07	1.71	4.20	3.27	< 0.001	0.001	22.1
CXCL3	2.03	1.76	4.08	3.39	0.012	0.022	17.1
EGR1	1.99	1.17	3.97	2.25	< 0.001	< 0.001	43.4
TNFAIP3	1.96	1.56	3.89	2.95	< 0.001	< 0.001	24.2
IL1A	1.85	1.53	3.61	2.89	0.001	0.005	19.9
NFKBIA	1.77	1.46	3.41	2.75	< 0.001	< 0.001	19.3
JUN	1.60	1.12	3.03	2.17	< 0.001	0.001	28.3
EFNA1	1.51	1.59	2.85	3.01	< 0.001	< 0.001	-5.7
IRF1	1.46	1.45	2.75	2.73	< 0.001	< 0.001	0.7
ICAM1	1.36	1.26	2.57	2.39	0.001	0.001	6.7
NFKBIE	1.16	0.79	2.23	1.73	< 0.001	0.002	22.6
TNF	1.16	0.61	2.23	1.53	0.001	0.031	31.7
OLR1	1.12	1.21	2.17	2.31	< 0.001	< 0.001	-6.4
DUSP5	1.08	0.91	2.11	1.88	< 0.001	< 0.001	11.1
NUAK2	1.06	1.05	2.08	2.07	< 0.001	< 0.001	0.7
NR4A2	1.04	0.76	2.06	1.69	< 0.001	0.002	17.6
KDM6B	1.03	0.93	2.04	1.91	0.003	0.007	6.7
SOD2	1.02	1.11	2.03	2.16	< 0.001	< 0.001	-6.4
IER5	1.01	0.69	2.01	1.61	0.002	0.020	19.9
CSF1	1.00	0.99	2.00	1.99	0.005	0.005	0.7

Dark orange: Significant reduction in gene expression (log2 fold change CD95L (shC10/shCTRL) < -0.5)
Light orange: Relative gene repression by shC10 > 15 %

Supplemental Experimental Procedures

Cell Lines

The cell lines, HeLa (cervical carcinoma, kindly provided by Dr. Michael Boutros), HaCaT (spontaneously transformed keratinocytes, generously provided by Dr. Petra Boukamp), SK-Mel and MC (melanoma) were cultured in DMEM high glucose + GlutaMAX^{TM} (Gibco, supplemented with 10 % FCS, 1 % Hepes, and 1 % sodium pyruvate) and maintained at 5 % CO_2 and 37 °C. Cell lines were cultured as previously described (Feoktistova et al., 2011; Geserick et al., 2015, 2009).

Materials

The following antibodies were used for Western blotting: antibodies to caspase-8 (C-15; kindly provided by P.H. Krammer), caspase-10 (4C1; MBL, Nagoya, Japan), cFLIP (NF-6; Alexis, San Diego, CA, USA), FADD (Clone 1; BD Transduction Laboratories™, San Diego, CA, USA); CD95 (C20; Santa Cruz, Delaware Avenue, CA, USA), IκBα (C-21; Santa Cruz, Delaware Avenue, CA, USA), phospho-IκBα (5A5; Cell Signaling Technology, Danvers, MA, USA), and β-tubulin (clone 2.1; Sigma-Aldrich, St.Louis, MO, USA). An expression construct for production of CD95L-Fc (Bossen et al., 2006) was kindly provided by P. Schneider (University of Lausanne, Epalinges, Switzerland). 1 U of CD95L-Fc was determined as 1 U/ml supernatant that was sufficient to kill 50% (LD50) of parental HeLa cells within 16-20 h. Ligand-mediated cell death was fully blocked by addition of soluble CD95-Fc protein. HRP-conjugated goat anti-rabbit, and goat anti–mouse IgG1 and IgG2b were obtained from SouthernBiotech (Birmingham, AL, USA). CD95 antibody for immunoprecipitation (Apo-1 IgG3) was kindly provided by P.H. Krammer (Dhein et al., 1992). Pacific Blue^{TM} Annexin V was from BioLegend (San Diego, CA, USA) and CellMetrix^{TM} Green Live/Dead Stain was purchased from ImmunoChemistry Technologies (Bloomington, MN, USA). 4-hydroytamoxifen, Necrostatin-1, cycloheximide, and propidium iodide were from Sigma-Aldrich (St. Louis, MO, USA). Doxycycline was purchased from Hexal (Holzkirchen, Germany), QVD-OPh from MP Biomedicals (Santa Ana, CA), and zVAD-fmk from Bachem (Bubendorf, Switzerland).

Transient Transfection of siRNA

For transient knockdown experiments the following siRNA duplexes were used: FlexiTube siRNA for caspase-8 (Hs_CASP8_11), caspase-10 (Hs_CASP10_8, Hs_CASP10_9, Hs-_CASP10_10, and Hs_CASP10_11), cFLIP (Hs_CFLAR_9) and the respective control siRNA (AllStars neg. control siRNA 1027281). All siRNA preparations were from QIAGEN (Venlo, Netherlands). For transient transfection $2x10^5$ cells per well were seeded in a 6-well plate and incubated overnight. Prior to transfection cells were incubated with Opti-MEM (Gibco 11058-021) medium for 20 min followed by transfection according to the

manufacturer´s recommendations using Lipofectamine 2000 (Thermo Fischer Scientific, Waltham, MA, USA) and the respective siRNA species. In case of single cFLIP knockdown, the molarity of cFLIP siRNA was reduced to 500 pM (HeLa) and 1 nM (HaCaT).

Cytotoxicity Assays

Crystal violet staining of attached living cells was performed after stimulations with the indicated concentrations and time points of CD95L-Fc in three technical replicates per condition in 96-well plates as previously described (Leverkus et al., 2000). Experiments were performed 3-5 times and the optical density of control conditions (cells treated with diluents) was normalized to 100 % to allow comparison of independent experiments. Subdiploid DNA content was analyzed as previously described (Diessenbacher et al., 2008) and measured by FACSCanto II analysis. To analyze the externalization of phosphatidylserine and plasma membrane integrity, $1x10^5$ HeLa cells were seeded per well in a 6-well plate and stimulated as described in the figure legend and further handled exactly as previously described (Geserick et al., 2009) with the exception that Pacific Blue Annexin V and CellMetrix Green Live/Dead Stain were used.

Western blotting

Cell lysates were prepared as previously described (Diessenbacher et al., 2008) and 10-15 µg of total cellular proteins were separated by SDS-PAGE on 4-12% gradient gels (Life Technologies, Carlsbad, CA) followed by transfer to PVDF membranes. Blocking of membranes and incubation with primary and appropriate secondary antibodies were essentially performed as described previously (Geserick et al., 2009). Bands were visualized with ECL detection kits (Luminata™ Forte Western HRP Substrate, Millipore, Schwalbach, Germany; Pierce ECL Western Blotting Substrate, Waltham, MA, USA). Respective protein bands were quantified by densitometry with *ImageJ* software using non-saturated exposures of blots.

Measurement of interleukin-8

$6x10^4$ HeLa cells were seeded per well in a 24-well plate and incubated over night at 37 °C. Interleukin-8 (IL-8) secretion was measured from cell free cell culture supernatants using the IL-8 ELISA kit obtained from BD Bioscience (Franklin Lakes, NJ, USA), and performed according to the manufacturer´s recommendations. Each assay was repeated at least three times and carried out using duplicate samples from each cell culture supernatant.

RT qPCR (primer sequences)

The following primers were used for the RT qPCR reaction:

IL-8

forward 5’- CAC CCC AAA TTT ATC AAA GA -3’;

reverse 5’- ACT GGC ATC TTC ACT GAT TC -3’;

TNF

forward 5’- TCA GAT CAT CTT CTC GAA CC -3’;

reverse 5’- TGG TTA TCT CTC AGC TCC AC -3´;

IL-6

forward 5’- ACT CAC CTC TTC AGA ACG AAT TG -3’;

reverse 5’- CCA TCT TTG GAA GGT TCA GGT TG -3´;

CCL2

forward 5’- CAG CCA GAT GCA ATC AAT GCC -3’;

reverse 5’- TGG AAT CCT GAA CCC ACT TCT -3´;

EGR1

forward 5’- GGT CAG TGG CCT AGT GAG C -3’;

reverse 5’- GTG CCG CTG AGT AAA TGG GA -3´;

CXCL3

forward 5’- CCA AAC CGA AGT CAT AGC CAC -3’;

reverse 5’- TGC TCC CCT TGT TCA GTA TCT -3´;

GAPDH

forward 5’- CCT GGT ATG ACA ACG AAT TT -3’;

reverse 5’- AGT GAG GGT CTC TCT CTT CC -3’;

18S

forward 5´- GAG GAT GAG GTG GAA CGT GT -3’;

reverse 5´- TCT TCA GTC GCT CCA GGT CT -3’.

Supplemental References

Bossen, C., Ingold, K., Tardivel, A., Bodmer, J.-L., Gaide, O., Hertig, S., Ambrose, C., Tschopp, J., Schneider, P., 2006. Interactions of tumor necrosis factor (TNF) and TNF receptor family members in the mouse and human. J. Biol. Chem. 281, 13964–13971.

Dhein, J., Daniel, P.T., Trauth, B.C., Oehm, A., Möller, P., Krammer, P.H., 1992. Induction of apoptosis by monoclonal antibody anti-APO-1 class switch variants is dependent on cross-linking of APO-1 cell surface antigens. J. Immunol. 149 , 3166–3173.

Diessenbacher, P., Hupe, M., Sprick, M.R., Kerstan, A., Geserick, P., Haas, T.L., Wachter, T., Neumann, M., Walczak, H., Silke, J., Leverkus, M., 2008. NF-[kappa]B Inhibition Reveals Differential Mechanisms of TNF Versus TRAIL-Induced Apoptosis Upstream or at the Level of Caspase-8 Activation Independent of cIAP2. J. Invest. Dermatol. 128, 1134–1147.

Feoktistova, M., Geserick, P., Kellert, B., Dimitrova, D.P., Langlais, C., Hupe, M., Cain, K., MacFarlane, M., Häcker, G., Leverkus, M., 2011. CIAPs Block Ripoptosome Formation, a RIP1/Caspase-8 Containing Intracellular Cell Death Complex Differentially Regulated by cFLIP Isoforms. Mol. Cell 43, 449–463.

Geserick, P., Hupe, M., Moulin, M., Wong, W.W.-L., Feoktistova, M., Kellert, B., Gollnick, H., Silke, J., Leverkus, M., 2009. Cellular IAPs inhibit a cryptic CD95-induced cell death by limiting RIP1 kinase recruitment. J. Cell Biol. 187, 1037–1054.

Geserick, P., Wang, J., Schilling, R., Horn, S., Harris, P.A., Bertin, J., Gough, P.J., Feoktistova, M., Leverkus, M., 2015. Absence of RIPK3 predicts necroptosis resistance in malignant melanoma. Cell Death Dis. 6, e1884.

Leverkus, M., Neumann, M., Mengling, T., Rauch, C.T., Bröcker, E.-B., Krammer, P.H., Walczak, H., 2000. Regulation of Tumor Necrosis Factor-related Apoptosis-inducing Ligand Sensitivity in Primary and Transformed Human Keratinocytes. Cancer Res. 60 , 553–559.
