# Supplementary material for: Caspase-10 Negatively Regulates Caspase-8-Mediated Cell Death, Switching the Response to CD95L in Favor of NF-κB Activation and Cell Survival
Source: Cell Rep. 2017 Apr 25;19(4):785–97. doi: 10.1016/j.celrep.2017.04.010 (PMC5413585; doi:10.1016/j.celrep.2017.04.010)
Supplement: Document S2. Article plus Supplemental Information [file mmc2.pdf]

Cell Reports

Article

Caspase-10 Negatively Regulates Caspase-8-Mediated Cell Death, Switching the Response to CD95L in Favor of NF-κB Activation and Cell Survival

Graphical Abstract

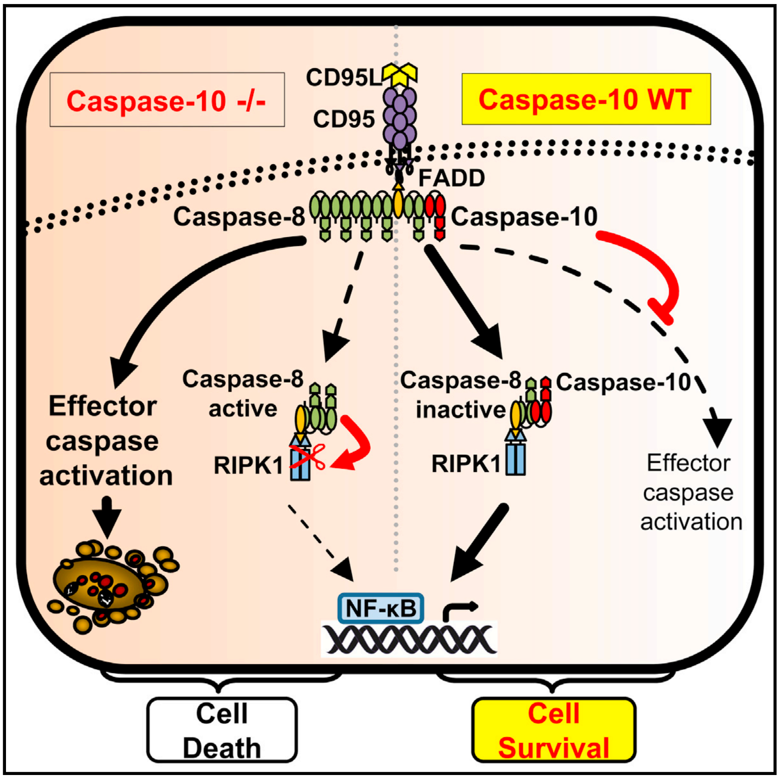

Highlights

- Caspase-10 negatively regulates DISC-mediated caspase-8 activation and cell death
- DISC formation and caspase-10 recruitment depend on caspase-8 scaffold function
- Caspase-10 rewires DISC signaling to NF-κB-induced gene induction/cell survival
- The catalytic activity of caspase-10 and caspase-8 are redundant in gene induction

Authors

Sebastian Horn, Michelle A. Hughes, Ramon Schilling, ..., Martin R. Sprick, Marion MacFarlane, Martin Leverkus

Correspondence

sebastian-horn@gmx.net (S.H.), m.sprick@dkfz-heidelberg.de (M.R.S.), mm21@leicester.ac.uk (M.M.)

In Brief

It has been assumed that caspase-10, and its homolog caspase-8, have redundant functions in cell death signaling. Horn et al. now reveal a role for caspase-10 in switching CD95 signaling from caspase-8-induced cell death to NF-κB activation/cell survival. DISC recruitment of caspase-10 and NF-κB activation critically depend upon caspase-8 scaffold function.

Accession Numbers

GSE75365

Horn et al., 2017, Cell Reports *19*, 785–797
April 25, 2017 © 2017 The Authors.
http://dx.doi.org/10.1016/j.celrep.2017.04.010

Cell Reports
Article

OPEN ACCESS
CellPress

Caspase-10 Negatively Regulates Caspase-8-Mediated Cell Death, Switching the Response to CD95L in Favor of NF-κB Activation and Cell Survival

Sebastian Horn,[1,*] Michelle A. Hughes,[2] Ramon Schilling,[1] Carsten Sticht,[3] Tencho Tenev,[4] Michaela Ploesser,[1] Pascal Meier,[4] Martin R. Sprick,[5,*] Marion MacFarlane,[2,8,*] and Martin Leverkus[1,6,7]
[1]Section of Molecular Dermatology, Department of Dermatology, Venereology, and Allergology, Medical Faculty Mannheim, University of Heidelberg, Theodor-Kutzer-Ufer 1-3, 68167 Mannheim, Germany
[2]MRC Toxicology Unit, Hodgkin Building, PO Box 138, Lancaster Road, Leicester LE1 9HN, UK
[3]Center for Medical Research, Medical Faculty Mannheim, University of Heidelberg, Theodor-Kutzer-Ufer 1-3, 68167 Mannheim, Germany
[4]The Breakthrough Toby Robins Breast Cancer Research Centre, Institute of Cancer Research, Mary-Jean Mitchell Green Building, Chester Beatty Laboratories, Fulham Road, London SW3 6JB, UK
[5]Heidelberg Institute for Stem Cell Technology and Experimental Medicine (HI-STEM gGmbH), Im Neuenheimer Feld 280, 69120 Heidelberg, Germany
[6]Department of Dermatology and Allergology, Medical Faculty of the RWTH Aachen, Pauwelsstraße 30, 52074 Aachen, Germany
[7]Deceased
[8]Lead Contact
*Correspondence: sebastian-horn@gmx.net (S.H.), m.sprick@dkfz-heidelberg.de (M.R.S.), mm21@leicester.ac.uk (M.M.)
http://dx.doi.org/10.1016/j.celrep.2017.04.010

SUMMARY

Formation of the death-inducing signaling complex (DISC) initiates extrinsic apoptosis. Caspase-8 and its regulator cFLIP control death signaling by binding to death-receptor-bound FADD. By elucidating the function of the caspase-8 homolog, caspase-10, we discover that caspase-10 negatively regulates caspase-8-mediated cell death. Significantly, we reveal that caspase-10 reduces DISC association and activation of caspase-8. Furthermore, we extend our cooperative/hierarchical binding model of caspase-8/cFLIP and show that caspase-10 does not compete with caspase-8 for binding to FADD. Utilizing caspase-8-knockout cells, we demonstrate that caspase-8 is required upstream of both cFLIP and caspase-10 and that DISC formation critically depends on the scaffold function of caspase-8. We establish that caspase-10 rewires DISC signaling to NF-κB activation/cell survival and demonstrate that the catalytic activity of caspase-10, and caspase-8, is redundant in gene induction. Thus, our data are consistent with a model in which both caspase-10 and cFLIP coordinately regulate CD95L-mediated signaling for death or survival.

INTRODUCTION

The apoptotic signaling cascade can be initiated by extrinsic or intrinsic stimuli. Extracellular death ligands, such as the cluster of differentiation 95 ligand (CD95L) (also known as FasL/Apo-1L) or TRAIL, bind to their respective receptors, most likely to preformed receptor trimers (Chan et al., 2000). Upon ligand binding, the adaptor protein FADD is recruited via its death domain to the receptor. The initiator caspase-8 then binds via its two death effector domains (DED) to the DED of FADD (Sprick et al., 2000). Proteins recruited upon receptor activation form a membrane-bound so-called death-inducing signaling complex (DISC) (Kischkel et al., 1995). After formation of the DISC, the large and small catalytic subunits of the caspase-8 homodimer are cleaved and activate downstream effector caspases (Hughes et al., 2009; Oberst et al., 2010). Additionally, active caspase-8 cleaves substrates, such as Bid, thereby connecting the extrinsic and intrinsic apoptotic pathway (Li et al., 1998). DISC-mediated caspase-8 cleavage is regulated by cFLIP, a DED containing caspase-like protein without protease activity (Irmler et al., 1997). Like caspase-8, cFLIP binds via its DEDs to the DISC and was shown to block caspase-8-mediated cell death (Leverkus et al., 2000; Siegmund et al., 2002; Wachter et al., 2004), although the long isoform of cFLIP ($cFLIP_L$) can also activate caspase-8 by mechanisms that are only now becoming clear (Boatright et al., 2004; Dohrman et al., 2005b; Hughes et al., 2016; Micheau et al., 2002; Yu et al., 2009).

The DISC is a complex synergy of recruited proteins, and recent studies have shown that DISC stoichiometry is different than prior models suggested, such that a single FADD molecule is able to recruit a multitude of caspase-8 molecules to the DISC via DED chain assembly (Dickens et al., 2012; Schleich et al., 2012). Death ligand-induced DISC formation is known to activate other signaling cascades beyond caspase-8. When caspase activity is blocked, death ligands can trigger necroptotic cell death, depending on the kinase activity of RIPK1 and RIPK3 (Feoktistova et al., 2011, 2012; Geserick et al., 2009; Tenev et al., 2011). In addition, DISC formation leads to NF-κB activation and cytokine gene induction (Choi et al., 2001; Farley et al., 2008; Park et al., 2003; Schmidt et al., 2015). Death ligand-mediated cytokine production occurs, in part, analogous to tumor

CrossMark

This is an open access article under the CC BY license (http://creativecommons.org/licenses/by/4.0/).

necrosis factor (TNF) signaling; RIPK1 is required for the activation of NF-κB by the degradation of IκBα (Peter et al., 2007). Of note, an interaction of RIPK1 with the DISC or soluble caspase-8 after receptor stimulation can primarily be detected whenever caspase activity is blocked (Cullen et al., 2013; Harper et al., 2001). However, it remains to be elucidated how RIPK1 is activated upon DISC formation. Interestingly, cFLIP is known to inhibit DISC-mediated gene induction, indicative of a critical but complex role of DISC-associated caspase-8 and cFLIP for regulation of cell death/gene induction (Kavuri et al., 2011; Wachter et al., 2004).

Caspase-10, a close homolog of caspase-8, is a highly conserved caspase throughout evolution, although absent in rodents (Eckhart et al., 2008; Sakamaki et al., 2015). It is recruited to and processed in the DISC (Kischkel et al., 2001; Sprick et al., 2002; Wang et al., 2001), and both caspase-8 and -10 share overlapping substrate specificities (Fischer et al., 2006). It is currently assumed that caspase-8 and caspase-10 have redundant functions in cell death signaling, but the ability of caspase-10 to substitute for caspase-8 has remained controversial. Due to the lack of caspase-10 in rodents, its function cannot be easily studied in vivo. Published experimental approaches to study its gene function have been mainly limited to overexpression studies that putatively derail the stoichiometry of DISC signaling (Mühlethaler-Mottet et al., 2011).

Here, we have identified an unanticipated role for caspase-10 in switching the CD95L-mediated response from caspase-8-induced cell death to activation of NF-κB and cell survival. We found that caspase-10 impedes caspase-8 activation within the CD95 DISC and that this occurs independently of cFLIP. Moreover, we demonstrate that caspase-10 promotes DISC-mediated gene induction and, independent of its catalytic activity, facilitates NF-κB signaling. Remarkably, we discover an indispensable scaffold function for caspase-8 in DISC formation. Thus, we show that, independent of its enzymatic activity, caspase-8 must bind to FADD to allow further recruitment of caspase-10 and/or cFLIP. Taken together, our data reveal that caspase-10 is a negative regulator of caspase-8-mediated cell death and instead supports CD95-induced gene induction.

RESULTS

Caspase-10 Inhibits CD95L-Induced Cell Death

To analyze the function of caspase-10 in CD95L-induced cell death, we performed small interfering RNA (siRNA)-mediated knockdown of caspase-10 in HeLa cells (Figure 1A). Whereas depletion of caspase-8 protected cells from CD95L-induced death, knockdown of caspase-10 reproducibly enhanced CD95-induced cell death. Under these conditions, cell death was caspase-8 mediated, as combined knockdown of caspase-8 and -10 fully protected cells from death induction. Knockdown of caspase-10 using four different siRNAs confirmed the inhibitory function of caspase-10 (Figure 1B). Moreover, HeLa cells expressing a doxycycline-induced caspase-10 short hairpin RNA (shRNA) were also significantly more sensitive to CD95L killing following knockdown of caspase-10 as measured by different assays (Figures 1C–1E and S1). The heightened sensitivity to CD95L was not due to an altered surface expression pattern of CD95 by knockdown of caspase-10 (data not shown). Next, we investigated other cell lines (diverse melanoma lines [SK-Mel, IGR, WK, and MC], B cell and T cell lymphoma [BJAB and Jurkat], and spontaneously transformed keratinocytes [HaCaT]) for the impact of caspase-10 on CD95L-induced cell death. As observed in HeLa cells, SK-Mel melanoma showed a significant sensitivity to CD95L after the depletion of caspase-10 (Figure 1F). In summary, we found that caspase-10 protects from CD95L-induced cell death in three out of eight cell lines examined.

Caspase-10 and cFLIP Independently Inhibit Caspase-8-Mediated Cell Death

Interestingly, all cell lines unaffected by the knockdown of caspase-10 showed a higher expression level of caspase-10 (examples shown for HaCaT/MC versus HeLa/SK-Mel in Figures 2A and 2B). Remarkably, protein levels after successful knockdown of caspase-10 in HaCaT or MC cells were comparable to endogenous levels present in HeLa or SK-Mel (Figure 2A). This differential stoichiometry of caspase-10 was also reflected in the DISC (Figure S2). We initially hypothesized that the low levels of caspase-10 remaining in these "high expressors" was sufficient to inhibit CD95L-induced cell death. However, a closer look at the expression levels of caspase-10 and cFLIP in HaCaT cells showed a reciprocal counter-regulation of cFLIP after inducible knockdown of caspase-10 (Figures 2C and 2D). Moreover, changes in cFLIP expression were also reflected in the DISC with an increased level of cleaved cFLIP p43 bound to the receptor (Figure 2C). In contrast, caspase-8 or FADD levels in the DISC were unchanged (Figure 2C). These data revealed a close relationship between cFLIP and caspase-10 for inhibition of cell death. To corroborate this assumption, we next combined the knockdown of caspase-10 and cFLIP in HaCaT cells. As shown in Figure 2D, combination of caspase-10 and cFLIP knockdown increased the sensitivity of cells to CD95L (Figures 2D and S3A, dark red columns). As cFLIP expression in HaCaT is very low (Figure 2A), we aimed to extend the data to cell lines with higher cFLIP levels. We thus performed siRNA-mediated knockdown of caspase-10 and cFLIP in HeLa cells. Depletion of either caspase-10 (Figure 2E, light red columns) or cFLIP (Figure 2E, black columns) led to a dramatic increase in CD95-induced cell death. Notably, similar to our data in HaCaT cells, combined knockdown of caspase-10 and cFLIP further increased sensitization to CD95L when compared to caspase-10/cFLIP knockdown alone (Figures 2E and S3B, dark red columns). Taken together, our data showed that caspase-10 is a negative regulator of CD95L-induced cell death, independent of cFLIP. However, at least in HaCaT cells, loss of caspase-10 is compensated by increased expression of cFLIP.

Caspase-10 Impedes DISC-Mediated Caspase-8 Activation

Next, we examined whether caspase-10 modulates assembly of the DISC by characterizing DISC formation in the presence or absence of caspase-10. Upon caspase-10 depletion in HeLa cells, we observed an enrichment of full-length and p43/41 caspase-8 cleavage fragments in the DISC (Figure 3A). Quantification of the ratio of caspase-8 (full length; p43/41) relative to

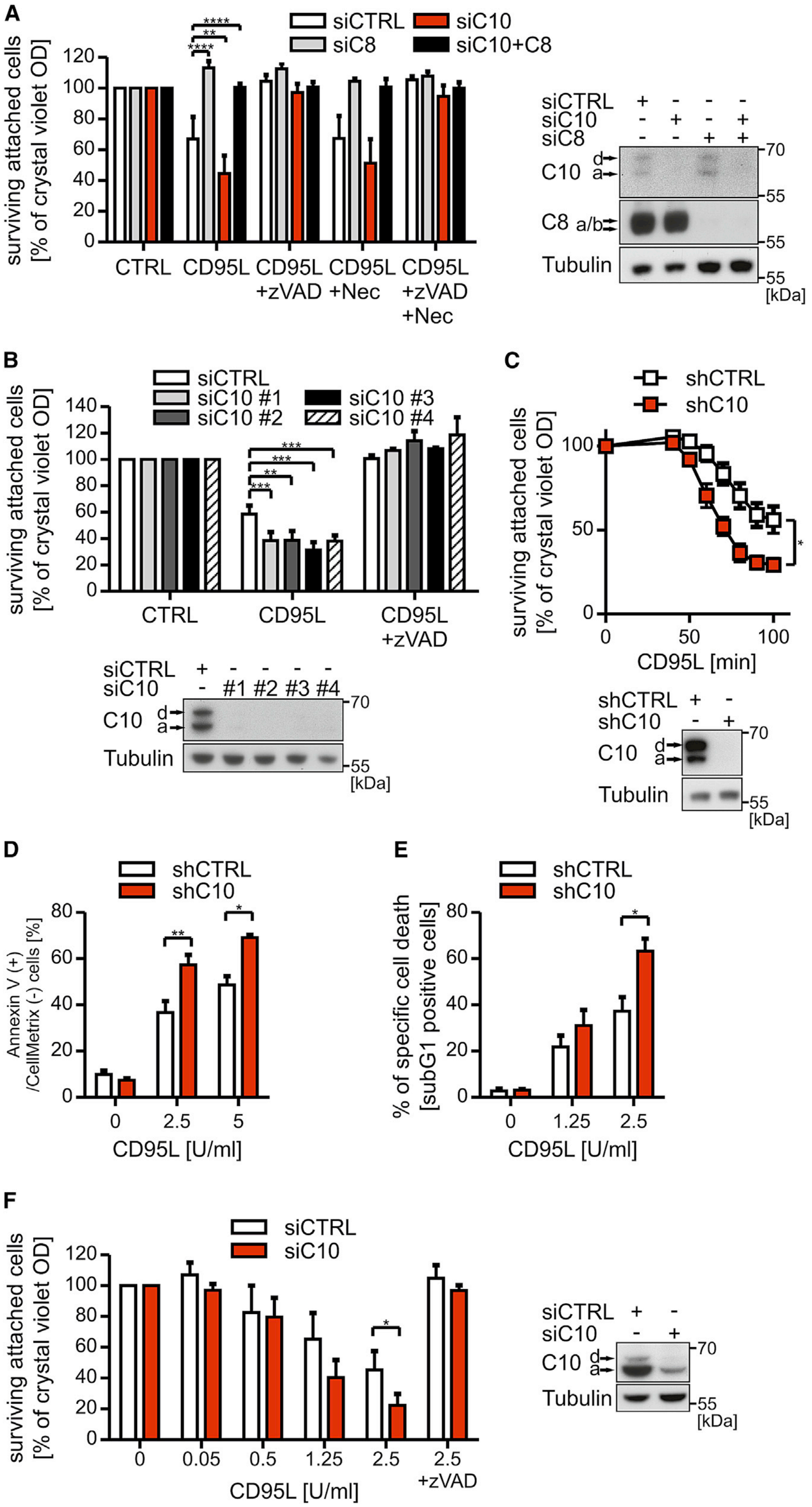

Figure 1. Caspase-10 Impairs CD95L-Induced Cell Death

(A) HeLa cells were transfected with caspase-10 (siC10), caspase-8 (siC8), the combination of both, or control siRNA (siCTRL). After 72 hr, triplicates were preincubated with 10 μM zVAD-fmk (zVAD) or 50 μM Necrostatin-1 (Nec) or the combination of both for 1 hr followed by stimulation of 1 U/mL CD95L-Fc for 16–20 hr. Cell viability was analyzed by crystal violet (CV) staining. Knockdown efficiency was controlled by western blotting (WB).

(B) HeLa cells were transfected with four different caspase-10 (siC10) or control (siCTRL) siRNAs. After 72 hr, corresponding triplicates were preincubated with 10 μM zVAD for 1 hr followed by stimulation of 1 U/mL CD95L-Fc for 16–20 hr. Cell viability was analyzed by CV staining. Knockdown efficiency was controlled by WB.

(C–E) HeLa cells inducibly expressing a shRNA against caspase-10 (shC10) or control shRNA (shCTRL) were treated with 0.5 μg/mL doxycycline for 72 hr. (C) Cells were stimulated in triplicates for the indicated time points with 2 U/mL CD95L-Fc. Cell survival was analyzed as described above. Knockdown efficiency was controlled by WB. (D) Cells were stimulated with the indicated concentrations of CD95L-Fc for 3 hr. Apoptotic cells (annexin V positive and CellMetrix negative) were measured by analyzing the externalization of phosphatidylserine and plasma membrane integrity by Pacific Blue Annexin V plus CellMetrix Green Live/Dead Stain using flow cytometry. A representative experiment is shown in Figure S1A. (E) Cells were stimulated with the indicated CD95L-Fc concentrations for 7 hr. DNA degradation was quantified by flow cytometry using propidium iodide (PI) staining for sub G1 populations. A representative experiment is shown in Figure S1B.

(F) SK-Mel cells were transfected with caspase-10 (siC10) and control siRNA (siCTRL). Seventy-two hours later, corresponding triplicates were pretreated with 10 μM zVAD for 1 hr followed by stimulation with the indicated concentrations of CD95L-Fc for 4 hr. Cell viability was analyzed by CV staining. Knockdown efficiency was controlled by WB.

Each graph/diagram represents mean values ± SEM of at least three independent experiments. Significance levels (p values) were measured by Student's t test (C–F) or two-way ANOVA test (A and B; *$p < 0.05$; **$p < 0.01$; ***$p < 0.001$; ****$p < 0.0001$).

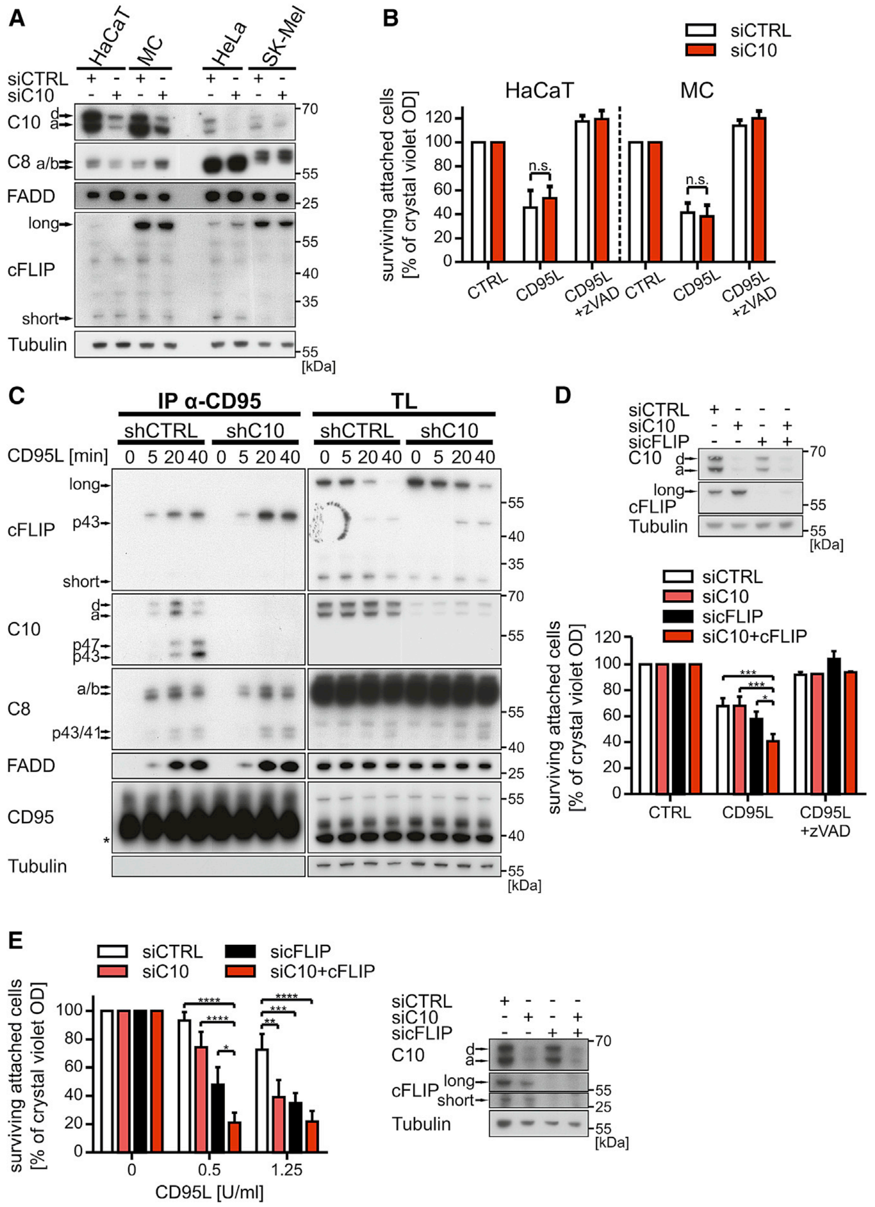

Figure 2. Both cFLIP and Caspase-10 Block Caspase-8-Mediated Cell Death

(A) Different cell lines were transfected with siC10 or siCTRL. After 72 hr, knockdown efficiency and proteins involved in DISC signaling were analyzed by WB. Cell lines with high expression of caspase-10 (HaCaT and MC) were compared to low-expressing cell lines (HeLa and SK-Mel).

(B) HaCaT and MC cells were treated with siC10 and siCTRL as described in (A). Triplicates were pre-treated with 10 μM zVAD-fmk (zVAD) for 1 hr followed by stimulation of 0.1 (HaCaT) and 2.5 (MC) U/mL CD95L-Fc for 16–20 hr (HaCaT) or 4 hr (MC). Cell viability was analyzed by CV staining.

(C) shC10 or shCTRL expression was induced in HaCaT cells by the addition of 0.5 μg/mL doxycycline (Doxy) for 72 hr. Cells were stimulated with 1 U/mL CD95L-Fc for the indicated time points. CD95 was immunoprecipitated from cell lysates (TL), and co-precipitated proteins were analyzed by WB. The asterisk marks a non-specific band.

(D) HaCaT cells were transfected with siC10, sicFLIP, the combination of both, or siCTRL. After 48 hr, cells were pre-treated in triplicates with 10 μM zVAD for 1 hr and further stimulated for 4 hr with 0.5 U/mL CD95L-Fc. Cell viability was assayed using CV staining. Knockdown efficiency was controlled by WB.

(E) HeLa cells were treated with siRNA as described in (D), stimulated with the indicated concentrations of CD95L-Fc, and analyzed for their cell viability by CV staining. Knockdown efficiency was controlled by WB.

Each diagram represents mean values ± SEM of three independent experiments. Significance levels (p values) were measured by Student's t test (B) or two-way ANOVA test (D and E; $*p < 0.05$; $**p < 0.01$; $***p < 0.001$; $****p < 0.0001$; n.s., not significant).

FADD within the DISC confirmed an enhancement of DISC-associated caspase-8 in the absence of caspase-10 (Figure 3B). In contrast, the recruitment of cFLIP to the DISC was unaffected (Figure 3B). Taken together, these findings support our conclusion of a negative regulatory function for caspase-10 in death signaling.

To analyze the proposed inhibitory function of caspase-10 in more detail, we reconstituted the CD95 DISC in a cell-free system using recombinant protein as previously described (Hughes et al., 2009). Strikingly, we observed a concentration-dependent inhibition of caspase-8 by caspase-10 (Figure 3C). Caspase-10 blocked processing of caspase-8 in the DISC and reduced DISC-associated IETDase activity (Figure 3C). Furthermore, our data confirm the ability of $cFLIP_L$ to activate caspase-8, as well as the co-operative and hierarchical binding model of caspase-8 and cFLIP (Hughes et al., 2016). As shown in Figure 3C, recruitment of $cFLIP_L$ to the complex is enhanced by the presence of caspase-8. Intriguingly, our data suggest a similar model of co-operative binding can now be linked to caspase-10. In the absence of caspase-8, recruitment of caspase-10 to the DISC is inefficient and, importantly, IETDase activity is absent (Figure 3C). Taken together, our data demonstrate that caspase-10 impedes caspase-8 processing, thereby reducing DISC-associated caspase activity.

Caspase-8 Is Indispensable for the Assembly of the CD95 DISC

To further study the co-operative binding of caspase-10 and caspase-8 to the CD95 DISC and to explore whether caspase-10 can substitute for caspase-8 in the native DISC, we next generated caspase-8 knockout HeLa cells using CRISPR/Cas9-mediated recombination. Two independent C8 CRISPR cell clones (C8 CRISPR), generated with two different

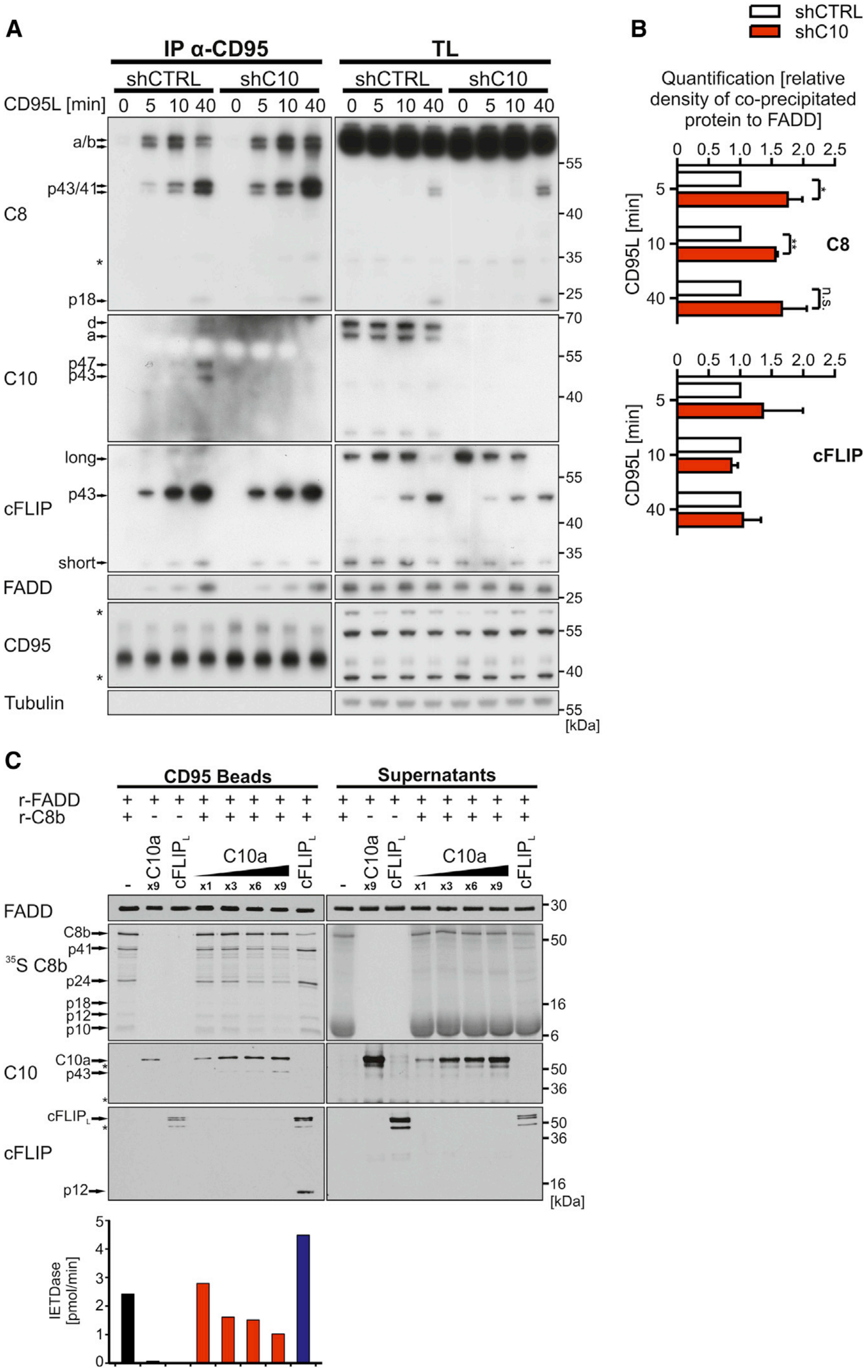

Figure 3. Caspase-10 Impairs Recruitment and Activation of Caspase-8 in the DISC

(A) shCTRL or shC10 expression was induced by 0.5 μg/mL doxycycline in HeLa cells. After 72 hr, cells were incubated for the indicated time points with 2 U/mL CD95L-Fc. CD95 was immunoprecipitated from cell lysates (TL), and DISC-associated proteins were analyzed for DISC recruitment by western blotting. The asterisks mark non-specific bands.

(B) Relative density of co-precipitated caspase-8 (full-length a/b and p43/41) and cFLIP p43 from the CD95L kinetic shown in (A) was quantified in respect to co-precipitated FADD and normalized to shCTRL cells. Shown are mean values ± SEM of three independent experiments. Significance levels (p values) were measured by Student's t test (*$p < 0.05$; **$p < 0.01$).

(C) A functional CD95 DISC was assembled using GST-CD95 intracellular domain (10 μg), recombinant FADD (r-FADD) (5 μg), and ^{35}S-labeled recombinant procaspase-8b (r-C8b) (85 μL) at 20°C for 16 hr in the absence or presence of recombinant cFLIP$_L$ or increasing amounts of recombinant procaspase-10a (C10a) (25–225 μL; indicated as 1×, 3×, 6×, and 9×). Beads and supernatants were analyzed by SDS-PAGE and autoradiography for ^{35}S C8b and immunoblotted for FADD, caspase-10, and cFLIP. Beads were additionally assayed for caspase-8 activity (IETDase). Control CD95 DISCs contained C10a or cFLIP$_L$ in the absence of r-C8b. Data shown are representative of three independent experiments. Asterisks mark non-specific bands.

guide RNAs (gRNAs) (Figure S4), were fully protected from CD95L-induced death irrespective of the expression of caspase-10 (Figure 4A). Intriguingly, C8 CRISPR cells also had repressed cFLIP/caspase-10 levels as compared to parental cells (Figures 4A and 4B, right panels). We next studied DISC composition in the absence of caspase-8. In line with our reconstituted DISC model, we observed that, despite effective precipitation of CD95, the native DISC isolated from C8 CRISPR cells completely lacked cFLIP and caspase-10 (Figure 4B), and strikingly, only weak levels of FADD were detected upon prolonged exposure of the blots. To exclude clonal artifacts during generation of C8 CRISPR cells, we next reconstituted caspase-8a and its respective active site mutant (ASM) in C8 CRISPR cells by inducible overexpression. When these cells were analyzed for CD95L-induced cell death, re-expression of caspase-8, but not its ASM, conferred sensitivity to CD95L-induced cell death, despite a lower overall expression level of the enzymatically active caspase-8a (Figure 5A). Intriguingly, reconstitution of caspase-8 protein restored the recruitment of cFLIP, caspase-10, and, importantly, FADD in the DISC independent of the enzymatic activity of caspase-8 (Figure 5B). The caspase-8 ASM, however, fully protected cleavage of caspase-8, caspase-10, or cFLIP, indicative of the critical importance of caspase-8 enzymatic activity for induction of cell death, but not initial recruitment of DED proteins. Thus, our data reveal a central role for caspase-8 in formation of the CD95 DISC and places caspase-8 upstream of both cFLIP and caspase-10 in DISC signaling.

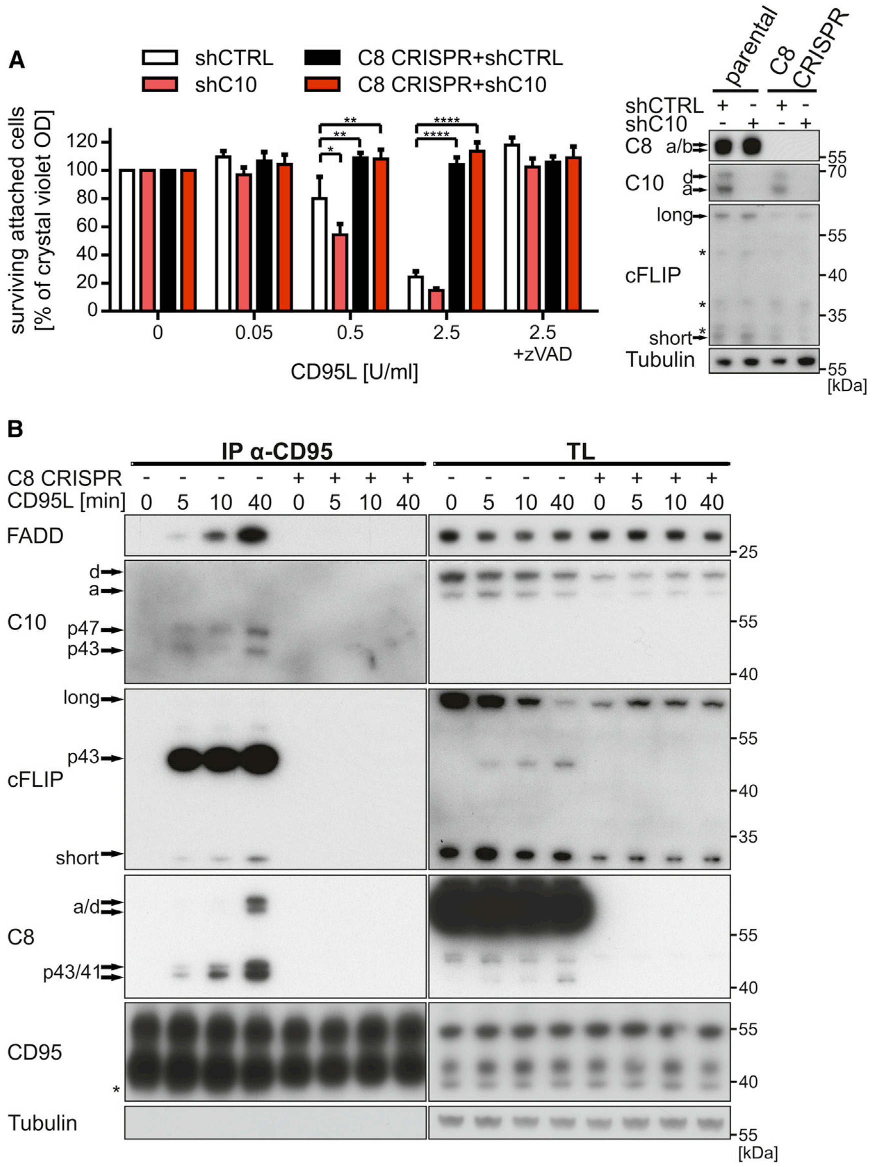

Figure 4. CD95 DISC Formation Requires Caspase-8

(A) Parental and caspase-8-deficient (C8 CRISPR) HeLa cells were treated for 72 hr with 0.5 μg/mL doxycycline to induce the expression of either control or caspase-10 shRNA. Cells were pretreated for 1 hr with 10 μM zVAD-fmk (zVAD) followed by stimulation with the indicated concentrations of CD95L-Fc for 4 hr. Knockdown efficiency of caspase-10 as well as the knockout of caspase-8 was controlled by WB. Cell viability was analyzed in triplicates by crystal violet staining. Shown are mean values ± SEM of three independent experiments. Significance levels (p values) were measured by two-way ANOVA test (*p < 0.05; **p < 0.01; ****p < 0.0001).

(B) CD95 DISC formation was analyzed in parental and HeLa C8 CRISPR cells. CD95 was immunoprecipitated from cell lysates (TL) after stimulation with 2 U/mL CD95L-Fc for the indicated time points. DISC-associated proteins were analyzed by WB. Asterisks mark non-specific bands.

Caspase-10 Promotes CD95L-Mediated NF-κB Activation and Gene Induction

We thus far explored caspase-10 for its impact on DISC-mediated cell death signaling. However, the function of the enzymatic activity of caspase-10 remains unclear. We reasoned that another function of caspase-10 might be related to the known gene-inductive properties exerted by CD95 stimulation (Cullen et al., 2013; Wallach et al., 1999). Moreover, it is well known that zVAD strongly supports DISC-mediated gene induction (Harper et al., 2001), and we have also previously reported that QVD allows for TRAIL-induced gene activation (Kavuri et al., 2011). However, when we compared zVAD and QVD for their impact on CD95L-mediated *interleukin-8* (*IL-8*) mRNA expression, *IL-8* induction was unaffected by QVD (Figure S5A). Furthermore, we observed that QVD was, firstly, inefficient in blocking CD95L-induced cell death compared to zVAD (Figure S5B) and, secondly, only partially blocked processing of caspase-8 after DISC stimulation (Figure S5C). Therefore, we characterized the role of caspase-10 in death-receptor-mediated gene induction in HeLa cells by microarray analysis in the presence of zVAD to achieve maximal gene expression. We observed that caspase-10 knockdown did not affect the subset of genes induced upon CD95L stimulation; rather it impacts on the amplitude of induction of a variety of NF-κB-induced target genes (Table S1). Of note, we identified a number of CD95L-induced genes to be deregulated by knockdown of caspase-10 (Table S1, light orange), with three genes exhibiting >25% repression of gene induction (Table S1, dark orange). Thus, we aimed to verify selected genes in more detail and importantly demonstrated that loss of caspase-10 significantly repressed IL-8 secretion after CD95L stimulation (Figure 6A). Furthermore, we analyzed the impact of caspase-10 on six CD95L-induced genes via real-time qPCR and observed that caspase-10 knockdown significantly reduced CD95L-mediated gene induction by 20%–50% in all targets examined (Figure 6B). As described for TNF-R-signaling, CD95L-induced gene induction is driven by multiple protein kinases, including the IKK complex, JNK, or p38 mitogen-activated protein (MAP) kinases (Cullen et al., 2013; Wallach et al., 1999). To study the impact of caspase-10 on these kinases, we generated caspase-10 knockout (C10 CRISPR) HeLa cells, which confirmed the heightened sensitivity to CD95L stimulation observed by knockdown approaches (Figure S6A). Whereas we failed to detect obvious differences in the phosphorylation status of JNK or p38 MAP kinase (MAPK) under conditions with or without

OPEN ACCESS
CellPress

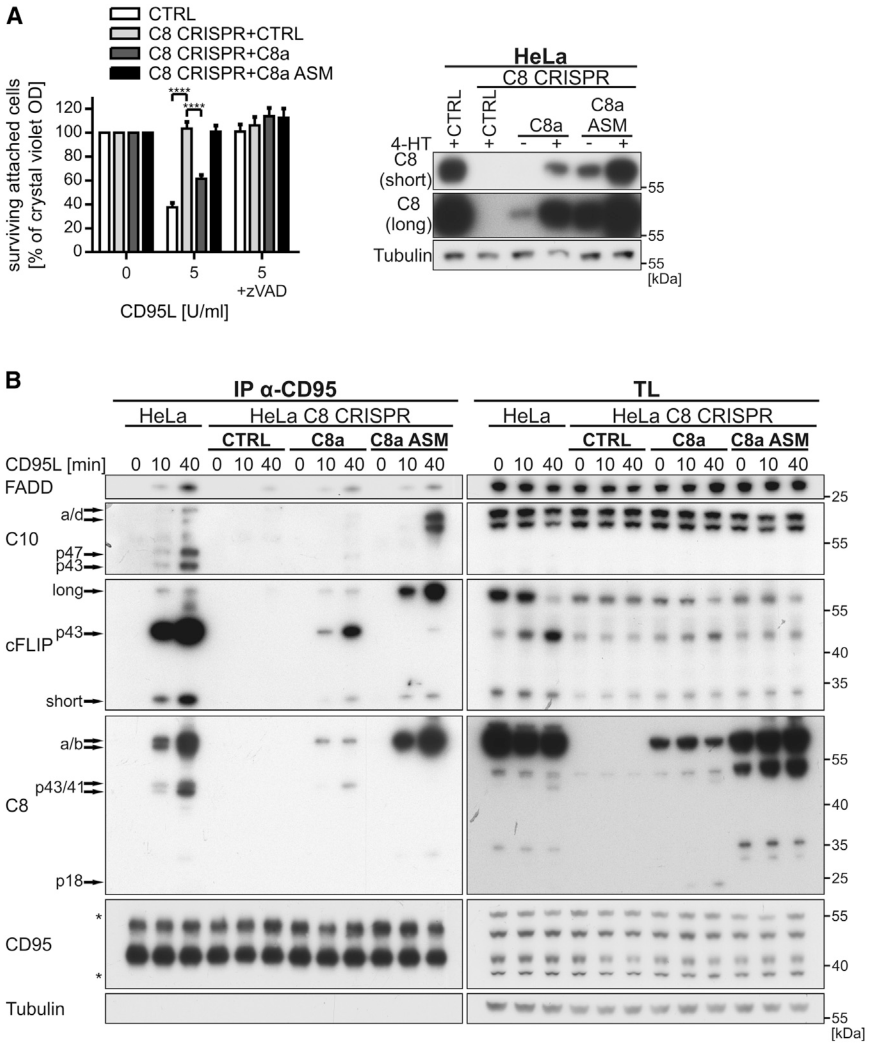

Figure 5. Caspase-8 Scaffold Function Is Indispensable for DISC Recruitment of DED Proteins

Caspase-8a (C8a) and its respective active site mutant (C8a ASM) were reconstituted in caspase-8-deficient HeLa (C8 CRISPR) cells.

(A) Parental and HeLa C8 CRISPR cells either overexpressing the empty vector (CTRL), C8a, or C8a ASM were treated with 10 nM 4-hydroxy-tamoxifen (4-HT) for 6 hr to induce the expression of the respective constructs. Cells were pre-treated with 10 μM zVAD-fmk (zVAD) for 1 hr followed by stimulation with 5 U/mL CD95L-Fc for 3 hr. Cell viability was analyzed in triplicates by crystal violet staining. Shown are mean values ± SEM of three independent experiments. Significance levels (p values) were measured by two-way ANOVA test (****$p < 0.0001$). Expression of C8a and C8a ASM were analyzed by western blotting.

(B) As described in (A), C8a was reconstituted in HeLa C8 CRISPR cells for 6 hr by 4-HT. Cell lines were stimulated with 2 U/mL CD95L-Fc for the indicated time points. CD95 was immunoprecipitated from cell lysates (TL) and analyzed for DISC-associated proteins. Asterisks mark non-specific bands.

caspase-10 expression (data not shown), CD95L-mediated IκBα degradation/phosphorylation was inhibited in C10 CRISPR cells (Figures 6C and S6B).

Corroborating our findings of an upstream role for caspase-8 in DISC formation, CD95L-induced cytokine gene induction was fully absent in C8 CRISPR cells. In contrast, reconstitution with caspase-8a/ASM allowed for *IL-8* mRNA induction, albeit to a lesser extent than parental cells (Figure 6D). To accommodate for differing expression levels between re-expressed wild-type and ASM caspase-8, we examined *IL-8* mRNA induction relative to caspase-8 expression in parental cells (Figures 6D and S6C). Moreover, C10 CRISPR cells confirmed our previous data obtained by inducible knockdown. In the absence of caspase-10, *IL-8* mRNA induction was reduced following CD95L stimulation (Figure 6E), whereas reconstitution with wild-type or ASM caspase-10a increased *IL-8* induction in these cells (Figures 6E and S6D). Our experiments using reconstituted caspase-8a ASM, as well as caspase-10a ASM, clearly showed that caspase-mediated cytokine induction occurs independently of catalytic activity (Figures 6D and 6E). Thus, caspase-10 and 8 promote DISC-mediated gene induction, revealing an intricate balance of gene-inducing/death-promoting abilities of these two DISC-associated tandem DED proteins.

DISCUSSION

To date, studies about DISC signaling have focused on the function of caspase-8 and its regulator cFLIP. In contrast, the role of caspase-10 is more controversial and less understood. The interplay of the tandem DED proteins caspase-8 and -10 and cFLIP is complex; in our study, downregulation of one of these proteins frequently resulted in a rapid counter-regulation of at least one of the other tandem DED proteins. For example, loss of caspase-8 leads to a downregulation of cFLIP (Figures 4 and 5), HaCaT cells counter-regulate the loss of caspase-10 by upregulation of cFLIP (Figure 2), and caspase-8-deficient Jurkat cells were shown to downregulate caspase-10 (Sprick et al., 2002). These phenomena occurred remarkably quickly during cell culture, thus showing how closely these proteins are inter-linked and critical for cell survival. Notably, caspase-10/8 and cFLIP are located on the same genetic locus (2q33-q34), and co-regulated genes tend to be clustered in the same genetic neighborhood (Michalak, 2008). Taken together, our findings underscore the necessity to simultaneously study all tandem DED proteins.

We focused our attention on the role of caspase-10 and strikingly found that this caspase is a negative regulator of DISC-mediated apoptosis (Figure 1). Thus far, caspase-10 was

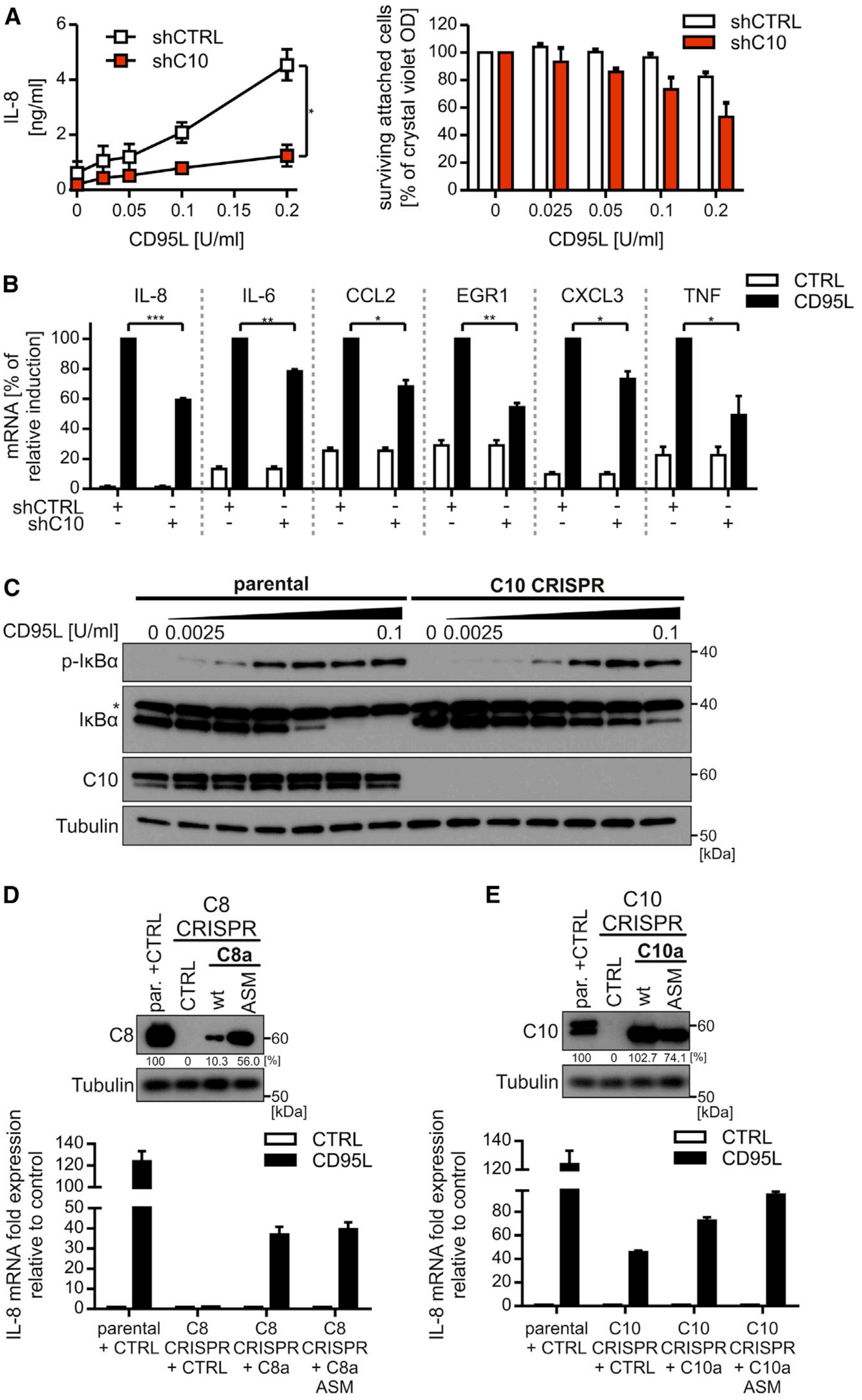

Figure 6. Caspase-10 Promotes CD95L-Mediated Gene Induction

(A and B) HeLa cells expressing shC10 or shCTRL were treated for 72 hr with 0.5 μg/mL doxycycline.
(A) Duplicate wells were stimulated in media containing 0.5% FCS with the indicated concentrations of CD95L-Fc for 24 hr. Supernatants were analyzed for secreted interleukin-8 (IL-8) by ELISA. Cell viability was assayed using crystal violet staining. (B) HeLa ± shC10 cells were pre-starved for 4 hr in media containing 0.5% FCS followed by treatment with 10 μM zVAD-fmk (zVAD) for 1 hr. Cells were stimulated with 0.1 U/mL CD95L-Fc for 3 hr. RNA was isolated, reverse transcribed to cDNA, and mRNA expression levels of *IL-8*, *IL-6*, *CCL2*, *EGR1*, *CXCL3*, and *TNF* were analyzed by real-time qPCR.
(C) Parental and caspase-10-deficient (C10 CRISPR) HeLa cells were starved and pre-treated with zVAD as described in (B). Cells were stimulated with CD95L-Fc (0.0025, 0.005, 0.01, 0.025, 0.05, or 0.1 U/mL) for 3 hr. IκBα phosphorylation as well as degradation and caspase-10 knockout were analyzed by western blotting. Asterisks mark non-specific bands.
(D) Parental and caspase-8-deficient (C8 CRISPR) HeLa cells were treated with 10 nM 4-HT for 6 hr in media containing 0.5% FCS to induce the expression of either control plasmid or caspase-8a (expression of caspase-8a ASM was achieved in the absence of induction via 4-HT). Cells were stimulated with zVAD and CD95L-Fc as described in (B) and analyzed for *IL-8* mRNA expression by real-time qPCR. Caspase-8 expression was quantified after reconstitution and compared to parental HeLa cells as indicated in the western blots. Relative *IL-8* mRNA induction has been calculated with respect to caspase-8 expression.
(E) Parental, C10 CRISPR, and reconstituted caspase-10a/ASM HeLa cells were treated as described in (D) (expression of wild-type caspase-10 was achieved in the absence of induction via 4-HT). Cells were stimulated with zVAD and CD95L-Fc as described in (B) and *IL-8* mRNA expression analyzed. Relative caspase-10 expression and *IL-8* induction has been calculated as described in (D) (raw data for D and E are shown in Figures S6C and S6D).
Each graph/diagram represents mean values ± SEM of three independent experiments. Significance levels (p values) were measured by Student's t test (*$p < 0.05$; **$p < 0.01$; ***$p < 0.001$).

reported to be a pro-apoptotic initiator caspase, similar to caspase-8 (Engels et al., 2005; Fischer et al., 2006). Importantly, our data exclude a pro-apoptotic function of caspase-10, independent of the cell line or the experimental setting used (siRNA-/shRNA-mediated knockdown or gene knockout). Significantly, our reconstituted DISC approach clearly demonstrates the negative impact of caspase-10 on caspase-8 in a cell-free system (Figure 3). In line with our data and a more complex role of caspase-10, a recent study has proposed a pro-survival function of endogenous caspase-10, as it was shown to inhibit autophagic cell death in multiple myeloma cell lines (Lamy et al., 2013).

When we studied the impact of caspase-10 mechanistically, caspase-10 was shown to impair recruitment/processing of

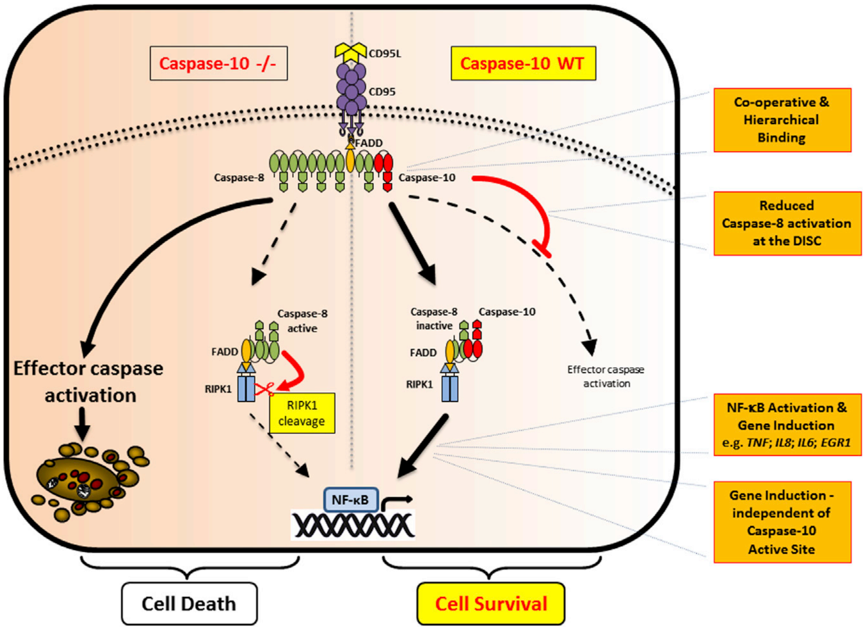

Figure 7. A Dual Role for Caspase-10 in DISC Signaling

In this model, FADD binding to the CD95 receptor initiates formation of the DISC. Caspase-8 binding is then required to stabilize the FADD receptor association and enable subsequent recruitment of caspase-10. The presence or absence of caspase-10 defines the amplitude of the cell death response. In the absence of caspase-10, caspase-8 DED chain assembly strongly activates downstream effector caspases to induce apoptosis. Simultaneously, caspase-8 promotes RIPK1 activation by mechanisms that to date have not been elucidated. Next, NF-κB dimerizes and localizes to the nucleus to induce cytokine gene expression. In the presence of caspase-10, DISC-mediated caspase-8 activity and subsequent cell death are reduced. Moreover, caspase-10, together with caspase-8, favors RIPK1 activation and NF-κB-mediated gene induction, independent of caspase-10/caspase-8 catalytic activity. Thus, taken together, caspase-10 switches the cell death response in favor of cell survival and cytokine gene expression.

caspase-8 in the DISC (Figure 3). Previous mass spectrometry analysis of the native CD95- and TRAIL DISC demonstrated that FADD is clearly sub-stoichiometric as compared to caspase-8, leading to a paradigm-changing model of DED chain elongation that is mainly driven by caspase-8 (Dickens et al., 2012; Schleich et al., 2012). In marked contrast, the ratio of caspase-8 to caspase-10 or cFLIP protein levels, respectively, are much lower and until recently have not been examined in detail. A recent report has suggested that short DED proteins regulate caspase-8 activation in DED chains (Schleich et al., 2016). Moreover, we have recently shown that overexpression of $cFLIP_S$ disrupts caspase-8 chain assembly (Hughes et al., 2016). Based on the data we present here, we hypothesize that caspase-10 may also disrupt caspase-8 chain elongation (Figure 7), but this will require further investigation. In line with this assumption, overall levels of DISC-bound caspase-8 were reduced in the presence of caspase-10 (Figure 3). Furthermore, IETDase activity of the reconstituted DISC was repressed by caspase-10 (Figure 3). Interestingly, DISC-associated caspase-10 was unable to activate IETDase in the absence of caspase-8 (Figure 3), despite reports that in vitro dimerized caspase-10 catalytic subunits harbor IETDase activity (Wachmann et al., 2010). However, this finding suggests another possibility for caspase-10 in terms of inhibiting cell death. Caspase-10 and caspase-8 could potentially form heterodimers with reduced or absent activity. In this regard, heterodimerization between caspase-8/10 and $cFLIP_L$ has been reported (Boatright et al., 2004; Yu et al., 2009), but it is highly controversial whether the resulting heterodimer has pro- or anti-apoptotic functions (reviewed in van Raam and Salvesen, 2012).

In line with our very recent report on cFLIP (Hughes et al., 2016), our studies demonstrate that caspase-8 is located upstream of both caspase-10 and cFLIP in the cell death pathway with critical relevance for DISC formation (Figures 4 and 5). Our re-expression studies with wild-type/ASM caspase-8 in C8 CRISPR cells restored recruitment of caspase-10, cFLIP, and importantly FADD to the DISC, independent of the catalytic activity of caspase-8 (Figure 5). Moreover, we show that FADD association within the DISC is stabilized by caspase-8 binding (Figures 4 and 5). Thus, our data demonstrate that a scaffold function of caspase-8 is both necessary and critical for DISC formation. However, in contrast to its homolog caspase-8, caspase-10 is not essential for DISC signaling. Altogether, our studies reveal that caspase-10 and cFLIP negatively regulate cell death signaling within the DISC but that this occurs downstream of caspase-8 recruitment. This finding is in contrast to previous reports made in caspase-8-deficient Jurkat cell lines, in which caspase-10 was recruited to the DISC in the absence of caspase-8 (Sprick et al., 2002). Importantly, we observed a weakly expressed truncated form of caspase-8 in caspase-8-deficient Jurkat cells (data not shown). Thus, because low levels of caspase-8 can restore caspase-10 and cFLIP recruitment to the DISC (Figure 5), these findings likely explain the previous results with caspase-8-deficient Jurkat cells (Sprick et al., 2002). Therefore, some of the collected knowledge about apoptotic and gene-inductive signaling gained from caspase-8-deficient Jurkat cells has to be critically re-evaluated.

In addition to the apoptosis-inducing machinery, DISC-mediated gene induction is an important signaling pathway that is activated concomitant to apoptotic cell death (Peter et al., 2007) but is most frequently observed under conditions independent of apoptotic caspase-8 activation, e.g., in the presence of broad spectrum caspase inhibitors (Harper et al., 2001; Kavuri et al., 2011; Leverkus et al., 2003). Importantly, we observed that QVD failed to support DISC-mediated gene induction and that it is inefficient in blocking CD95L-induced caspase-8 cleavage and cell death (Figure S5). Very recently, it has been shown that QVD, in contrast to zVAD, is a weak inhibitor of the

caspase-8/cFLIP$_L$ heterodimer, whereas both almost equally block the caspase-8 homodimer (Brumatti et al., 2016). Studies on the function of cFLIP$_L$ in the activation of NF-κB upon DISC formation are contradictory. It has been reported that the cFLIP p43 fragment promotes the recruitment of TRAF2 and RIPK1 to the caspase-8/cFLIP heterodimer, resulting in more efficient activation of NF-κB (Dohrman et al., 2005a). In contrast, several studies reported an inhibitory function of cFLIP$_L$ in DISC-mediated gene induction (Kavuri et al., 2011; Kreuz et al., 2004). This clearly needs to be further examined but puts the caspase-8/cFLIP heterodimer in the spotlight for DISC-induced NF-κB activation. Strikingly, our data now show that caspase-10 promotes DISC-mediated cytokine expression by enhancing IκBα degradation/phosphorylation (Figure 6; Table S1). Whereas the molecular mechanisms of DISC-mediated gene induction are largely unknown, our studies show that the scaffold function of caspase-8 is essential for DISC formation as well as NF-κB activation (Figure 6); moreover, the catalytic activity of caspase-8 and -10 is redundant for NF-κB activation and subsequent cytokine induction. However, it still remains to be elucidated how and where caspase-8 and -10 activate RIPK1 to initiate the phosphorylation of IκBα.

Our data for CD95L demonstrate that caspase-10 shifts the apoptotic cell death response following DISC formation to the activation of NF-κB and cell survival (Figure 7), both beneficial features for tumor cells. Thus, caspase-10 may have tumorigenic properties. Chronic inflammatory gene expression favors tumor formation and progression (Chai et al., 2015), and CD95 signaling has been shown in several studies to favor tumor growth and invasiveness (Barnhart et al., 2004; Chen et al., 2010). Thus, our data hint at the possibility of modulating caspase-10 expression as a therapeutic oncological target; repression of caspase-10 reduces cytokine expression (Figure 6) and favors apoptosis (Figure 1), potentially resulting in impaired tumor growth. Whether DISC-mediated gene induction is responsible for CD95 dependency of cancer cells remains unanswered. However, we have previously demonstrated that primary keratinocytes activate NF-κB upon stimulation of the TRAIL DISC, and they are 5-fold less sensitive to death-ligand-induced apoptosis than transformed keratinocytes (Kavuri et al., 2011; Leverkus et al., 2000). Thus, gene induction is potentially much more relevant in DISC signaling than currently assumed.

Here, we reveal the elusive, and in part controversial, role of caspase-10 in DISC signaling. Our discovery of caspase-10 as a negative regulator of cell death and a facilitator of gene induction separates the function of cFLIP and caspase-10 in the DISC. Crucially, we demonstrate that caspase-8 binding via FADD to the receptor is an indispensable initiating step in DISC formation and NF-κB activation. Moreover, our data clearly delineate that caspase-10 is not an initiator of DISC-induced cell death signaling as previously believed. Instead, caspase-10 and caspase-8 scaffold function promote DISC-mediated gene induction, revealing an intricate balance of gene-inducing and death-protecting abilities of the different DISC-associated tandem DED proteins (Figure 7). These findings change our current understanding of extrinsic apoptotic signaling and open new possibilities in terms of signaling via other caspase-8 and -10 and cFLIP-containing complexes, such as the ripoptosome or TNF complex (Feoktistova et al., 2011; Micheau and Tschopp, 2003; Tenev et al., 2011).

EXPERIMENTAL PROCEDURES

Supplemental Experimental Procedures contain details of materials, antibodies, siRNA transfection, cytotoxicity assays, western blot analysis, IL-8 ELISA, and primer sequences used for real-time qPCR.

Generation of Stable Cell Lines

To generate stable cell lines that inducibly overexpress caspase-8a/10a and their respective active site mutants by addition of 4-hydroxytamoxifen, cells were transduced with a pF GEV16 Super PGKHygro as previously described (Diessenbacher et al., 2008). Viral vectors inducibly expressing a shRNA against caspase-10 (V3THS_394573) or the respective control shRNA (TRIPZ inducible lentiviral non-silencing shRNA control) via the pTRIPZ lentiviral shRNAmir system by addition of doxycycline were purchased from GE Healthcare and used for production of lentiviral particles using a second-generation packing system. In brief, HEK293T cells were transfected with 3 μg pMD2.G, 7.5 μg pSPax2, and 3 μg pcDNA3.1/p35 of lentiviral packaging vectors together with the transfer vector pF 5 × UAS W SV40 Puro, which expresses caspase-8 in a Gal4-dependent fashion. Caspase-8a/ASM was subcloned from pcDNA3.1 (Hughes et al., 2009) and caspase-10a/ASM from pEF6/V5-His-TOPO into lentiviral vector system using standard cloning procedures and verified by sequencing. Supernatants were harvested 24 and 48 hr post-transfection and filtered (0.45 μm filter; GE Healthcare). Viral particles were added to the cells with 5 μg/mL polybrene, and cells were spin infected for 1.5 hr at 30°C. Antibiotic-resistant stable cell lines were selected in 300 μg/mL hygromycin and/or 1 μg/mL puromycin for 3–7 days.

Caspase-8/10-deficient HeLa cells were generated using the CRISPR/Cas9 system. HeLa cells were transiently transfected using Lipofectamine LTX Reagent with PLUS Reagent (Thermo Fisher Scientific) according to manufacturer's recommendations. HeLa cells were cotransfected with the pMA-T vector (carrier of the cassette-U6-gRNA(casp8)-TTTTT; Life Technologies) and hCas9-pcDNA3.3-TOPO (Addgene) to generate caspase-8 knockout cells. Caspase-10 knockout cells were generated by the use of the pSpCas9(BB)-2A-GFP (PX458) plasmid (Addgene). gRNA insertion was performed as previously described (Ran et al., 2013). gRNA sequences were designed using the open access software provided at http://crispr.mit.edu/ to target the 5′ end of the gene and thus all isoforms of either caspase-8 or 10. The gRNA sequences were as follows:

Casp8-1: GCCTGGACTACATTCCGCAA
Casp8-2: GCTCTTCCGAATTAATAGAC
Casp10: GGGGGTCCAAGATGTGGAGA

Two days post-transfection, cells were sorted with a BD FACSAria I (BD Biosciences) and single clones isolated and analyzed for successful caspase-8/10 knockout.

Immunoprecipitation of the CD95 Receptor

For the precipitation of DISC-associated proteins, 1.5×10^7 cells were used. The procedure of CD95 precipitation was performed as described previously (Cullen et al., 2013). Equal amounts of precipitates were analyzed by western blotting as described above.

Reconstitution of the CD95 DISC

Complete reconstitutions were carried out essentially as described previously (Hughes et al., 2009). Recombinant procaspase-10a was produced by in vitro transcription/translation (TNT T7-coupled reticulocyte lysate system; Promega) using pEF6/V5-His-TOPO vector containing caspase-10a or its respective ASM.

Real-Time qPCR

RNA isolation, cDNA synthesis, primer design, and real-time qPCR as well as data analysis were performed as described previously (Feoktistova et al., 2011). Data shown have been normalized to *GAPDH* and have been confirmed by normalization to *18S*.

Microarray Analysis

HeLa cells were seeded in 6-well plates, and respective shRNA expression was induced by the addition of 0.5 μg/mL doxycycline for 72 hr. In three independent experiments, cells were prestarved for 4 hr with media containing 0.5% fetal calf serum (FCS) followed by zVAD-fmk treatment (10 μM) for 1 hr. Cells were then stimulated with 0.1 U/mL CD95L for 3 hr. Total RNA from stimulated or control cells was isolated and tested by capillary electrophoresis on an Agilent 2100 bioanalyzer (Agilent Technologies) to confirm RNA quality. Gene expression profiling was performed using arrays of human Hugene-2_0-st-type from Affymetrix according to manufacturer's instructions. Bioinformatic evaluations were done as previously described (Czymai et al., 2010). Significant regulated genes (adjusted p values [false discovery rate (FDR)] < 0.05) were considered by a fold change >2 compared to unstimulated control cells.

Statistics

Statistical analysis was carried out using GraphPad Prism. Statistical significance (p values) was analyzed using either paired Student's t test (two-tailed) or two-way ANOVA, including Dunnett corrections for multiple comparisons, as indicated in the figure legends.

ACCESSION NUMBERS

The accession number for the microarray dataset reported in this paper is GEO: GSE75365.

SUPPLEMENTAL INFORMATION

Supplemental Information includes Supplemental Experimental Procedures, six figures, and one table and can be found with this article online at http://dx.doi.org/10.1016/j.celrep.2017.04.010.

AUTHOR CONTRIBUTIONS

S.H. designed and carried out most of the experiments. R.S. and M.P. performed essential additional experiments. C.S. performed and supervised microarray experiments and analyzed the resulting data. T.T. and P.M. initiated generation of CRISPR/Cas9 knockout cell lines and advised the respective experiments. M.A.H. and M.M. advised and performed the reconstituted DISC analysis. M.M., P.M., and M.R.S. discussed data throughout the study and advised experiments. M.L. conceptualized and initiated the study, obtained funding, designed experiments, and oversaw the study. S.H. and M.L. wrote the paper, with input from M.M., P.M., M.R.S., and M.A.H., particularly during the revision process. All authors reviewed the manuscript.

ACKNOWLEDGMENTS

With heavy hearts, we have to acknowledge Martin Leverkus, who tragically passed away while this manuscript was under revision. We are grateful for each dialogue and the time we spent together. It was a pleasure to know a great scientist like him. We furthermore thank Kelvin Cain, Michael Boutros, Henning Walczak, and the members of the Section of Molecular Dermatology, Medical Faculty Mannheim, University of Heidelberg, as well as the Scientists of the Department of Dermatology, RWTH Aachen, for constructive discussions. We are grateful to P.H. Krammer for monoclonal antibodies (mAbs) to caspase-8 (C-15), cFLIP (NF-6), and CD95 (Apo-1). Work in the laboratory of M.L. was funded by EU Horizon 2020 (MelPlex ESR network; project 5) and grants of the Deutsche Forschungsgemeinschaft (Le 953/6-1 and 953/8-1). M.L. was supported by Deutsche Forschungsgemeinschaft RTG 2099 (projects 9 and 10). Project 9 supports R.S., and project 10 provides funding for S.H. M.R.S. is supported by the Dietmar Hopp Foundation. M.M. and M.A.H. are supported by the UK Medical Research Council.

Received: January 12, 2016
Revised: May 30, 2016
Accepted: April 3, 2017
Published: April 25, 2017

REFERENCES

Barnhart, B.C., Legembre, P., Pietras, E., Bubici, C., Franzoso, G., and Peter, M.E. (2004). CD95 ligand induces motility and invasiveness of apoptosis-resistant tumor cells. EMBO J. *23*, 3175–3185.

Boatright, K.M., Deis, C., Denault, J.-B., Sutherlin, D.P., and Salvesen, G.S. (2004). Activation of caspases-8 and -10 by FLIP(L). Biochem. J. *382*, 651–657.

Brumatti, G., Ma, C., Lalaoui, N., Nguyen, N.Y., Navarro, M., Tanzer, M.C., Richmond, J., Ghisi, M., Salmon, J.M., Silke, N., et al. (2016). The caspase-8 inhibitor emricasan combines with the SMAC mimetic birinapant to induce necroptosis and treat acute myeloid leukemia. Sci. Transl. Med. *8*, 339ra69.

Chai, E.Z.P., Siveen, K.S., Shanmugam, M.K., Arfuso, F., and Sethi, G. (2015). Analysis of the intricate relationship between chronic inflammation and cancer. Biochem. J. *468*, 1–15.

Chan, F.K., Chun, H.J., Zheng, L., Siegel, R.M., Bui, K.L., and Lenardo, M.J. (2000). A domain in TNF receptors that mediates ligand-independent receptor assembly and signaling. Science *288*, 2351–2354.

Chen, L., Park, S.M., Tumanov, A.V., Hau, A., Sawada, K., Feig, C., Turner, J.R., Fu, Y.X., Romero, I.L., Lengyel, E., and Peter, M.E. (2010). CD95 promotes tumour growth. Nature *465*, 492–496.

Choi, C., Xu, X., Oh, J.-W., Lee, S.J., Gillespie, G.Y., Park, H., Jo, H., and Benveniste, E.N. (2001). Fas-induced expression of chemokines in human glioma cells: involvement of extracellular signal-regulated kinase 1/2 and p38 mitogen-activated protein kinase. Cancer Res. *61*, 3084–3091.

Cullen, S.P., Henry, C.M., Kearney, C.J., Logue, S.E., Feoktistova, M., Tynan, G.A., Lavelle, E.C., Leverkus, M., and Martin, S.J. (2013). Fas/CD95-induced chemokines can serve as "find-me" signals for apoptotic cells. Mol. Cell *49*, 1034–1048.

Czymai, T., Viemann, D., Sticht, C., Molema, G., Goebeler, M., and Schmidt, M. (2010). FOXO3 modulates endothelial gene expression and function by classical and alternative mechanisms. J. Biol. Chem. *285*, 10163–10178.

Dickens, L.S., Boyd, R.S., Jukes-Jones, R., Hughes, M.A., Robinson, G.L., Fairall, L., Schwabe, J.W.R., Cain, K., and MacFarlane, M. (2012). A death effector domain chain DISC model reveals a crucial role for caspase-8 chain assembly in mediating apoptotic cell death. Mol. Cell *47*, 291–305.

Diessenbacher, P., Hupe, M., Sprick, M.R., Kerstan, A., Geserick, P., Haas, T.L., Wachter, T., Neumann, M., Walczak, H., Silke, J., and Leverkus, M. (2008). NF-kappaB inhibition reveals differential mechanisms of TNF versus TRAIL-induced apoptosis upstream or at the level of caspase-8 activation independent of cIAP2. J. Invest. Dermatol. *128*, 1134–1147.

Dohrman, A., Kataoka, T., Cuenin, S., Russell, J.Q., Tschopp, J., and Budd, R.C. (2005a). Cellular FLIP (long form) regulates CD8+ T cell activation through caspase-8-dependent NF-kappa B activation. J. Immunol. *174*, 5270–5278.

Dohrman, A., Russell, J.Q., Cuenin, S., Fortner, K., Tschopp, J., and Budd, R.C. (2005b). Cellular FLIP long form augments caspase activity and death of T cells through heterodimerization with and activation of caspase-8. J. Immunol. *175*, 311–318.

Eckhart, L., Ballaun, C., Hermann, M., VandeBerg, J.L., Sipos, W., Uthman, A., Fischer, H., and Tschachler, E. (2008). Identification of novel mammalian caspases reveals an important role of gene loss in shaping the human caspase repertoire. Mol. Biol. Evol. *25*, 831–841.

Engels, I.H., Totzke, G., Fischer, U., Schulze-Osthoff, K., and Jänicke, R.U. (2005). Caspase-10 sensitizes breast carcinoma cells to TRAIL-induced but not tumor necrosis factor-induced apoptosis in a caspase-3-dependent manner. Mol. Cell. Biol. *25*, 2808–2818.

Farley, S.M., Purdy, D.E., Ryabinina, O.P., Schneider, P., Magun, B.E., and Iordanov, M.S. (2008). Fas ligand-induced proinflammatory transcriptional responses in reconstructed human epidermis. Recruitment of the epidermal

growth factor receptor and activation of MAP kinases. J. Biol. Chem. *283*, 919–928.

Feoktistova, M., Geserick, P., Kellert, B., Dimitrova, D.P., Langlais, C., Hupe, M., Cain, K., MacFarlane, M., Häcker, G., and Leverkus, M. (2011). cIAPs block ripoptosome formation, a RIP1/caspase-8 containing intracellular cell death complex differentially regulated by cFLIP isoforms. Mol. Cell *43*, 449–463.

Feoktistova, M., Geserick, P., Panayotova-Dimitrova, D., and Leverkus, M. (2012). Pick your poison: the ripoptosome, a cell death platform regulating apoptosis and necroptosis. Cell Cycle *11*, 460–467.

Fischer, U., Stroh, C., and Schulze-Osthoff, K. (2006). Unique and overlapping substrate specificities of caspase-8 and caspase-10. Oncogene *25*, 152–159.

Geserick, P., Hupe, M., Moulin, M., Wong, W.W.-L., Feoktistova, M., Kellert, B., Gollnick, H., Silke, J., and Leverkus, M. (2009). Cellular IAPs inhibit a cryptic CD95-induced cell death by limiting RIP1 kinase recruitment. J. Cell Biol. *187*, 1037–1054.

Harper, N., Farrow, S.N., Kaptein, A., Cohen, G.M., and MacFarlane, M. (2001). Modulation of tumor necrosis factor apoptosis-inducing ligand-induced NF-κ B activation by inhibition of apical caspases. J. Biol. Chem. *276*, 34743–34752.

Hughes, M.A., Harper, N., Butterworth, M., Cain, K., Cohen, G.M., and MacFarlane, M. (2009). Reconstitution of the death-inducing signaling complex reveals a substrate switch that determines CD95-mediated death or survival. Mol. Cell *35*, 265–279.

Hughes, M.A., Powley, I.R., Jukes-Jones, R., Horn, S., Feoktistova, M., Fairall, L., Schwabe, J.W.R., Leverkus, M., Cain, K., and MacFarlane, M. (2016). Co-operative and hierarchical binding of c-FLIP and caspase-8: a unified model defines how c-FLIP isoforms differentially control cell fate. Mol. Cell *61*, 834–849.

Irmler, M., Thome, M., Hahne, M., Schneider, P., Hofmann, K., Steiner, V., Bodmer, J.-L., Schröter, M., Burns, K., Mattmann, C., et al. (1997). Inhibition of death receptor signals by cellular FLIP. Nature *388*, 190–195.

Kavuri, S.M., Geserick, P., Berg, D., Dimitrova, D.P., Feoktistova, M., Siegmund, D., Gollnick, H., Neumann, M., Wajant, H., and Leverkus, M. (2011). Cellular FLICE-inhibitory protein (cFLIP) isoforms block CD95- and TRAIL death receptor-induced gene induction irrespective of processing of caspase-8 or cFLIP in the death-inducing signaling complex. J. Biol. Chem. *286*, 16631–16646.

Kischkel, F.C., Hellbardt, S., Behrmann, I., Germer, M., Pawlita, M., Krammer, P.H., and Peter, M.E. (1995). Cytotoxicity-dependent APO-1 (Fas/CD95)-associated proteins form a death-inducing signaling complex (DISC) with the receptor. EMBO J. *14*, 5579–5588.

Kischkel, F.C., Lawrence, D.A., Tinel, A., LeBlanc, H., Virmani, A., Schow, P., Gazdar, A., Blenis, J., Arnott, D., and Ashkenazi, A. (2001). Death receptor recruitment of endogenous caspase-10 and apoptosis initiation in the absence of caspase-8. J. Biol. Chem. *276*, 46639–46646.

Kreuz, S., Siegmund, D., Rumpf, J.-J., Samel, D., Leverkus, M., Janssen, O., Häcker, G., Dittrich-Breiholz, O., Kracht, M., Scheurich, P., and Wajant, H. (2004). NFkappaB activation by Fas is mediated through FADD, caspase-8, and RIP and is inhibited by FLIP. J. Cell Biol. *166*, 369–380.

Lamy, L., Ngo, V.N., Emre, N.C.T., Shaffer, A.L., 3rd, Yang, Y., Tian, E., Nair, V., Kruhlak, M.J., Zingone, A., Landgren, O., and Staudt, L.M. (2013). Control of autophagic cell death by caspase-10 in multiple myeloma. Cancer Cell *23*, 435–449.

Leverkus, M., Neumann, M., Mengling, T., Rauch, C.T., Bröcker, E.-B., Krammer, P.H., and Walczak, H. (2000). Regulation of tumor necrosis factor-related apoptosis-inducing ligand sensitivity in primary and transformed human keratinocytes. Cancer Res. *60*, 553–559.

Leverkus, M., Sprick, M.R., Wachter, T., Denk, A., Bröcker, E.-B., Walczak, H., and Neumann, M. (2003). TRAIL-induced apoptosis and gene induction in HaCaT keratinocytes: differential contribution of TRAIL receptors 1 and 2. J. Invest. Dermatol. *121*, 149–155.

Li, H., Zhu, H., Xu, C.J., and Yuan, J. (1998). Cleavage of BID by caspase 8 mediates the mitochondrial damage in the Fas pathway of apoptosis. Cell *94*, 491–501.

Michalak, P. (2008). Coexpression, coregulation, and cofunctionality of neighboring genes in eukaryotic genomes. Genomics *91*, 243–248.

Micheau, O., and Tschopp, J. (2003). Induction of TNF receptor I-mediated apoptosis via two sequential signaling complexes. Cell *114*, 181–190.

Micheau, O., Thome, M., Schneider, P., Holler, N., Tschopp, J., Nicholson, D.W., Briand, C., and Grütter, M.G. (2002). The long form of FLIP is an activator of caspase-8 at the Fas death-inducing signaling complex. J. Biol. Chem. *277*, 45162–45171.

Mühlethaler-Mottet, A., Flahaut, M., Bourloud, K.B., Nardou, K., Coulon, A., Liberman, J., Thome, M., and Gross, N. (2011). Individual caspase-10 isoforms play distinct and opposing roles in the initiation of death receptor-mediated tumour cell apoptosis. Cell Death Dis. *2*, e125.

Oberst, A., Pop, C., Tremblay, A.G., Blais, V., Denault, J.-B., Salvesen, G.S., and Green, D.R. (2010). Inducible dimerization and inducible cleavage reveal a requirement for both processes in caspase-8 activation. J. Biol. Chem. *285*, 16632–16642.

Park, D.R., Thomsen, A.R., Frevert, C.W., Pham, U., Skerrett, S.J., Kiener, P.A., and Liles, W.C. (2003). Fas (CD95) induces proinflammatory cytokine responses by human monocytes and monocyte-derived macrophages. J. Immunol. *170*, 6209–6216.

Peter, M.E., Budd, R.C., Desbarats, J., Hedrick, S.M., Hueber, A.-O., Newell, M.K., Owen, L.B., Pope, R.M., Tschopp, J., Wajant, H., et al. (2007). The CD95 receptor: apoptosis revisited. Cell *129*, 447–450.

Ran, F.A., Hsu, P.D., Wright, J., Agarwala, V., Scott, D.A., and Zhang, F. (2013). Genome engineering using the CRISPR-Cas9 system. Nat. Protoc. *8*, 2281–2308.

Sakamaki, K., Imai, K., Tomii, K., and Miller, D.J. (2015). Evolutionary analyses of caspase-8 and its paralogs: Deep origins of the apoptotic signaling pathways. BioEssays *37*, 767–776.

Schleich, K., Warnken, U., Fricker, N., Oztürk, S., Richter, P., Kammerer, K., Schnölzer, M., Krammer, P.H., and Lavrik, I.N. (2012). Stoichiometry of the CD95 death-inducing signaling complex: experimental and modeling evidence for a death effector domain chain model. Mol. Cell *47*, 306–319.

Schleich, K., Buchbinder, J.H., Pietkiewicz, S., Kähne, T., Warnken, U., Öztürk, S., Schnölzer, M., Naumann, M., Krammer, P.H., and Lavrik, I.N. (2016). Molecular architecture of the DED chains at the DISC: regulation of procaspase-8 activation by short DED proteins c-FLIP and procaspase-8 prodomain. Cell Death Differ. *23*, 681–694.

Schmidt, J.H., Pietkiewicz, S., Naumann, M., and Lavrik, I.N. (2015). Quantification of CD95-induced apoptosis and NF-κB activation at the single cell level. J. Immunol. Methods *423*, 12–17.

Siegmund, D., Hadwiger, P., Pfizenmaier, K., Vornlocher, H.-P., and Wajant, H. (2002). Selective inhibition of FLICE-like inhibitory protein expression with small interfering RNA oligonucleotides is sufficient to sensitize tumor cells for TRAIL-induced apoptosis. Mol. Med. *8*, 725–732.

Sprick, M.R., Weigand, M.A., Rieser, E., Rauch, C.T., Juo, P., Blenis, J., Krammer, P.H., and Walczak, H. (2000). FADD/MORT1 and caspase-8 are recruited to TRAIL receptors 1 and 2 and are essential for apoptosis mediated by TRAIL receptor 2. Immunity *12*, 599–609.

Sprick, M.R., Rieser, E., Stahl, H., Grosse-Wilde, A., Weigand, M.A., and Walczak, H. (2002). Caspase-10 is recruited to and activated at the native TRAIL and CD95 death-inducing signalling complexes in a FADD-dependent manner but can not functionally substitute caspase-8. EMBO J. *21*, 4520–4530.

Tenev, T., Bianchi, K., Darding, M., Broemer, M., Langlais, C., Wallberg, F., Zachariou, A., Lopez, J., MacFarlane, M., Cain, K., and Meier, P. (2011). The ripoptosome, a signaling platform that assembles in response to genotoxic stress and loss of IAPs. Mol. Cell *43*, 432–448.

van Raam, B.J., and Salvesen, G.S. (2012). Proliferative versus apoptotic functions of caspase-8 Hetero or homo: the caspase-8 dimer controls cell fate. Biochim. Biophys. Acta *1824*, 113–122.

Wachmann, K., Pop, C., van Raam, B.J., Drag, M., Mace, P.D., Snipas, S.J., Zmasek, C., Schwarzenbacher, R., Salvesen, G.S., and Riedl, S.J. (2010). Activation and specificity of human caspase-10. Biochemistry *49*, 8307–8315.

Wachter, T., Sprick, M., Hausmann, D., Kerstan, A., McPherson, K., Stassi, G., Bröcker, E.-B., Walczak, H., and Leverkus, M. (2004). cFLIPL inhibits tumor necrosis factor-related apoptosis-inducing ligand-mediated NF-kappaB activation at the death-inducing signaling complex in human keratinocytes. J. Biol. Chem. *279*, 52824–52834.

Wallach, D., Varfolomeev, E.E., Malinin, N.L., Goltsev, Y.V., Kovalenko, A.V., and Boldin, M.P. (1999). Tumor necrosis factor receptor and Fas signaling mechanisms. Annu. Rev. Immunol. *17*, 331–367.

Wang, J., Chun, H.J., Wong, W., Spencer, D.M., and Lenardo, M.J. (2001). Caspase-10 is an initiator caspase in death receptor signaling. Proc. Natl. Acad. Sci. USA *98*, 13884–13888.

Yu, J.W., Jeffrey, P.D., and Shi, Y. (2009). Mechanism of procaspase-8 activation by c-FLIPL. Proc. Natl. Acad. Sci. USA *106*, 8169–8174.

Cell Reports, Volume *19*

Supplemental Information

Caspase-10 Negatively Regulates Caspase-8-Mediated Cell Death, Switching the Response to CD95L in Favor of NF-κB Activation and Cell Survival

Sebastian Horn, Michelle A. Hughes, Ramon Schilling, Carsten Sticht, Tencho Tenev, Michaela Ploesser, Pascal Meier, Martin R. Sprick, Marion MacFarlane, and Martin Leverkus

Supplemental Information

Caspase-10 is a negative regulator of caspase-8-mediated cell death, switching the response to CD95L in favour of NF-κB activation and cell survival

Sebastian Horn[1*], Michelle A Hughes[2], Ramon Schilling[1], Carsten Sticht[3], Tencho Tenev[4], Michaela Ploesser[1], Pascal Meier[4], Martin R. Sprick[5*], Marion MacFarlane[2*‡], Martin Leverkus[1,6†]

Inventory of Supplemental Information

Supplemental Figures S1-6 and Table SI:

Figure S1, related to Figure 1
Caspase-10 blocks CD95L-induced cell death

Figure S2, related to Figure 2
Cellular expression levels reflect the stoichiometry of caspase-10 in the DISC

Figure S3, related to Figure 2
Loss of caspase-10 and/or cFLIP promotes CD95L-induced cell death

Figure S4, related to Figure 4
Caspase-8 knockout prevents CD95L-induced cell death

Figure S5, related to Figure 6
QVD does not support CD95L-mediated gene induction and is a weak inhibitor of caspase-8-mediated cell death

Figure S6, related to Figure 6
Caspase-10 and -8 induce DISC-mediated gene expression

Table SI, related to Figure 6
Analysis of CD95L-mediated gene induction by microarray after knockdown of caspase-10

Supplemental Experimental Procedures

Supplemental References

Supplemental Figures

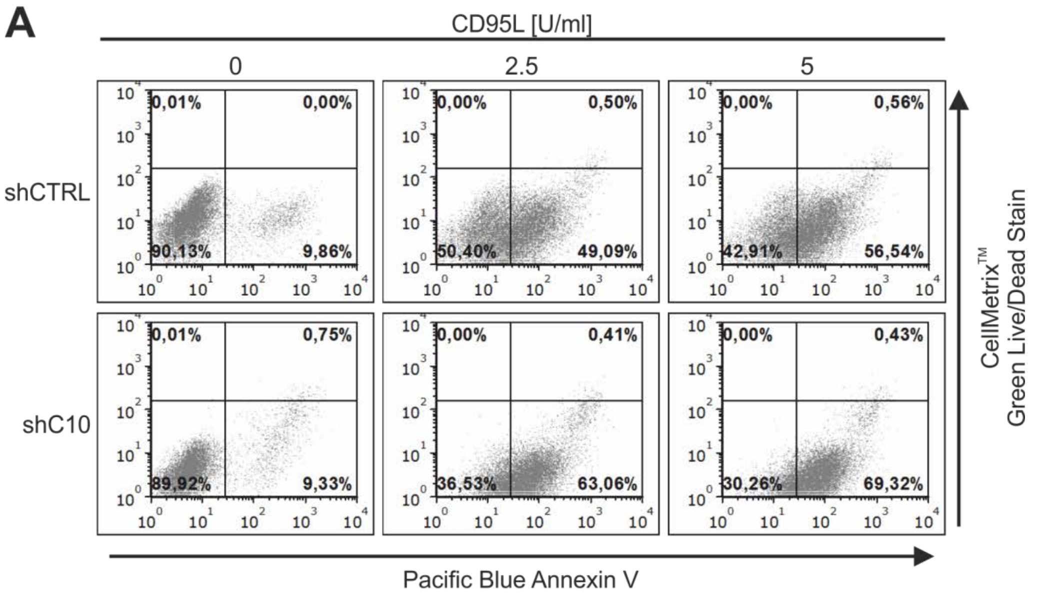

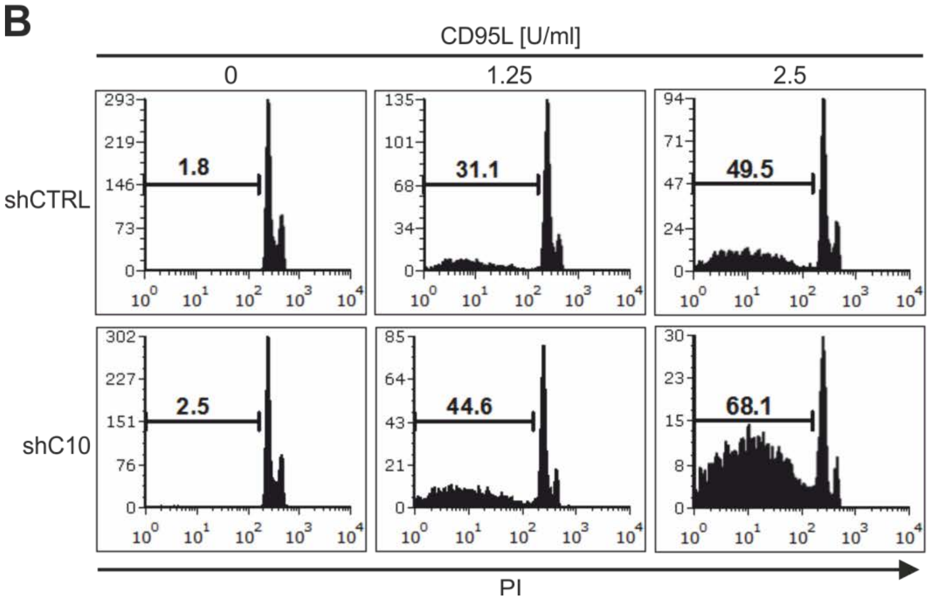

Figure S1, related to Figure 1. Caspase-10 blocks CD95L-induced cell death

(A) Cells were stimulated with the indicated concentrations of CD95L-Fc for 3 h. Apoptotic cells (Annexin V positive and CellMetrix negative) were measured by analyzing the externalization of phosphatidylserine and plasma membrane integrity by Pacific Blue Annexin V plus CellMetrix Green Live/Dead Stain using flow cytometry. A representative experiment is shown. (**B)** Cells were stimulated with the indicated CD95L-Fc concentrations for 7 h. DNA degradation was quantified by flow cytometry using PI staining for sub G1 populations. A representative experiment is shown.

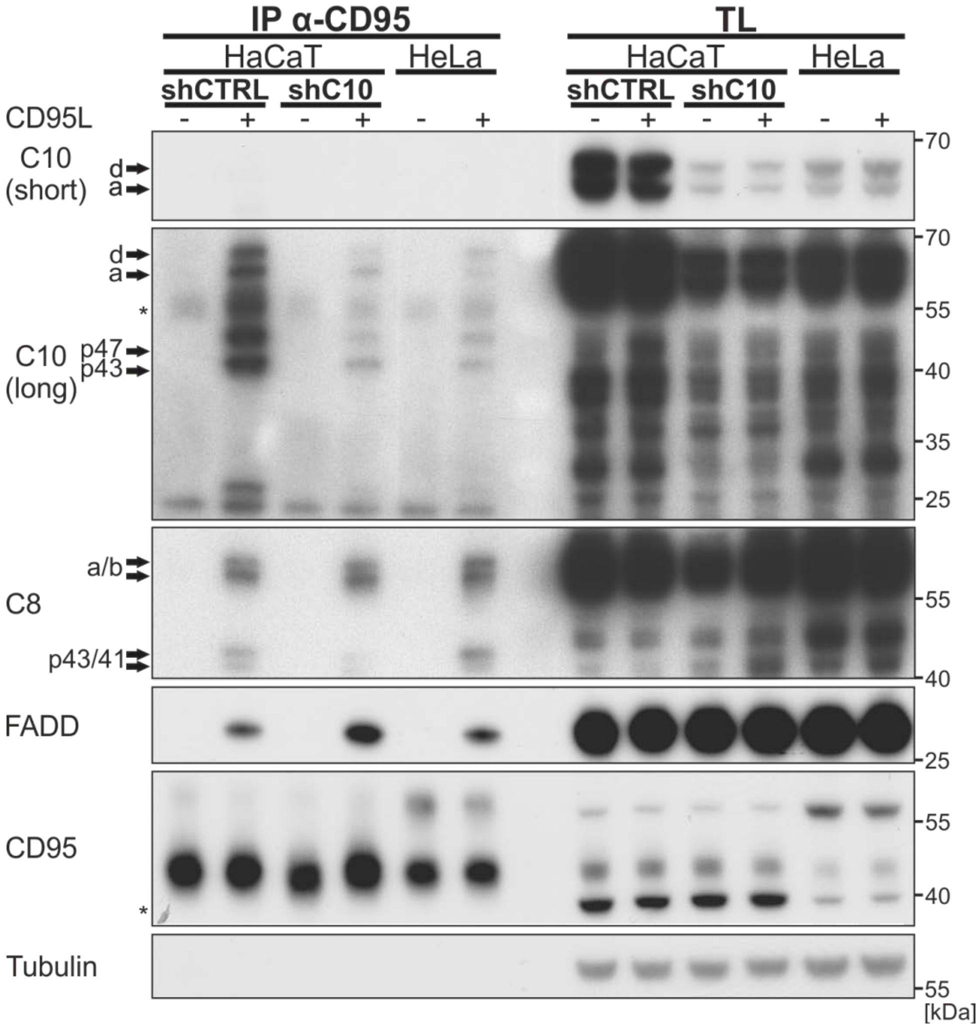

Figure S2, related to Figure 2. Cellular expression levels reflect the stoichiometry of caspase-10 in the DISC

Caspase-10 (shC10) or control (shCTRL) shRNA expression was induced in HaCaT cells by the addition of 0.5 µg/ml doxycycline for 72 h. Cells were stimulated with 1 U/ml CD95L-Fc for 40 min and compared to equivalently-treated HeLa cells. CD95 was immunoprecipitated from cell lysates (TL) and co-precipitated proteins were analyzed by Western blotting. The asterisks mark non-specific bands.

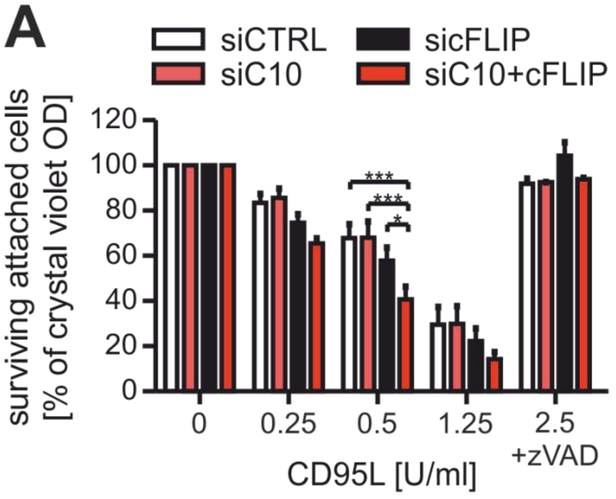

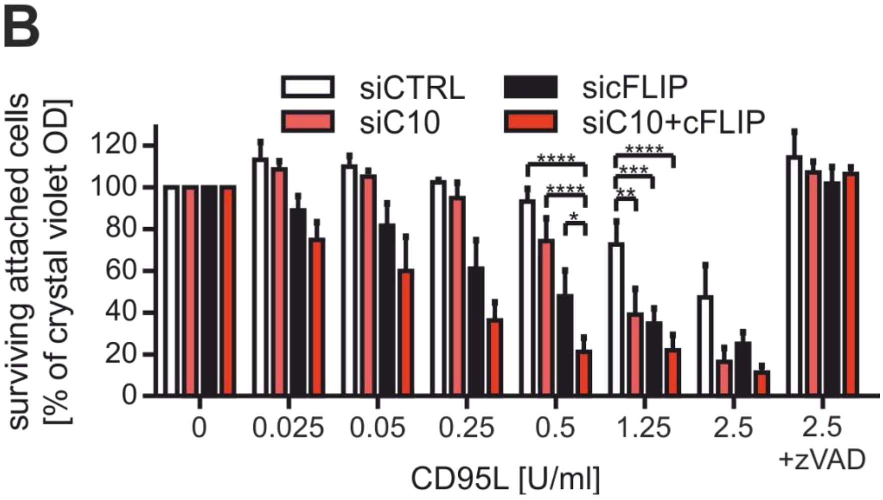

Figure S3, related to Figure 2. Loss of caspase-10 and/or cFLIP promotes CD95L-induced cell death

Dose-response to CD95L-Fc stimulation in HaCaT (**A**) and HeLa (**B**) cells after cFLIP and/or caspase-10 knockdown by siRNA. Cells were transfected with siC10, sicFLIP, the combination of both, or siCTRL. After 48 h, cells were pre-treated in triplicates with 10 µM zVAD for 1 h and further stimulated for 4 h with the indicated concentrations of CD95L-Fc, and cell viability analyzed by crystal violet staining. Each value represents mean ± SEM of three independent experiments. Significance levels (p values) were measured by two-way ANOVA test (*p<0.05; **p<0.01; ***p<0.001; ****p<0.0001).

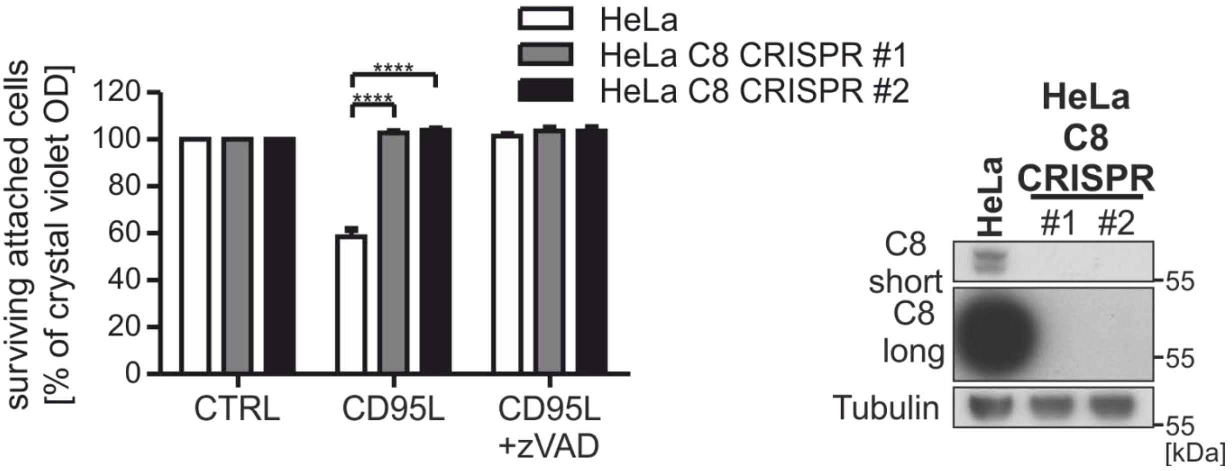

Figure S4, related to Figure 4. Caspase-8 knockout prevents CD95L-induced cell death

Caspase-8 deficient HeLa cell lines were generated using the CRISPR-Cas9 system with two different gRNAs targeting caspase-8. Parental HeLa cells as well as caspase-8 deficient cells (HeLa C8 CRISPR) were pre-treated with 10 µM zVAD-fmk (zVAD) for 1 h followed by stimulation with 1 U/ml CD95L-Fc for 16-20 h. Cell viability was analyzed in triplicates by crystal violet staining. Shown are mean values ± SEM of three independent experiments. Significance levels (p values) were measured by two-way ANOVA test (****p<0.0001). Knockout efficiency was measured by Western blotting.

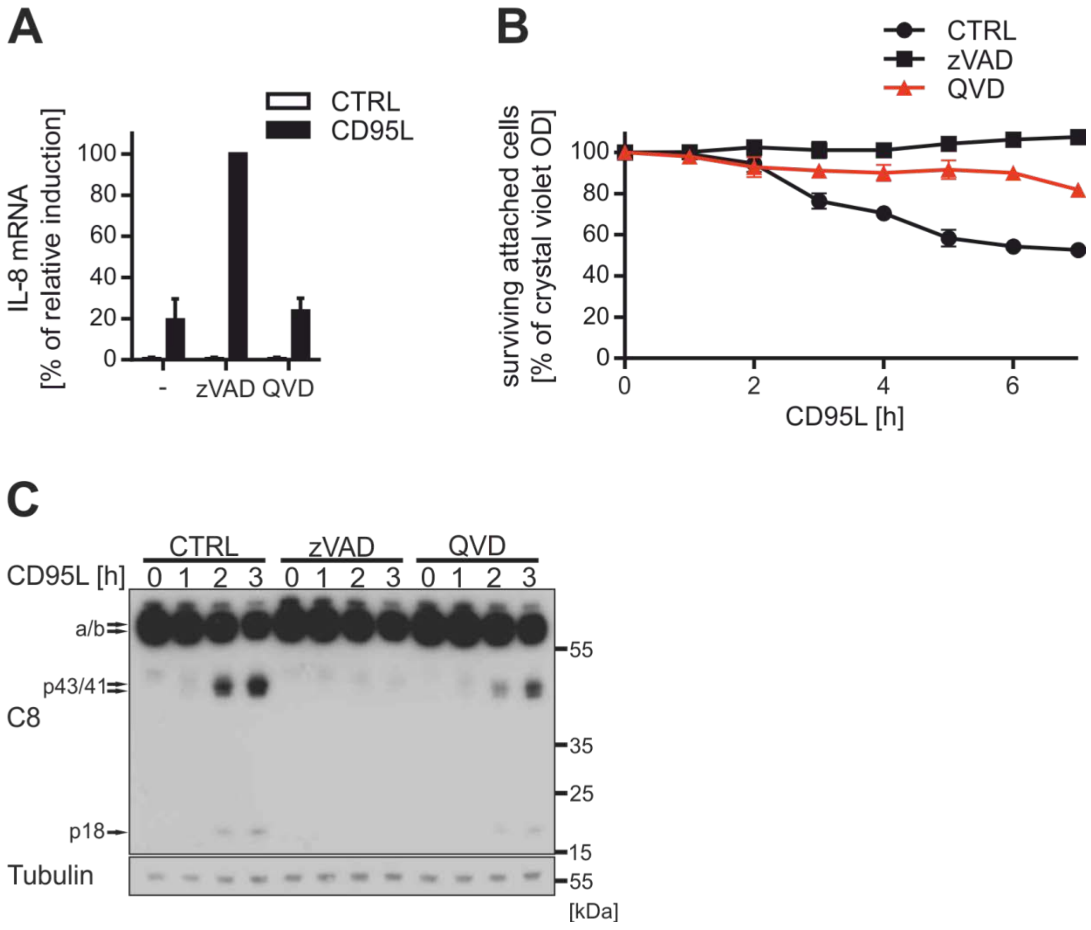

Figure S5, related to Figure 6. QVD does not support CD95L-mediated gene induction and is a weak inhibitor of caspase-8-mediated cell death

(**A**) HeLa+shCTRL cells were starved for 4 h in media containing 0.5 % FCS and pre-treated with 10 µM zVAD-fmk or 10 µM QVD-OPh for 1 h. Cells were stimulated with 0.1 U/ml CD95L-Fc for 3 h in the presence or absence of ZVAD-fmk or QVD-OPh. RNA was isolated, reverse transcribed to cDNA and mRNA expression levels of *IL-8* were analyzed by RT qPCR. Values shown are the average ± range of two independent experiments. **(B**) Triplicates of HeLa+shCTRL cells were pre-treated with 10 µM zVAD-fmk or 10 µM QVD-OPh and stimulated for the indicated time points with 0.1 U/ml CD95L-Fc. Cell viability was assayed by crystal violet staining. Shown are mean values of three technical replicates from one experiment. (**C**) HeLa+shCTRL cells were pre-stimulated as described in B and treated for the indicated time points with 0.1 U/ml CD95L-Fc. Total cell lysates were analyzed for caspase-8 processing by Western blotting.

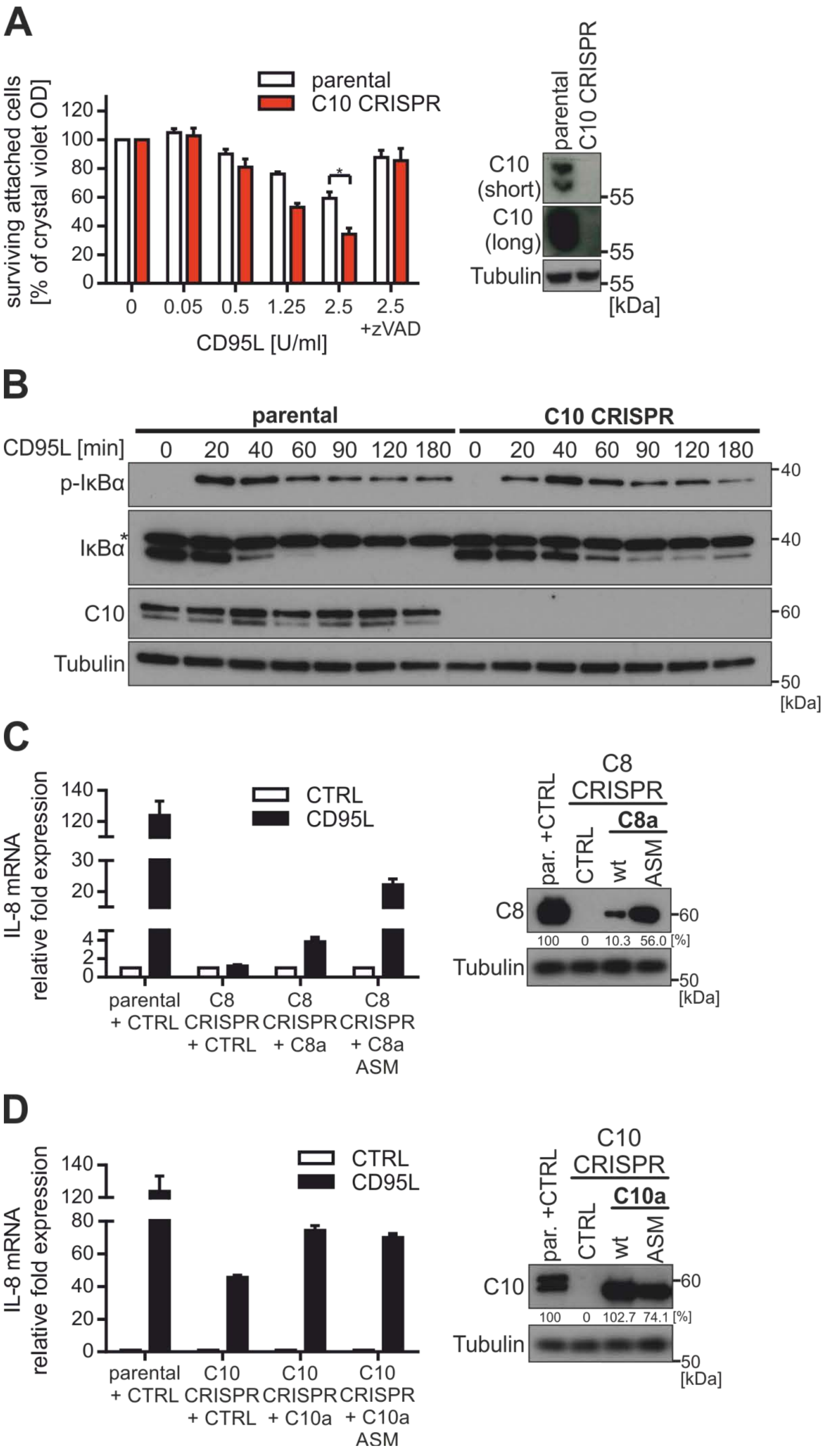
A
parental
C10 CRISPR
surviving attached cells
[% of crystal violet OD]
120
100
80
60
40
20
0
0
0.05
0.5
1.25
2.5
2.5
+zVAD
CD95L [U/ml]
*
parental
C10 CRISPR
C10
(short)
C10
(long)
Tubulin
55
55
55
[kDa]
B
parental
C10 CRISPR
CD95L [min]
0 20 40 60 90 120 180 0 20 40 60 90 120 180
p-IκBα
IκBα*
C10
Tubulin
40
40
60
50
[kDa]
C
CTRL
CD95L
IL-8 mRNA
relative fold expression
140
120
30
20
4
2
0
parental
+ CTRL
C8
CRISPR
+ CTRL
C8
CRISPR
+ C8a
C8
CRISPR
+ C8a
ASM
C8
CRISPR
par. +CTRL
CTRL
C8a
wt
ASM
C8
100
0
10.3
56.0
[%]
Tubulin
60
50
[kDa]
D
CTRL
CD95L
IL-8 mRNA
relative fold expression
140
120
80
60
40
20
0
parental
+ CTRL
C10
CRISPR
+ CTRL
C10
CRISPR
+ C10a
C10
CRISPR
+ C10a
ASM
C10
CRISPR
par. +CTRL
CTRL
C10a
wt
ASM
C10
100
0
102.7
74.1
[%]
Tubulin
60
50
[kDa]

Figure S6, related to Figure 6. Caspase-10 and -8 induce DISC-mediated gene expression

(**A**) Parental and caspase-10 deficient (C10 CRISPR) HeLa cells were pre-treated for 1 h with 10 µM zVAD-fmk (zVAD). Cells were stimulated in triplicate with the indicated concentrations of CD95L-Fc for 4 h. Cell death was analyzed by crystal violet staining. Caspase-10 knockout was controlled by Western blotting. (**B**) Parental and C10 CRISPR HeLa cells were starved for 4 h in media containing 0.5 % FCS, pretreated with 10 µM zVAD, and stimulated with 0.1 U/ml CD95L-Fc for the indicated time points. Caspase-10 knockout and IκBα degradation, as well as phosphorylation, were analyzed by Western blotting. The asterisk marks a non-specific band. (**C**) Raw data for Figure 6D (without the correction for caspase-8 expression levels). (**D**) Raw data for Figure 6E (without the correction for caspase-10 expression levels). Shown are mean values ± SEM of three independent experiments. Significance levels (p values) were measured by Student´s t-test (*$p<0.05$).

Table SI, related to Figure 6: Analysis of CD95L-mediated gene induction by microarray following knockdown of caspase-10

Gene symbol	log2 fold change (CD95L/CTRL)		Fold change (CD95L/CTRL)		p-values (CD95L/CTRL)		Relative repression of gene induction by shC10 [% of fold change]
	shCTRL	shC10	shCTRL	shC10	shCTRL	shC10	
CCL20	4.65	4.75	25.11	26.91	< 0.001	< 0.001	-7.2
IL8	4.57	4.17	23.75	18.00	< 0.001	< 0.001	24.2
PTX3	2.69	2.41	6.45	5.31	< 0.001	< 0.001	17.6
CCL2	2.09	1.80	4.26	3.48	0.015	0.031	18.2
IL6	2.07	1.71	4.20	3.27	< 0.001	0.001	22.1
CXCL3	2.03	1.76	4.08	3.39	0.012	0.022	17.1
EGR1	1.99	1.17	3.97	2.25	< 0.001	< 0.001	43.4
TNFAIP3	1.96	1.56	3.89	2.95	< 0.001	< 0.001	24.2
IL1A	1.85	1.53	3.61	2.89	0.001	0.005	19.9
NFKBIA	1.77	1.46	3.41	2.75	< 0.001	< 0.001	19.3
JUN	1.60	1.12	3.03	2.17	< 0.001	0.001	28.3
EFNA1	1.51	1.59	2.85	3.01	< 0.001	< 0.001	-5.7
IRF1	1.46	1.45	2.75	2.73	< 0.001	< 0.001	0.7
ICAM1	1.36	1.26	2.57	2.39	0.001	0.001	6.7
NFKBIE	1.16	0.79	2.23	1.73	< 0.001	0.002	22.6
TNF	1.16	0.61	2.23	1.53	0.001	0.031	31.7
OLR1	1.12	1.21	2.17	2.31	< 0.001	< 0.001	-6.4
DUSP5	1.08	0.91	2.11	1.88	< 0.001	< 0.001	11.1
NUAK2	1.06	1.05	2.08	2.07	< 0.001	< 0.001	0.7
NR4A2	1.04	0.76	2.06	1.69	< 0.001	0.002	17.6
KDM6B	1.03	0.93	2.04	1.91	0.003	0.007	6.7
SOD2	1.02	1.11	2.03	2.16	< 0.001	< 0.001	-6.4
IER5	1.01	0.69	2.01	1.61	0.002	0.020	19.9
CSF1	1.00	0.99	2.00	1.99	0.005	0.005	0.7

Dark orange: Significant reduction in gene expression (log2 fold change CD95L (shC10/shCTRL) < -0.5)
Light orange: Relative gene repression by shC10 > 15 %

Supplemental Experimental Procedures

Cell Lines

The cell lines, HeLa (cervical carcinoma, kindly provided by Dr. Michael Boutros), HaCaT (spontaneously transformed keratinocytes, generously provided by Dr. Petra Boukamp), SK-Mel and MC (melanoma) were cultured in DMEM high glucose + GlutaMAX^{TM} (Gibco, supplemented with 10 % FCS, 1 % Hepes, and 1 % sodium pyruvate) and maintained at 5 % CO_2 and 37 °C. Cell lines were cultured as previously described (Feoktistova et al., 2011; Geserick et al., 2015, 2009).

Materials

The following antibodies were used for Western blotting: antibodies to caspase-8 (C-15; kindly provided by P.H. Krammer), caspase-10 (4C1; MBL, Nagoya, Japan), cFLIP (NF-6; Alexis, San Diego, CA, USA), FADD (Clone 1; BD Transduction Laboratories™, San Diego, CA, USA); CD95 (C20; Santa Cruz, Delaware Avenue, CA, USA), IκBα (C-21; Santa Cruz, Delaware Avenue, CA, USA), phospho-IκBα (5A5; Cell Signaling Technology, Danvers, MA, USA), and β-tubulin (clone 2.1; Sigma-Aldrich, St.Louis, MO, USA). An expression construct for production of CD95L-Fc (Bossen et al., 2006) was kindly provided by P. Schneider (University of Lausanne, Epalinges, Switzerland). 1 U of CD95L-Fc was determined as 1 U/ml supernatant that was sufficient to kill 50% (LD50) of parental HeLa cells within 16-20 h. Ligand-mediated cell death was fully blocked by addition of soluble CD95-Fc protein. HRP-conjugated goat anti-rabbit, and goat anti–mouse IgG1 and IgG2b were obtained from SouthernBiotech (Birmingham, AL, USA). CD95 antibody for immunoprecipitation (Apo-1 IgG3) was kindly provided by P.H. Krammer (Dhein et al., 1992). Pacific Blue^{TM} Annexin V was from BioLegend (San Diego, CA, USA) and CellMetrix^{TM} Green Live/Dead Stain was purchased from ImmunoChemistry Technologies (Bloomington, MN, USA). 4-hydroytamoxifen, Necrostatin-1, cycloheximide, and propidium iodide were from Sigma-Aldrich (St. Louis, MO, USA). Doxycycline was purchased from Hexal (Holzkirchen, Germany), QVD-OPh from MP Biomedicals (Santa Ana, CA), and zVAD-fmk from Bachem (Bubendorf, Switzerland).

Transient Transfection of siRNA

For transient knockdown experiments the following siRNA duplexes were used: FlexiTube siRNA for caspase-8 (Hs_CASP8_11), caspase-10 (Hs_CASP10_8, Hs_CASP10_9, Hs-_CASP10_10, and Hs_CASP10_11), cFLIP (Hs_CFLAR_9) and the respective control siRNA (AllStars neg. control siRNA 1027281). All siRNA preparations were from QIAGEN (Venlo, Netherlands). For transient transfection $2x10^5$ cells per well were seeded in a 6-well plate and incubated overnight. Prior to transfection cells were incubated with Opti-MEM (Gibco 11058-021) medium for 20 min followed by transfection according to the

manufacturer´s recommendations using Lipofectamine 2000 (Thermo Fischer Scientific, Waltham, MA, USA) and the respective siRNA species. In case of single cFLIP knockdown, the molarity of cFLIP siRNA was reduced to 500 pM (HeLa) and 1 nM (HaCaT).

Cytotoxicity Assays

Crystal violet staining of attached living cells was performed after stimulations with the indicated concentrations and time points of CD95L-Fc in three technical replicates per condition in 96-well plates as previously described (Leverkus et al., 2000). Experiments were performed 3-5 times and the optical density of control conditions (cells treated with diluents) was normalized to 100 % to allow comparison of independent experiments. Subdiploid DNA content was analyzed as previously described (Diessenbacher et al., 2008) and measured by FACSCanto II analysis. To analyze the externalization of phosphatidylserine and plasma membrane integrity, $1x10^5$ HeLa cells were seeded per well in a 6-well plate and stimulated as described in the figure legend and further handled exactly as previously described (Geserick et al., 2009) with the exception that Pacific Blue Annexin V and CellMetrix Green Live/Dead Stain were used.

Western blotting

Cell lysates were prepared as previously described (Diessenbacher et al., 2008) and 10-15 µg of total cellular proteins were separated by SDS-PAGE on 4-12% gradient gels (Life Technologies, Carlsbad, CA) followed by transfer to PVDF membranes. Blocking of membranes and incubation with primary and appropriate secondary antibodies were essentially performed as described previously (Geserick et al., 2009). Bands were visualized with ECL detection kits (Luminata™ Forte Western HRP Substrate, Millipore, Schwalbach, Germany; Pierce ECL Western Blotting Substrate, Waltham, MA, USA). Respective protein bands were quantified by densitometry with *ImageJ* software using non-saturated exposures of blots.

Measurement of interleukin-8

$6x10^4$ HeLa cells were seeded per well in a 24-well plate and incubated over night at 37 °C. Interleukin-8 (IL-8) secretion was measured from cell free cell culture supernatants using the IL-8 ELISA kit obtained from BD Bioscience (Franklin Lakes, NJ, USA), and performed according to the manufacturer´s recommendations. Each assay was repeated at least three times and carried out using duplicate samples from each cell culture supernatant.

RT qPCR (primer sequences)

The following primers were used for the RT qPCR reaction:

IL-8

forward 5’- CAC CCC AAA TTT ATC AAA GA -3’;

reverse 5’- ACT GGC ATC TTC ACT GAT TC -3’;

TNF

forward 5’- TCA GAT CAT CTT CTC GAA CC -3’;

reverse 5’- TGG TTA TCT CTC AGC TCC AC -3´;

IL-6

forward 5’- ACT CAC CTC TTC AGA ACG AAT TG -3’;

reverse 5’- CCA TCT TTG GAA GGT TCA GGT TG -3´;

CCL2

forward 5’- CAG CCA GAT GCA ATC AAT GCC -3’;

reverse 5’- TGG AAT CCT GAA CCC ACT TCT -3´;

EGR1

forward 5’- GGT CAG TGG CCT AGT GAG C -3’;

reverse 5’- GTG CCG CTG AGT AAA TGG GA -3´;

CXCL3

forward 5’- CCA AAC CGA AGT CAT AGC CAC -3’;

reverse 5’- TGC TCC CCT TGT TCA GTA TCT -3´;

GAPDH

forward 5’- CCT GGT ATG ACA ACG AAT TT -3’;

reverse 5’- AGT GAG GGT CTC TCT CTT CC -3’;

18S

forward 5´- GAG GAT GAG GTG GAA CGT GT -3’;

reverse 5´- TCT TCA GTC GCT CCA GGT CT -3’.

Supplemental References

Bossen, C., Ingold, K., Tardivel, A., Bodmer, J.-L., Gaide, O., Hertig, S., Ambrose, C., Tschopp, J., Schneider, P., 2006. Interactions of tumor necrosis factor (TNF) and TNF receptor family members in the mouse and human. J. Biol. Chem. 281, 13964–13971.

Dhein, J., Daniel, P.T., Trauth, B.C., Oehm, A., Möller, P., Krammer, P.H., 1992. Induction of apoptosis by monoclonal antibody anti-APO-1 class switch variants is dependent on cross-linking of APO-1 cell surface antigens. J. Immunol. 149 , 3166–3173.

Diessenbacher, P., Hupe, M., Sprick, M.R., Kerstan, A., Geserick, P., Haas, T.L., Wachter, T., Neumann, M., Walczak, H., Silke, J., Leverkus, M., 2008. NF-[kappa]B Inhibition Reveals Differential Mechanisms of TNF Versus TRAIL-Induced Apoptosis Upstream or at the Level of Caspase-8 Activation Independent of cIAP2. J. Invest. Dermatol. 128, 1134–1147.

Feoktistova, M., Geserick, P., Kellert, B., Dimitrova, D.P., Langlais, C., Hupe, M., Cain, K., MacFarlane, M., Häcker, G., Leverkus, M., 2011. CIAPs Block Ripoptosome Formation, a RIP1/Caspase-8 Containing Intracellular Cell Death Complex Differentially Regulated by cFLIP Isoforms. Mol. Cell 43, 449–463.

Geserick, P., Hupe, M., Moulin, M., Wong, W.W.-L., Feoktistova, M., Kellert, B., Gollnick, H., Silke, J., Leverkus, M., 2009. Cellular IAPs inhibit a cryptic CD95-induced cell death by limiting RIP1 kinase recruitment. J. Cell Biol. 187, 1037–1054.

Geserick, P., Wang, J., Schilling, R., Horn, S., Harris, P.A., Bertin, J., Gough, P.J., Feoktistova, M., Leverkus, M., 2015. Absence of RIPK3 predicts necroptosis resistance in malignant melanoma. Cell Death Dis. 6, e1884.

Leverkus, M., Neumann, M., Mengling, T., Rauch, C.T., Bröcker, E.-B., Krammer, P.H., Walczak, H., 2000. Regulation of Tumor Necrosis Factor-related Apoptosis-inducing Ligand Sensitivity in Primary and Transformed Human Keratinocytes. Cancer Res. 60 , 553–559.
